# Comparative effectiveness of statins on non-high density lipoprotein cholesterol in people with diabetes and at risk of cardiovascular disease: systematic review and network meta-analysis

**DOI:** 10.1136/bmj-2021-067731

**Published:** 2022-03-24

**Authors:** Alexander Hodkinson, Dialechti Tsimpida, Evangelos Kontopantelis, Martin K Rutter, Mamas A Mamas, Maria Panagioti

**Affiliations:** 1 National Institute for Health Research School for Primary Care Research, Division of Population Health, Health Services Research and Primary Care, School of Health Sciences, Faculty of Biology, Medicine and Health, Manchester Academic Health Science Centre, University of Manchester, Manchester, UK; 2 Institute for Health Policy and Organisation, Faculty of Biology, Medicine and Health, Manchester Academic Health Science Centre, University of Manchester, Manchester, UK; 3 Division of Medical Education, School of Medical Sciences, Faculty of Biology, Medicine and Health, Manchester Academic Health Science Centre, University of Manchester, Manchester, UK; 4 Division of Informatics, Imaging, and Data Sciences, Faculty of Biology, Medicine and Health, Manchester Academic Health Science Centre, University of Manchester, Manchester, UK; 5 Division of Diabetes, Endocrinology, and Gastroenterology, School of Medical Sciences, Faculty of Biology, Medicine and Health, Manchester Academic Health Science Centre, University of Manchester, Manchester, UK; 6 Diabetes, Endocrinology, and Metabolism Centre, Manchester University NHS Foundation Trust, Manchester, UK; 7 Keele Cardiovascular Research Group, Centre for Prognosis Research, Keele University, Keele, UK; 8 Department of Cardiology, Royal Stoke University Hospital, Stoke-on-Trent, UK; 9 National Institute for Health Research Greater Manchester Patient Safety Translational Research Centre, Division of Population Health, Health Services Research and Primary Care, University of Manchester, Manchester, UK

## Abstract

**Objective:**

To compare the efficacy of different statin treatments by intensity on levels of non-high density lipoprotein cholesterol (non-HDL-C) for the prevention of cardiovascular disease in people with diabetes.

**Design:**

Systematic review and network meta-analysis.

**Data sources:**

Medline, Cochrane Central Register of Controlled Trials, and Embase from inception to 1 December 2021.

**Review methods:**

Randomised controlled trials comparing different types and intensities of statins, including placebo, in adults with type 1 or type 2 diabetes mellitus were included. The primary outcome was changes in levels of non-HDL-C, calculated from measures of total cholesterol and HDL-C. Secondary outcomes were changes in levels of low density lipoprotein cholesterol (LDL-C) and total cholesterol, three point major cardiovascular events (non-fatal stroke, non-fatal myocardial infarction, and death related to cardiovascular disease), and discontinuations because of adverse events. A bayesian network meta-analysis of statin intensity (low, moderate, or high) with random effects evaluated the treatment effect on non-HDL-C by mean differences and 95% credible intervals. Subgroup analysis of patients at greater risk of major cardiovascular events was compared with patients at low or moderate risk. The confidence in network meta-analysis (CINeMA) framework was applied to determine the certainty of evidence.

**Results:**

In 42 randomised controlled trials involving 20 193 adults, 11 698 were included in the meta-analysis. Compared with placebo, the greatest reductions in levels of non-HDL-C were seen with rosuvastatin at high (−2.31 mmol/L, 95% credible interval −3.39 to −1.21) and moderate (−2.27, −3.00 to −1.49) intensities, and simvastatin (−2.26, −2.99 to −1.51) and atorvastatin (−2.20, −2.69 to −1.70) at high intensity. Atorvastatin and simvastatin at any intensity and pravastatin at low intensity were also effective in reducing levels of non-HDL-C. In 4670 patients at greater risk of a major cardiovascular events, atorvastatin at high intensity showed the largest reduction in levels of non-HDL-C (−1.98, −4.16 to 0.26, surface under the cumulative ranking curve 64%). Simvastatin (−1.93, −2.63 to −1.21) and rosuvastatin (−1.76, −2.37 to −1.15) at high intensity were the most effective treatment options for reducing LDL-C. Significant reductions in non-fatal myocardial infarction were found for atorvastatin at moderate intensity compared with placebo (relative risk=0.57, confidence interval 0.43 to 0.76, n=4 studies). No significant differences were found for discontinuations, non-fatal stroke, and cardiovascular deaths.

**Conclusions:**

This network meta-analysis indicated that rosuvastatin, at moderate and high intensity doses, and simvastatin and atorvastatin, at high intensity doses, were most effective at moderately reducing levels of non-HDL-C in patients with diabetes. Given the potential improvement in accuracy in predicting cardiovascular disease when reduction in levels of non-HDL-C is used as the primary target, these findings provide guidance on which statin types and intensities are most effective by reducing non-HDL-C in patients with diabetes.

**Systematic review registration:**

PROSPERO CRD42021258819.

## Introduction

Type 2 diabetes is expected to affect 380 million people worldwide by 2025,^1^ ^2^ and patients with type 2 diabetes are at increased risk of cardiovascular diseases, the leading cause of death globally, with an estimated 17.9 million deaths each year.^3^ ^4^ Lipid modifying treatments, such as statins, are considered the basis of primary and secondary prevention of cardiovascular disease by lowering levels of low density lipoprotein cholesterol (LDL-C) in the blood.^5^ Statins have been found to be the most effective agents in reducing the risk of coronary heart disease in patients with diabetes, reducing the relative risk by a third.^6^ ^7^

The National Cholesterol Education Program in the United States recommends that LDL-C values should be used to estimate the risk of cardiovascular disease related to lipoproteins in individuals.^8^ Non-high density lipoprotein cholesterol (HDL-C), however, might be more strongly associated with the risk of cardiovascular disease in patients receiving statins,^9^ and could be a better tool than LDL-C for assessing the risk of cardiovascular disease and the effects of treatment.^10^ The rationale for this recommendation is that non-HDL-C includes all potentially atherogenic cholesterol present in lipoprotein particles, including LDL, lipoprotein (a), intermediate density lipoprotein, and very low density lipoprotein remnants.

Guidelines from the National Institute for Health and Care Excellence (NICE) for adults with diabetes were updated in April 2021. NICE now recommends that non-HDL-C should replace LDL-C as the primary target for reducing the risk of cardiovascular disease with lipid lowering treatment.^11^ In contrast, other international guidelines do not have a non-HDL-C target. The European Society of Cardiology uses LDL-C as their treatment goal.^12^ Similarly, the American College of Cardiology, American Heart Association, and National Lipid Association target reductions in LDL-C based on patient risk.^13^

Despite the potential of non-HDL-C as a predictor of developing cardiovascular diseases, no study has assessed the comparative effectiveness of different lipid lowering treatments on levels of non-HDL-C in people with diabetes. Therefore, we carried out a systematic review and network meta-analysis to estimate the comparative effectiveness of seven statins on levels of non-HDL-C in patients with diabetes.

## Methods

We undertook the systematic review and network meta-analysis according to a review protocol (PROSPERO CRD42021258819), and the results were reported in accordance with the Preferred Reporting Items for Systematic Reviews and Meta-Analyses (PRISMA) extension statement for network meta-analysis (PRISMA extension checklist, appendix 1).^14^

### Data sources and search strategies

Searches were performed from inception to 1 December 2021 in Medline, Cochrane Central Register of Controlled Trials, and Embase. Screening was done by two independent blinded reviewers (AH, DT) with Covidence software, and disagreements were resolved by a third reviewer (MP). Appendix 2 shows the full search strategy. Reference lists of included studies and relevant systematic reviews were screened for more studies. Trial registries (ClinicalTrials.gov, ISCTRN (International Standard Randomised Controlled Trial Number), WHO ICTRP (World Health Organization International Clinical Trials Registry Platform) portal, and OpenTrials.net) were also searched for unpublished or ongoing trials. Drug approval packages at the Food and Drug Administration and European Product Assessment Reports were also scanned for unpublished studies or relevant outcome data. We excluded studies not reported in English.

### Eligibility criteria

Studies of patients aged ≥18 years with a diagnosis of type 1 or 2 diabetes were eligible. We included patients treated for primary (that is, no diagnosis of cardiovascular disease) or secondary (that is, history of a cardiovascular disease according to the three point major adverse cardiovascular events classification) prevention of cardiovascular disease. The seven globally prescribed statins were atorvastatin (Lipitor), fluvastatin (Lescol), lovastatin (Altoprev), pitavastatin (Livalo, Zypitamag), pravastatin (Pravachol), rosuvastatin (Crestor, Ezallor), and simvastatin (Zocor) at any dose. The comparator was placebo or any of the seven statins. The primary outcome was a reduction in levels of non-HDL-C, but we also included studies reporting both total cholesterol and HDL-C, allowing us to calculate non-HDL-C levels. Secondary outcomes were reductions in levels of LDL-C and total cholesterol, classical three point major cardiovascular events (defined as non-fatal stroke, non-fatal myocardial infarction, and death related to cardiovascular disease),^15^ and discontinuations because of adverse event. Only randomised controlled trials were included to limit potential bias.

### Data extraction

We extracted data with a standardised form that was previously tested in a pilot study. Data included study characteristics (country, placebo controlled, length of follow-up, and number of patients and outcomes reported) and patient characteristics (mean or median age in years, percentage of men, ethnicity according to the definition of the Office for National Statistics for ethnic group, national identity and region,^16^ baseline body mass index, diabetes type (1 or 2), duration of diabetes, comorbidity, concomitant drug use (other lipid lowering treatments), and risk of patients according to major cardiovascular events). Data on interventions included the statin agent, dose in milligrams, and intensity based on guidelines from the American College of Cardiology/American Heart Association,^17^ European Society of Cardiology,^18^ and NICE.^19^ All data extractions were completed by two reviewers (AH, DT) and checked by another reviewer (MP).

### Categorisation of statin intensity

Based on the recommendations of the American College of Cardiology/American Heart Association, European Society of Cardiology, and NICE for assessing the risk of cardiovascular disease and the reduction in risk, including lipid modification, statins were grouped into three intensity categories according to the percentage reduction in levels of LDL-C: 20-30% reduction is low intensity; 31-39% reduction is medium intensity; and ≥40% reduction is high intensity.^20^ Table 1 shows the classification of the seven statins used in our analysis following the three dose intensity groups (low, moderate, and high) for the expected reduction in LDL-C. If the dose of statin was positioned between the range of two intensity groups, we chose the nearest group to ensure an intensity was assigned.

**Table 1:** Statin dosing and American College of Cardiology/American Heart Association, European Society of Cardiology, and National Institute for Health and Care Excellence classification of intensity according to percentage reduction in low density lipoprotein cholesterol (LDL-C)

Statin	Total daily dose, mg		
	Low intensity (LDL-C reduced by 20-30%)	Moderate intensity (LDL-C reduced by 31-39%)	High intensity (LDL-C reduced by ≥40%)
Atorvastatin	NA	10-20	40-80
Fluvastatin	20-40	80	NA
Lovastatin	20	40-80	NA
Pitavastatin	NA	1-4	NA
Pravastatin	10-20	40-80	NA
Rosuvastatin	NA	5-10	20-40
Simvastatin	10	20-40	80

NA=no classification available from any guideline.

### Quality assessment of evidence

The quality of the individual studies was assessed independently by two reviewers with the Cochrane risk of bias tool 2.0 for randomised controlled trials. The overall risk of bias was classified as: low (score=1), when a study was judged to be at low risk of bias for all domains with some concerns showing; some concerns (score=2), when the study was judged to raise more domains with at least some concerns or high risk of bias in one domain; or high (score=3), when the study was judged to be at high risk of bias in at least one domain or to have some concerns for multiple domains in a way that substantially lowered confidence in the result, or both.^21^ We also applied the CINeMA (confidence in network meta-analysis) framework^22^ ^23^ to assess the certainty of evidence covering six key domains: within study bias, reporting bias, indirectness, imprecision, heterogeneity, and incoherence.

### Data synthesis

The primary outcome of changes in levels of non-HDL-C was calculated as the net difference between levels of total cholesterol and HDL-C. The means and standard deviations of total cholesterol and HDL-C were converted from milligrams per decilitre (mg/dL) to millimoles per litre (mmol/L), the international standard measure for levels of cholesterol. Variance for these calculated values were determined with previously developed procedures.^24^

Net changes in levels of non-HDL-C were calculated as the difference (statin minus placebo or statin comparator) in these mean values in a network meta-analysis setting, which allowed for the simultaneous evaluation of the different statin intensities.^25^ To ensure transitivity within the network, we categorised all statin agents and intensity groups, and placebo, into nodes and compared the distribution of clinical (total cholesterol, HDL-C) and methodological (age, sex, and body mass index) variables.^26^ We used a bayesian random effects network meta-analysis model with a normal likelihood. To account for the correlations induced by multigroup studies, we used multivariate distributions. We considered the I^2^ statistic and the (heterogeneity) variance in the distribution of the random effect (τ^2^) to measure the extent of the influence of variability across and within studies on treatment effects. I^2^ statistic and the 95% confidence interval was interpreted as 0-29%, 30-59%, 60-89%, and >89%, indicating low, moderate, substantial, and high heterogeneity, respectively. To rank the treatments by efficacy, we used the surface under the cumulative ranking curve.^27^ We evaluated consistency (that is, agreement between direct and indirect evidence) by considering direct and indirect evidence separately with node splitting.^28^ ^29^

We fitted all models with the MBNMAdose (version 0.3.0) package^30^ in R version 4.0.5 (R Foundation for Statistical Computing). Specifically, we used uninformative prior distributions for the treatment effects and a minimally informative prior distribution for the common standard deviation parameter. Model convergence was ensured by visual inspection of three Markov chain Monte Carlo chains after considering the Brooks-Gelman-Rubin diagnostic. Network graphs scaled by the number of studies and patients by each treatment node were presented graphically. The GeMTC package in R was used to produce some figures and to check the results.^31^ ^32^ The secondary outcomes, changes in levels of LDL-C and total cholesterol, were analysed in the same way as non-HDL-C. We found few reports for the outcome, discontinuations because of adverse events, so we used the Peto odds ratio method, which is proven to be more suitable for meta-analysing rare events.^33^ The three point major cardiovascular event outcomes were analysed with DerSimonian and Laird pairwise meta-analysis with the relative risk.^34^

We performed a subgroup network meta-analysis for the non-HDL-C outcome that focused on high risk patients compared with patients at low to medium risk.^35^ With the inclusion and exclusion criteria, and baseline data from the individual trial reports, patient risk was categorised as: high, for those with a history of major cardiovascular event outcomes (that is, non-fatal stroke, non-fatal myocardial infarction, coronary heart disease, or cardiovascular disease)^36^; and low to medium, for those who had no previous or current major cardiovascular events at baseline.

A sensitivity analysis with the dose specific network model was conducted to examine the robustness of the findings from the analysis involving categorisation by intensity. A network funnel plot was used to visually scrutinise the criterion of symmetry and penitential presence for small study effect bias.^37^

### Patient and public involvement

In designing this study, we held a patient and public involvement focus group with 24 adults who had diabetes and were taking statins for prevention of cardiovascular disease, to help inform on the interpretation of our findings. These review results will be disseminated to the relevant patient communities.

## Results

**Fig 1:**
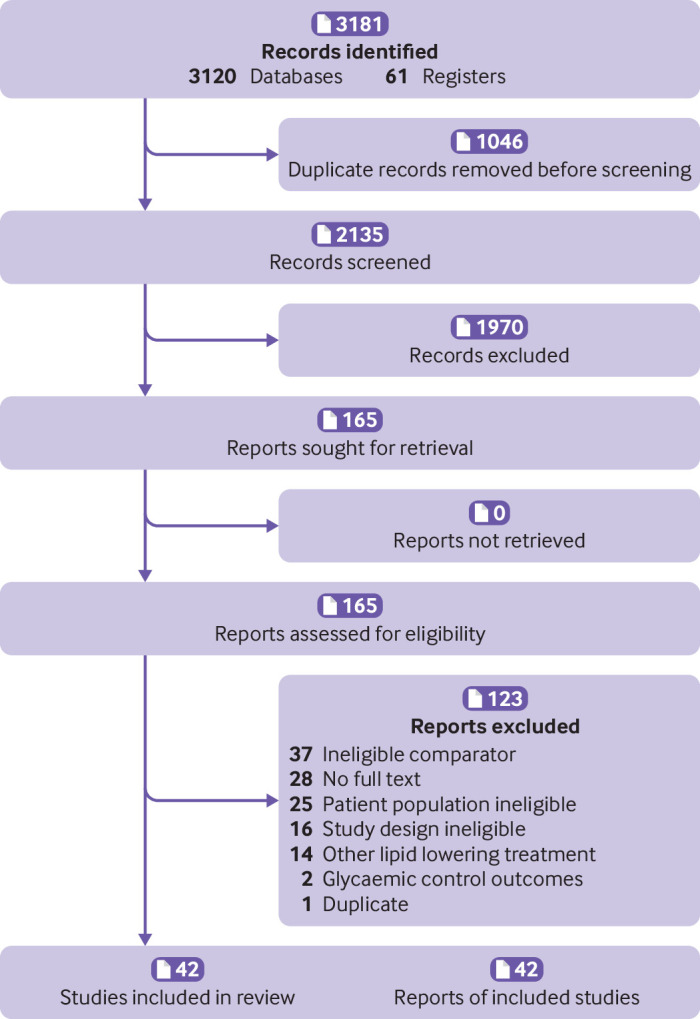
Preferred Reporting Items for Systematic Reviews and Meta-Analyses (PRISMA) flowchart

The search retrieved 3181 references. After screening the titles and abstracts of 2135 references, 1970 were excluded, and the full text of 165 reports were screened. Forty two randomised controlled trials, involving 20 193 participants, met our inclusion criteria (fig 1). Appendix 3 lists the included studies.

### Characteristic of included studies

Appendix 4 shows the characteristics of the included studies. Fourteen (33%) of the studies were carried out in the European Union, six (14%) in the US, and four (10%) in the UK.^6^ ^38^ ^39^ ^40^ The studies involved a median of 145 (range 52-390, interquartile range 338) patients with a median age of 60 years (58-62, 4). Eighteen (43%) studies involved 55% or more men, 11 (26%) involved 55% or more women, and 11 (26%) had a mixture of both sexes. Patients were mostly overweight, with a median body mass index at baseline of 29 (26-31, 5). Seventeen (40%) of the studies included Asian populations (South Korean (n=6),^41^ ^42^ ^43^ ^44^ ^45^ ^46^ Japanese (n=3),^47^ ^48^ ^49^ Taiwanese (n=3),^50^ ^51^ ^52^ Arabic (n=2),^53^ ^54^ Chinese (n=1),^55^ Indian (n=1),^56^ and Thai (n=1) 57), 12 (29%) included white ethnic groups (western or European),^6^ ^58^ ^59^ ^60^ ^61^ ^62^ ^63^ ^64^ ^65^ ^66^ ^67^ ^68^ and one (2%) study included patients of mixed ethnicity.^69^ In 12 of the studies (29%), ethnicity was not reported.^38^ ^40^ ^70^ ^71^ ^72^ ^73^ ^74^ ^75^ ^76^ ^77^ ^78^ ^79^

In 35 (83%) studies, patients had a diagnosis of type 2 diabetes, in five (12%), patients had a diagnosis of type 1 or 2 diabetes,^55^ ^70^ ^71^ ^79^ ^80^ and in two (5%) studies, patients had a diagnosis of type 1 diabetes only.^63^ ^74^ Of the 22 (49%) studies that reported the average length of the diagnosis of diabetes, the median was 8 years (range 4-11, interquartile range 7). Most studies (n=32, 76%) involved the secondary prevention of cardiovascular disease but nine (21%) studies targeted primary prevention; in one (2%) study, the target was unclear.^76^ Common comorbidities included stable hypercholesterolaemia (n=12), cardiovascular disease or cardiovascular risk factors (hypertension (n=6), coronary artery disease (n=3), coronary heart disease or peripheral vascular disease (n=3), acute myocardial infarction (n=3), or stable angina (n=2)), metabolic syndrome (n=1), and retinopathy (n=1). Eleven studies reported diabetes status only. In six of the studies, patients were taking other (concomitant) lipid lowering treatment at enrolment; in the remaining 36 studies, most had a washout phase before recruitment or did not specify use of lipid lowering treatment. Eighteen (43%) of the studies involved mostly patients at low risk, 12 (29%) involved patients at moderate risk, and 12 (29%) involved patients at high risk with a current diagnosis of a cardiovascular disease or a previous history of major cardiovascular events. Twenty four (57%) of the studies were placebo controlled and 18 studies (43%) involved an active statin treatment as the comparator. The median length of the intervention period in the studies was 12 weeks (range 8-24).

### Assessment of risk of bias

The quality of the studies varied (appendix 5). Five (12%) studies had a high risk of bias for the randomisation process, six (14%) had a high risk for deviations from the intended intervention, five (12%) had a high risk for missing outcome data, and 11 (26%) had a high risk for the measurement of the outcome domain. Selection reporting bias was found in seven (17%) of the studies. Overall, 19 studies (48%) had a low risk of bias (overall bias score of 1) and two of the studies (52%) had a score above 1, indicating some concerns or high risk of bias.

### Network meta-analysis

**Fig 2:**
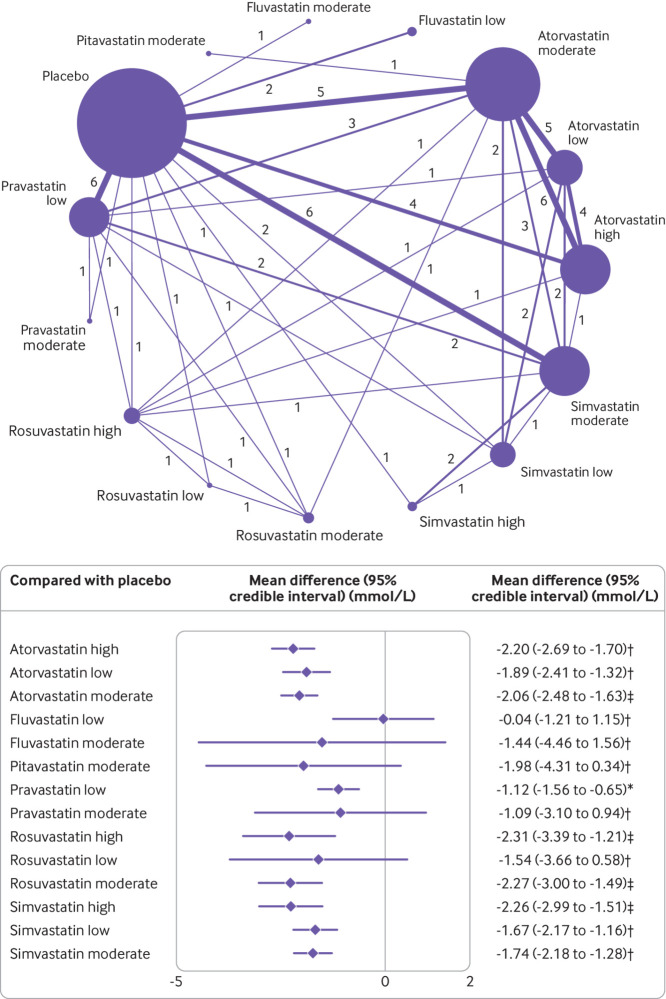
Network of available comparisons between statin intensities for non-high density lipoprotein cholesterol, and forest plot of network effect sizes of statin intensities compared with placebo. Size of node is proportional to number of trial participants, and thickness of line connecting nodes is proportional to number of trial participants randomised in trials directly comparing the two treatments. Numbers represent the number of trials contributing to each treatment comparison. Certainty of the evidence, according to the confidence in network meta-analysis (CINeMA) framework, is included in the forest plot and classified as *low, †moderate, and ‡high confidence of evidence. Appendix 11 shows the full CINeMA assessments

Figure 2 shows the network of eligible comparisons for the primary outcome, changes in levels of non-HDL-C, involving 36 of the trials with amenable data for including in the meta-analysis. The network of evidence included 15 interventions, 11 698 patients, 24 two arm studies, and 12 multi-arm studies. Twenty one studies involved atorvastatin (n=15 moderate intensity,^6^ ^42^ ^46^ ^49^ ^50^ ^51^ ^52^ ^53^ ^58^ ^59^ ^67^ ^71^ ^72^ ^73^ ^75^ n=10 high intensity,^40^ ^42^ ^46^ ^50^ ^51^ ^56^ ^59^ ^60^ ^66^ ^72^ and n=7 low intensity^46^ ^50^ ^51^ ^57^ ^58^ ^72^ ^76^), three studies involved fluvastatin (n=2 low intensity^47^ ^68^ and n=1 moderate intensity^77^), one study involved pitavastatin at moderate intensity,^52^ eight studies involved pravastatin (n=8 low intensity^41^ ^44^ ^49^ ^58^ ^63^ ^70^ ^73^ ^79^ and n=1 moderate intensity^41^), four studies involved rosuvastatin (n=3 high intensity,^45^ ^56^ ^58^ n=2 moderate intensity,^45^ ^49^ and n=1 low intensity^45^), and 14 studies involved simvastatin (n=10 moderate intensity,^43^ ^44^ ^50^ ^53^ ^54^ ^58^ ^65^ ^69^ ^74^ ^76^ n=5 low intensity,^43^ ^57^ ^71^ ^73^ ^76^ and n=2 high intensity^43^ ^65^). Appendix 6 shows the model fit statistics and the profile plots of treatment response by dose for the convergence model.

### Inconsistency analysis

We found evidence of statistical inconsistency through node splitting analysis owing to one comparison of atorvastatin at moderate intensity compared with pravastatin at low intensity (P=0.071); this inconsistency was because one study had a high risk of bias owing to a substantial difference in total cholesterol and HDL-C recordings at baseline^73^ for the measurement of outcome in the pravastatin treatment group (appendix 7). Another inconsistency was found for pravastatin at low intensity compared with simvastatin at moderate intensity (P=0.031). This inconsistency was because one study^44^ showed a moderate risk of bias owing to uncertainty with the randomisation process used and the high number of unexplained discontinuations. Because consistency (transitivity) is a central assumption of a network meta-analysis, we removed both trials, leaving 34 randomised controlled trials for the network on non-HDL-C.

### Performance on non-HDL-C by statin intensity

Figure 2 shows the network meta-analysis results for the primary outcome of all eligible trials after the inconsistency analysis. Rosuvastatin at high (−2.31, 95% credible interval −3.39 to −1.21 mmol/L) and moderate (−2.27, −3.00 to −1.49) intensities, and simvastatin (−2.26, −2.99 to −1.51) and atorvastatin (−2.20, −2.69 to −1.70) at high intensity were the most effective in reducing concentrations of non-HDL-C compared with placebo. Atorvastatin and simvastatin at all intensities and pravastatin at low intensity also significantly reduced levels of non-HDL-C. Although the other statin agents (fluvastatin at low and moderate intensities, pitavastatin at moderate intensity, pravastatin at moderate intensity, and rosuvastatin at low intensity) effectively reduced levels of non-HDL-C compared with placebo, the relative effects were not significant. Heterogeneity was low in the network meta-analysis, with I^2^=0% (95% confidence interval 0 to 38%) (appendix 8).

The surface under the cumulative ranking curve score provides an overall ranking of each treatment. In reducing levels of non-HDL-C, rosuvastatin at moderate intensity was ranked as the best statin treatment (surface under the cumulative ranking curve 77.5%), the second best treatment was rosuvastatin at high intensity (76.8%), the third best was simvastatin at high intensity (76.7%), followed by atorvastatin at high intensity (76.3%). The treatment with the lowest surface under the cumulative ranking curve score (excluding placebo) was fluvastatin at low intensity (8.5%) (appendix 10).

**Fig 3:**
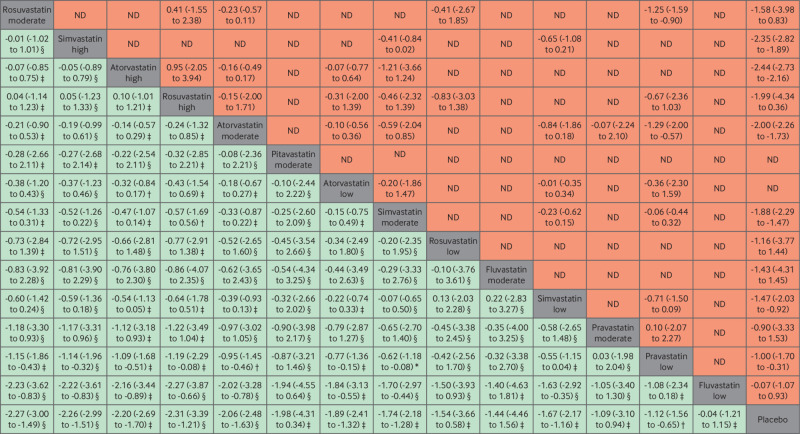
League table of direct comparisons for statin intensities with effect estimates as mean differences (mmol/L). Statin intensities are reported in order of most effective treatment based on surface under the cumulative ranking curve score compared with placebo. Data are standardised mean difference (95% credible interval) in the column defining treatment compared with the row defining treatment. Green=network meta-analysis estimates (105 comparisons); orange=direct pairwise meta-analysis estimates. Appendix 6 gives the numbers of patient and studies. ND=no direct evidence available. The certainty of the evidence, according to the confidence in network meta-analysis (CINeMA) framework, was classified as *very low, †low, ‡moderate, and §high confidence of evidence

Figure 3 is a league table of the results of the network meta-analysis comparing the effects of the different statin intensities. Almost all statins were effective compared with fluvastatin and pravastatin at low intensity. Rosuvastatin at high intensity seemed to be the best performing statin in direct comparisons with other statin intensities, but the difference in effect sizes were not significant and showed a small benefit compared with other top performers (that is, rosuvastatin at moderate intensity, simvastatin at high intensity, and atorvastatin at high intensity). To ensure the certainty of evidence, we incorporated the CINeMA judgments into figure 2 and figure 3. The quality of the evidence according to CINeMA was mostly moderate or high overall (appendix 11), and we found no evidence of funnel plot asymmetry (appendix 12). Results of the sensitivity analyses of the network meta-analysis by specific statin dose were similar to the main statin intensity model, with atorvastatin, rosuvastatin, and simvastatin significantly reducing levels of non-HDL-C (except for rosuvastatin 25 mg) (appendix 13).

### Secondary outcomes

**Fig 4:**
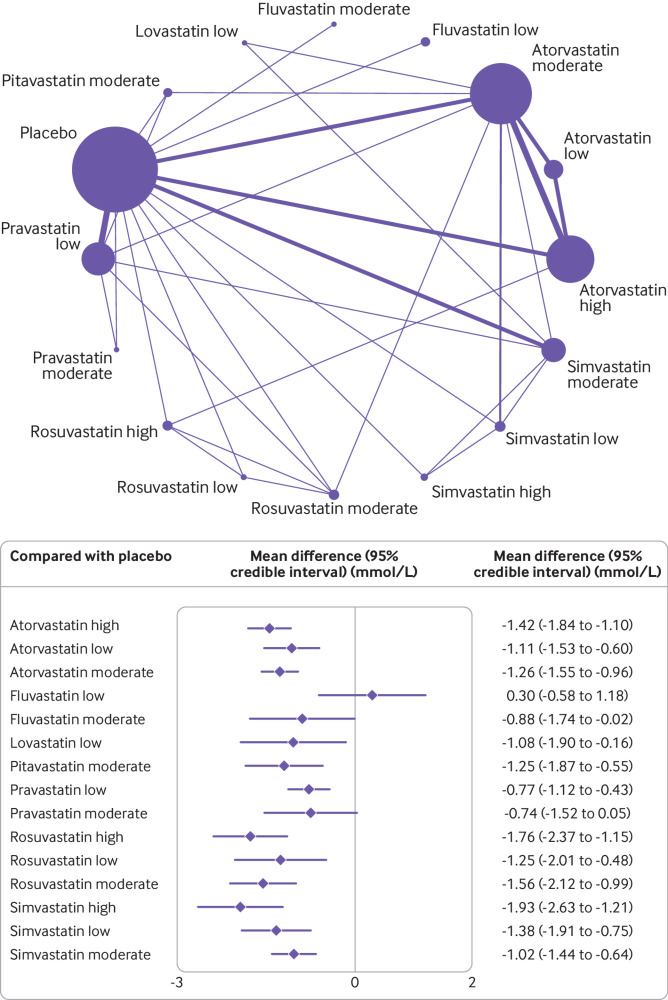
Network of available comparisons between statin intensities for low density lipoprotein cholesterol, and forest plot of network effect sizes of the statin intensities compared with placebo. Size of node is proportional to number of trial participants, and thickness of line connecting nodes is proportional to number of trial participants randomised in trials directly comparing the two treatments

For the secondary outcome, changes in levels of LDL-C, reported in 29 studies (18 two arm, nine three arm, and two four arm), simvastatin (−1.93, 95% credible interval −2.63 to −1.21 mmol/L, surface under the cumulative ranking curve 93%) and rosuvastatin (−1.76, −2.37 to −1.15, 89%) at high intensity doses were the most effective treatment options for reducing levels of LDL-C (fig 4). Heterogeneity was low in this network meta-analysis (I^2^=5%) and no inconsistency was found (appendix 7). For total cholesterol, reported in 36 studies (23 two arm, eight three arm, four four arm, and one five arm), atorvastatin (−2.21, −2.62 to −1.74), rosuvastatin (−2.18, −3.19 to −1.20), and simvastatin (−2.20, −2.96 to −1.42) at high intensity doses were the most effective treatment options for reducing levels of total cholesterol. Of the 12 studies that reported discontinuations of treatment because of an adverse event, only four statin interventions (pravastatin low, atorvastatin moderate, lovastatin low, and simvastatin moderate intensity) were possible for meta-analyses. We found no significant associations in these analysis, although high uncertainty around the estimates was found, as expected. Appendix 9 shows the raw data for discontinuations because of adverse events. Only five studies^6^ ^39^ ^55^ ^59^ ^64^ reported at least one of the three point major cardiovascular event outcomes, for atorvastatin moderate and high intensity treatment groups only. We found a significant reduction in non-fatal myocardial infarction for atorvastatin at moderate intensity compared with placebo (relative risk=0.57, 95% confidence interval 0.43 to 0.76, n=4 studies). We found no significant results for non-fatal stroke or death related to cardiovascular disease. Appendix 9 shows the results for the secondary outcomes.

### Subgroup analysis of patient risk for non-HDL-C

**Fig 5:**
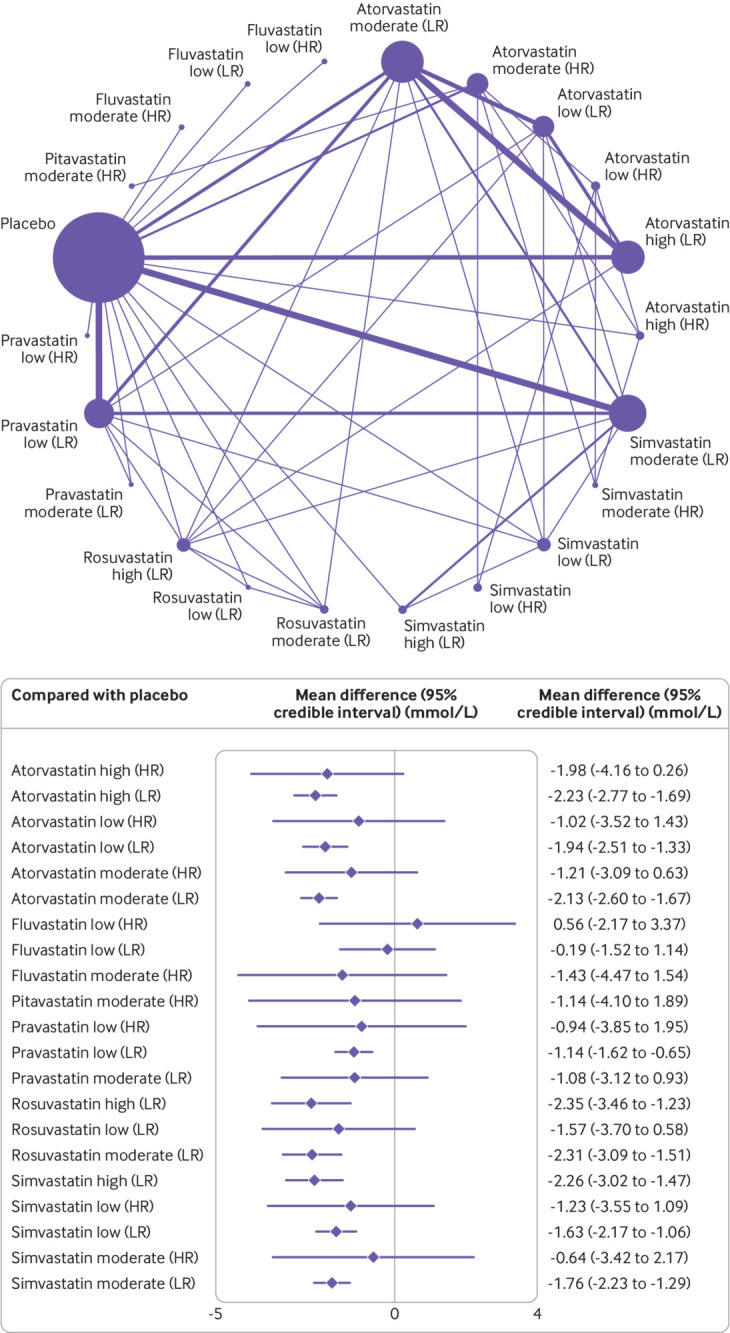
Network of available comparisons between statin intensities for non-high density lipoprotein cholesterol adjusted for patient risk, with forest plot of network effect sizes compared with placebo. Size of node is proportional to number of trial participants, and thickness of line connecting nodes is proportional to number of trial participants randomised in trials directly comparing the two treatments. Patient risk is classified as high (HR) and low to moderate (LR)

Figure 5 shows the subgroup network meta-analysis of 4670 patients with a high risk (10 studies) and 7028 patients with a low-to-medium risk (26 studies) of a major cardiovascular event. The results showed that all statin agents and intensities, except fluvastatin, pravastatin, and rosuvastatin at low intensity, significantly reduced levels of non-HDL-C in patients with a low-to-medium risk of a major cardiovascular event. Atorvastatin at high intensity was the best (not significant, −1.98, 95% credible interval −4.16 to 0.26 mmol/L, surface under the cumulative ranking curve 64%) performer in patients at high risk, and fluvastatin at low intensity (0.56, −2.17 to 3.37, 12%) was the worst. Two studies^50^ ^79^ with a high risk of bias score were removed from the network, but the results did not change (appendix 14).

## Discussion

### Principal findings

This network meta-analysis compared the effectiveness of different statin agents at different intensities in adults with diabetes, with a reduction in levels of non-HDL-C as the primary lipid target. The findings derived from a population of 20 193 participants from randomised clinical trials showed that rosuvastatin, given at moderate and high intensity doses, and simvastatin and atorvastatin at high intensity doses, were the best performing statins at lowering levels of non-HDL-C compared with placebo over an average treatment period of 12 weeks. The network model, adjusting for patient risk, showed that of the 4670 adults (40% of the total number of adults) at high risk of major cardiovascular event outcomes (secondary prevention), atorvastatin at high intensity doses was the most effective. Rosuvastatin, at moderate and high intensity doses, and atorvastatin and simvastatin at high intensity doses, were the most effective statin treatment option in the population of 7028 adults at low to medium risk of major cardiovascular events and possible primary prevention.

### Comparisons with similar research

No previous meta-analysis has assessed the efficacy of statin intensity based on levels of non-HDL-C. But our findings are similar to a recent network meta-analysis^3^ that assessed the primary efficacy endpoint of the same seven statins based on management of levels of LDL-C, HDL-C, and total cholesterol in patients with dyslipidaemia, cardiovascular disease, or diabetes. This meta-analysis concluded that rosuvastatin was the most effect in reducing levels of LDL-C (−72.28 mg/dL, equivalent to −1.87 mmol/L), similar to our estimate of −1.76 mmol/L for rosuvastatin at a high intensity dose. However, the authors highlight that their overall findings should be interpreted with caution because of the large variations in follow-up trial periods, ranging from 14 weeks to 5 years; the intensity and doses of statins involved were not clearly unified; non-HDL-C was not used as an outcome; and several inconsistencies between direct and indirect evidence were identified, which could cause bias in the network findings.

Current evidence suggests that some statins cause more adverse events, and high doses might be more harmful to patients. For instance, a large meta-analysis of 246 955 patients assessing the tolerability and harms of individual statins^81^ found that, compared with other statins, higher doses of atorvastatin and rosuvastatin were associated with a higher risk of discontinuation, and higher doses of atorvastatin, fluvastatin, simvastatin, and pravastatin were associated with a greater risk of increases in levels of transaminase. Our meta-analysis on discontinuations because of adverse events involved fewer studies and patients, and few events were reported; some studies even reported no events in both treatment arms. Meta-analysing rare events is problematic in this setting and can give spurious findings.^82^ ^83^ ^84^ Therefore, the results on discontinuations need to be interpreted with caution. Discontinuations because of adverse events and other harm outcomes should be reported in a CONSORT (CONsolidated Standards of Reporting Trials) flow diagram and in the study results.^85^ The primary reports for drug treatment and device trials for chronic heart failure,^86^ and more specifically for statins,^87^ often do not provide these data, however. Practising cardiologists need these data to help support clinical judgments when balancing the benefit and harms of different statins.

### Implications for policy and practice

The use of lipid or apolipoprotein parameters other than LDL-C as targets for treatment with statins continues to be strongly debated. The clinical applicability of non-HDL-C and LDL-C are identical, however, with the garnered evidence suggesting that non-HDL-C might be superior to LDL-C as a marker of cardiovascular risk,^9^ ^88^ ^89^ ^90^ and therefore non-HDL-C levels are likely to be a more appropriate target than LDL-C levels for treatment with statins in the future.^9^ With non-HDL-C as a potential key lipid target, NICE has now recommended that physicians use non-HDL-C as their primary target. Specifically, NICE has set a target of reducing levels of non-HDL-C by >40% from baseline when high intensity statins are used. If the baseline value is not available, however, the Joint British Societies consensus recommends a target level of <2.5 mmol/L for non-HDL-C (equivalent to LDL-C <1.8 mmol/L).^91^ Further evidence from observational primary care data suggests that at the time of diagnosis of diabetes, the mean concentration of non-HDL-C is 4 mmol/L.^92^ Many of the patients in their study were taking statins and they would be expected to reach the target of <2.5 mmol/L over time.

### Strengths and limitations of the study

Our review assessed the efficacy of the most prescribed statin treatments at different intensities in reducing levels of non-HDL-C in patients with diabetes. Our analysis used robust methods, including bayesian meta-analysis and rigorous quality assessment by CINeMA.

Our study had several limitations. Classification of patient risk was not confirmed at the individual participant level and hence cannot be considered exact. Therefore, we made assumptions based on the study inclusion and exclusion criteria and baseline characteristics to determine an appropriate risk category according to definitions of major cardiovascular events. Also, a flexible method for estimating the effective sample size in an indirect comparison meta-analysis and network meta-analysis^93^ suggests that some of the pairwise comparisons in the high risk group were underpowered, meaning the results of the subgroup analysis should be interpreted with caution. Our sensitivity analysis, however, removing the studies with a high risk of bias, showed no major differences from the main results.

Only two studies^63^ ^74^ assessed people with type 1 diabetes, thus limiting our results to mostly patients with type 2 diabetes. The primary focus of our review and of the included studies was on surrogate outcome (lipid) measures and not cardiovascular disease or major cardiovascular event outcomes. Thus our results should mainly act as guidance for whether individuals will reach the target levels of non-HDL-C, LDL-C, and total cholesterol with a specific statin treatment delivered at a certain intensity. Nevertheless, the three point major cardiovascular event outcomes were analysed, but because only five studies^6^ ^55^ ^59^ ^64^ ^67^ reported these outcomes, the results were limited.

This study was a (network) meta-analysis of aggregate data, and standard practice when dealing with aggregate data is to focus on the final score values to obtain an effect estimate at the study level. Thus, based on this principle, our effect estimates did not adjust for baseline differences. A major constraint underlying this type of adjustment is that baseline data are often not reported in studies, which was the case in the studies in this meta-analysis. Also, if we had included studies that provided baseline data, a different methodological approach would be required, which would have substantially reduced the number of trials in our study pool.^94^ Nevertheless, meta-analysing randomised controlled trials makes this method feasible and defensible, because the underlying assumption (at least in large randomised controlled trials) is that baseline levels are almost identical in the two (or more) treatment arms, which was also shown to be true in the transitivity assessment as part of our CINeMA evaluation. With meta-analyses of aggregate data, little else can be done about baseline scores, except perhaps a meta-regression where the association between baseline levels and effect sizes can be explored. This approach comes with considerable power limitations, however, even for large meta-analyses. Hence to investigate the role of baseline scores, an individual patient data meta-analysis would be required. We tried contacting the authors for their baseline data, but only three responded.^38^ ^73^ ^80^ We recommend that these data are reported in future trials to allow the adjusted baseline change scores to be used instead of the final scores.

### Conclusions

Rosuvastatin, given at moderate intensity doses, and rosuvastatin, simvastatin, and atorvastatin, given at high intensity doses, were the most effective treatments in patients with diabetes, modestly reducing levels of non-HDL-C over 12 weeks compared with placebo. Given the potential improvement in accuracy in predicting cardiovascular disease when non-HDL-C is used as the primary target, our findings could inform policy on which statin types and intensities are most effective by reducing non-HDL-C in patients with diabetes and at risk of cardiovascular disease.

**What is already known on this topic**

In people with diabetes, statins are the basis of primary and secondary prevention of cardiovascular disease by reducing plasma levels of low density lipoprotein cholesterol (LDL-C), but evidence is lacking on the comparative effectiveness of statins on non-high density lipoprotein cholesterol (non-HDL-C)
Non-HDL-C is thought to be more strongly associated with the risk of cardiovascular disease than LDL-C in statin users, and therefore might be a better tool for assessing the risk of cardiovascular disease and the effects of treatment
Guidelines from the National Institute for Health and Care Excellence for adults with diabetes recommend that non-HDL-C should replace LDL-C as the primary target for reducing the risk of cardiovascular disease when taking lipid lowering agents

**What this study adds**

Rosuvastatin, given at moderate and high intensity doses, and simvastatin and atorvastatin, given at high intensity doses, were the most effective treatments in patients with diabetes, reducing concentrations of non-HDL-C by 2.20-2.31 mmol/L over 12 weeks
In patients at high risk of major cardiovascular events (secondary prevention), atorvastatin at high intensity doses showed the largest reduction in non-HDL-C (~2.0 mmol/L)
These findings can guide decision making for clinicians and support policy guidelines for the management of lipid levels, with non-HDL-C as a primary target, in patients with diabetes

## Data Availability

AH had full access to all the data in the study and takes responsibility for the integrity of the data and the accuracy of the data analysis. All data are publicly available.

## Web extra

Extra material supplied by authors

Web appendix: Supplementary appendix

## Notes

Contributors: AH, MP, and EK had the initial research idea and obtained funding for this study. AH, MP, and DT formulated the research questions and designed the study. AH and DT searched for published work, selected articles, and extracted data. AH performed all analyses and EK checked over. AH and DT drafted the protocol and the manuscript. MP helped in the searching of articles and data selection and extraction. MP contributed to designing the searches and the statistical analysis plan, writing of the manuscript, and interpreting the findings. MAM, MKR, and EK contributed to the manuscript by providing review comments and edits. AH is the study guarantor. The corresponding author attests that all listed authors meet authorship criteria and that no others meeting the criteria have been omitted.

The lead author AH affirms that the manuscript is an honest, accurate, and transparent account of the study being reported; that no important aspects of the study have been omitted; and that any discrepancies from the study as planned (and, if relevant, registered) have been explained.

Dissemination to participants and related patient and public communities: Dissemination of this research will be done at the World Heart Congress in October 2022 (https://heart.euroscicon.com/), and through a press release from the University of Manchester, social media and twitter, and charities, including the British Heart Foundation and Heart Research Institute UK.

Provenance and peer review: Not commissioned; externally peer reviewed.
