# Supplementary material for: Comparative effectiveness of statins on non-high density lipoprotein cholesterol in people with diabetes and at risk of cardiovascular disease: systematic review and network meta-analysis
Source: BMJ. 2022 Mar 24;376:e067731. doi: 10.1136/bmj-2021-067731 (PMC8943592; doi:10.1136/bmj-2021-067731)
Supplement: Supplementary file 1 — Web appendix: Supplementary appendix [file hoda067731.ww.pdf]

Supplementary Appendix

Contents

Appendix 1: PRISMA Extension Checklist 3

Appendix 2: Search Criteria 7

Appendix 3: Citations of Included Studies 10

Appendix 4: Characteristics of Included Studies 13

Appendix 5: Risk of Bias Assessment 29

Appendix 6: Deviance statistics of convergence model, Mean Posterior values by Dose and Meta-Analysis of Direct (Pairwise) Evidence 33

Appendix 7: Inconsistency Analysis 38

Appendix 8: Heterogeneity Assessment 50

Appendix 9: Secondary outcomes (TC, Discontinuations and MACE) 55

Appendix 10: Treatment Ranking using SUCRA 63

Appendix 11: CINeMA Assessment 68

Appendix 12: Comparison-Adjusted Funnel Plots 79

Appendix 13: Sensitivity Analysis of Network Model by Dose 81

Appendix 14: Further Results for the Subgroup Analysis 92

Appendix 1: PRISMA Extension Checklist

PRISMA NMA Checklist of Items to Include When Reporting A Systematic Review Involving a Network Meta-analysis

Section/Topic	Item #	Checklist Item	Reported on Page #
**TITLE**			
Title	1	Identify the report as a systematic review *incorporating a network meta-analysis (or related form of meta-analysis).*	1
**ABSTRACT**			
Structured summary	2	Provide a structured summary including, as applicable:<br>**Background:** main objectives<br>**Methods:** data sources; study eligibility criteria, participants, and interventions; study appraisal; and *synthesis methods, such as network meta-analysis.*<br>**Results:** number of studies and participants identified; summary estimates with corresponding confidence/credible intervals; *treatment rankings may also be discussed. Authors may choose to summarize pairwise comparisons against a chosen treatment included in their analyses for brevity.*<br>**Discussion/Conclusions:** limitations; conclusions and implications of findings.<br>**Other:** primary source of funding; systematic review registration number with registry name.	2
**INTRODUCTION**			
Rationale	3	Describe the rationale for the review in the context of what is already known, *including mention of why a network meta-analysis has been conducted.*	3
Objectives	4	Provide an explicit statement of questions being addressed, with reference to participants, interventions, comparisons, outcomes, and study design (PICOS).	4
**METHODS**			
Protocol and registration	5	Indicate whether a review protocol exists and if and where it can be accessed (e.g., Web address); and, if available, provide registration information, including registration number.	4
Eligibility criteria	6	Specify study characteristics (e.g., PICOS, length of follow-up) and report characteristics (e.g., years considered, language, publication status) used as criteria for eligibility, giving rationale. *Clearly describe eligible treatments included in the treatment network and note whether any have been clustered or merged into the same node (with justification).*	4-5
Information sources	7	Describe all information sources (e.g., databases with dates of coverage, contact with study authors to identify additional studies) in the search and date last searched.	4
Search	8	Present full electronic search strategy for at least one	4

		database, including any limits used, such that it could be repeated.	
Study selection	9	State the process for selecting studies (i.e., screening, eligibility, included in systematic review, and, if applicable, included in the meta-analysis).	4
Data collection process	10	Describe method of data extraction from reports (e.g., piloted forms, independently, in duplicate) and any processes for obtaining and confirming data from investigators.	5
Data items	11	List and define all variables for which data were sought (e.g., PICOS, funding sources) and any assumptions and simplifications made.	5
**Geometry of the network**	**S1**	Describe methods used to explore the geometry of the treatment network under study and potential biases related to it. This should include how the evidence base has been graphically summarized for presentation, and what characteristics were compiled and used to describe the evidence base to readers.	5-6
Risk of bias within individual studies	12	Describe methods used for assessing risk of bias of individual studies (including specification of whether this was done at the study or outcome level), and how this information is to be used in any data synthesis.	5-6
Summary measures	13	State the principal summary measures (e.g., risk ratio, difference in means). *Also describe the use of additional summary measures assessed, such as treatment rankings and surface under the cumulative ranking curve (SUCRA) values, as well as modified approaches used to present summary findings from meta-analyses.*	6
Planned methods of analysis	14	Describe the methods of handling data and combining results of studies for each network meta-analysis. This should include, but not be limited to:<br>• *Handling of multi-arm trials.*<br>• *Selection of variance structure.*<br>• *Selection of prior distributions in Bayesian analyses; and*<br>• *Assessment of model fit.*	6-7
**Assessment of Inconsistency**	**S2**	Describe the statistical methods used to evaluate the agreement of direct and indirect evidence in the treatment network(s) studied. Describe efforts taken to address its presence when found.	6
Risk of bias across studies	15	Specify any assessment of risk of bias that may affect the cumulative evidence (e.g., publication bias, selective reporting within studies).	5-6
Additional analyses	16	Describe methods of additional analyses if done, indicating which were pre-specified. This may include, but not be limited to, the following:<br>• Sensitivity or subgroup analyses.<br>• Meta-regression analyses.<br>• *Alternative formulations of the treatment network; and*<br>• *Use of alternative prior distributions for Bayesian analyses (if applicable).*	6-7

**RESULTS†**			
Study selection	17	Give numbers of studies screened, assessed for eligibility, and included in the review, with reasons for exclusions at each stage, ideally with a flow diagram.	7
**Presentation of network structure**	**S3**	Provide a network graph of the included studies to enable visualization of the geometry of the treatment network.	8
**Summary of network geometry**	**S4**	Provide a brief overview of characteristics of the treatment network. This may include commentary on the abundance of trials and randomized patients for the different interventions and pairwise comparisons in the network, gaps of evidence in the treatment network, and potential biases reflected by the network structure.	8
Study characteristics	18	For each study, present characteristics for which data were extracted (e.g., study size, PICOS, follow-up period) and provide the citations.	7-8
Risk of bias within studies	19	Present data on risk of bias of each study and, if available, any outcome level assessment.	8
Results of individual studies	20	For all outcomes considered (benefits or harms), present, for each study: 1) simple summary data for each intervention group, and 2) effect estimates and confidence intervals. *Modified approaches may be needed to deal with information from larger networks.*	8
Synthesis of results	21	Present results of each meta-analysis done, including confidence/credible intervals. *In larger networks, authors may focus on comparisons versus a particular comparator (e.g., placebo or standard care), with full findings presented in an appendix. League tables and forest plots may be considered to summarize pairwise comparisons.* If additional summary measures were explored (such as treatment rankings), these should also be presented.	9-10
**Exploration for inconsistency**	**S5**	Describe results from investigations of inconsistency. This may include such information as measures of model fit to compare consistency and inconsistency models, *P* values from statistical tests, or summary of inconsistency estimates from different parts of the treatment network.	8
Risk of bias across studies	22	Present results of any assessment of risk of bias across studies for the evidence base being studied.	9
Results of additional analyses	23	Give results of additional analyses, if done (e.g., sensitivity or subgroup analyses, meta-regression analyses, *alternative network geometries studied, alternative choice of prior distributions for Bayesian analyses,* and so forth).	9
**DISCUSSION**			
Summary of evidence	24	Summarize the main findings, including the strength of evidence for each main outcome; consider their relevance to key groups (e.g., healthcare providers, users, and policymakers).	10

Limitations	25	Discuss limitations at study and outcome level (e.g., risk of bias), and at review level (e.g., incomplete retrieval of identified research, reporting bias). *Comment on the validity of the assumptions, such as transitivity and consistency. Comment on any concerns regarding network geometry (e.g., avoidance of certain comparisons).*	11-12
Conclusions	26	Provide a general interpretation of the results in the context of other evidence, and implications for future research.	13
**FUNDING**			
Funding	27	Describe sources of funding for the systematic review and other support (e.g., supply of data), role of funders for the systematic review. This should also include information regarding whether funding has been received from manufacturers of treatments in the network and/or whether some of the authors are content experts with professional conflicts of interest that could affect use of treatments in the network.	14

PICOS = population, intervention, comparators, outcomes, study design.
* Text in italics indicateS wording specific to reporting of network meta-analyses that has been added to guidance from the PRISMA statement.
† Authors may wish to plan for use of appendices to present all relevant information in full detail for items in this section.

Appendix 2: Search Criteria

Ovid Medline (1946 to December 2021)

#	Terms	Number of Citations
1	exp Hydroxymethylglutaryl-CoA Reductase Inhibitors/	42548
2	(atorvastatin or fluvastatin or lovastatin or pitavastatin or pravastatin or rosuvastatin or simvastatin).mp.	27663
3	(lipitor or lescol or mevacor or livalo or pravachol or crestor or zocor).mp.	483
4	2 or 3	27723
5	1 or 4	46309
6	Diabetes Mellitus, Type 1/ or Diabetes Mellitus, Type 2/ or diabetes.mp. or Diabetes Mellitus/	582109
7	Cholesterol, LDL/	28703
8	(LDL cholesterol or LDL-c).mp.	36486
9	7 or 8	50434
10	5 and 6 and 9	1620
11	limit 10 to (clinical trial, all or clinical trial or comparative study or controlled clinical trial or randomized controlled trial)	579
12	randomized controlled trial.pt.	536101
13	controlled clinical trial.pt.	94244
14	randomized.mp. or randomised.tw. [mp=title, abstract, original title, name of substance word, subject heading word, floating sub-heading word, keyword heading word, organism supplementary concept word, protocol supplementary concept word, rare disease supplementary concept word, unique identifier, synonyms]	852433
15	placebo.tw.	203787
16	randomly.ab.	305428
17	trial.tw.	559657
18	or/12-17	1360407
19	10 and 18	736
**20**	**11 or 19**	**859**

Cochrane Central Register of Controlled Trials (December 2021)

#	Terms	Number of Citations
1	exp Hydroxymethylglutaryl-CoA Reductase Inhibitors/	5452
2	(atorvastatin or fluvastatin or lovastatin or pitavastatin or pravastatin or rosuvastatin or simvastatin).mp.	13170
3	(lipitor or lescol or mevacor or livalo or pravachol or crestor or zocor).mp.	442
4	2 or 3	13209
5	1 or 4	13996
6	Diabetes Mellitus, Type 1/ or Diabetes Mellitus, Type 2/ or diabetes.mp. or Diabetes Mellitus/	90730
7	Cholesterol, LDL/	4817
8	(LDL cholesterol or LDL-c).mp.	14605
9	7 or 8	16445
**10**	**5 and 6 and 9**	**846**

Embase (1974 to December 2021)

#	Terms	Number of Citations
1	exp Hydroxymethylglutaryl-CoA Reductase Inhibitors/	165145
2	(atorvastatin or fluvastatin or lovastatin or pitavastatin or pravastatin or rosuvastatin or simvastatin).mp.	85264
3	(lipitor or lescol or mevacor or livalo or pravachol or crestor or zocor).mp.	4993
4	2 or 3	85546
5	1 or 4	166665
6	Diabetes Mellitus, Type 1/ or Diabetes Mellitus, Type 2/ or diabetes.mp. or Diabetes Mellitus/	1131277
7	Cholesterol, LDL/	97478
8	(LDL cholesterol or LDL-c).mp.	65092
9	7 or 8	119985
10	5 and 6 and 9	8401
11	(random* or factorial* or placebo* or assign* or allocat* or crossover*).tw.	2168848
12	(trial* and (control* or comparative)).tw.	691381
13	((blind* or mask*) and (single or double or triple or treble)).tw.	285963
14	(treatment adj arm*).tw.	22367
15	(control* adj group*).tw.	716627
16	rct.tw.	42525
17	DOUBLE BLIND PROCEDURE/	185363
18	SINGLE BLIND PROCEDURE/	43054
19	RANDOMIZATION/	91106
20	PLACEBO/	368040
21	exp Clinical Trial/	1606640
22	or/11-21	3799216
**23**	**10 and 22**	**1579**

Appendix 3: Citations of Included Studies

Citations for the 42 eligible studies:

1. Visseren, F. , Bouter, P. , van Loon, B. , Erkelens, W. & (2001). Treatment of Dyslipidaemia with Fluvastatin in Patients with Type 2 Diabetes Mellitus. Clinical Drug Investigation, 21 (10), 671-678.
2. The effect of aggressive versus standard lipid lowering by atorvastatin on diabetic dyslipidemia: the DALI study: a double-blind, randomized, placebo-controlled trial in patients with type 2 diabetes and diabetic dyslipidemia. *Diabetes Care* 2001; **24**(8): 1335-41.
3. Behounek BD, Mcgovern ME, Kassler-Taub KB, Markowitz SJ, Bergman M, DIABETES TPMSGF. A multinational study of the effects of low-dose pravastatin in patients with non-insulin-dependent diabetes mellitus and hypercholesterolemia. *Clinical Cardiology* 1994; **17**(10): 558-62.
4. Berberoglu Z, Guvener N, Cangoz B, Asik M, Yazici AC, Karatas M. Effects of Achieving an LDL-Cholesterol Level of <70 mg/dL Compared With the Goal of <100 mg/dL Using Simvastatin or Atorvastatin on Cognitive Processes in High-Risk Diabetic Patients. *The Endocrinologist* 2009; **19**(6): 271-9.
5. Betteridge DJ, Gibson JM, Sager PT. Comparison of Effectiveness of Rosuvastatin Versus Atorvastatin on the Achievement of Combined C-Reactive Protein (<2 mg/L) and Low-Density Lipoprotein Cholesterol (<70 mg/dl) Targets in Patients With Type 2 Diabetes Mellitus (from the ANDROMEDA Study). *American Journal of Cardiology* 2007; **100**(8): 1245-8.
6. Chang YH, Lin KC, Chang DM, Hsieh CH, Lee YJ. Paradoxical negative HDL cholesterol response to atorvastatin and simvastatin treatment in Chinese type 2 diabetic patients. *Rev Diabet Stud* 2013; **10**(2-3): 213-22.
7. Cheung RC, Morrell JM, Kallend D, Watkins C, Schuster H. Effects of switching statins on lipid and apolipoprotein ratios in the MERCURY I study. *Int J Cardiol* 2005; **100**(2): 309-16.
8. Chu CH, Lee JK, Lam HC, et al. Atorvastatin does not affect insulin sensitivity and the adiponectin or leptin levels in hyperlipidemic Type 2 diabetes. *J Endocrinol Invest* 2008; **31**(1): 42-7.
9. Colhoun HM, Betteridge DJ, Durrington PN, et al. Primary prevention of cardiovascular disease with atorvastatin in type 2 diabetes in the Collaborative Atorvastatin Diabetes Study (CARDS): multicentre randomised placebo-controlled trial. *Lancet* 2004; **364**(9435): 685-96.
10. Dalla Nora E, Passaro A, Zamboni PF, Calzoni F, Fellin R, Solini A. Atorvastatin improves metabolic control and endothelial function in type 2 diabetic patients: a placebo-controlled study. *J Endocrinol Invest* 2003; **26**(1): 73-8.
11. Dallinga-Thie GM, van Tol A, Dullaart RP. Plasma pre beta-HDL formation is decreased by atorvastatin treatment in type 2 diabetes mellitus: Role of phospholipid transfer protein. *Biochim Biophys Acta* 2009; **1791**(8): 714-8.
12. Ferrer-García JC, Sanchez-Ballester E, Albalat-Galera R, Berzosa-Sanchez M, Herrera-Ballester A. Efficacy of atorvastatin for achieving cholesterol targets after LDL-cholesterol based dose selection in patients with type 2 diabetes. *J Cardiovasc Pharmacol Ther* 2008; **13**(3): 183-8.
13. Gentile S, Turco S, Guarino G, et al. Comparative efficacy study of atorvastatin vs. simvastatin, pravastatin, lovastatin and placebo in type 2 diabetic patients with hypercholesterolaemia. *Diabetes, Obesity and Metabolism* 2000; **2**(6): 355-62.
14. Goldberg RB, Guyton JR, Mazzone T, et al. Ezetimibe/simvastatin vs atorvastatin in patients with type 2 diabetes mellitus and hypercholesterolemia: the VYTAL study. *Mayo Clin Proc* 2006; **81**(12): 1579-88.
15. Hadjibabaie M, Gholami K, Khalili H, et al. Comparative efficacy and safety of atorvastatin, simvastatin and lovastatin in the management of dyslipidemic Type 2 diabetic patients. *Therapy* 2006; **3**: 759-64.
16. Ichihara A, Hayashi M, Ryuzaki M, Handa M, Furukawa T, Saruta T. Fluvastatin prevents development of arterial stiffness in haemodialysis patients with type 2 diabetes mellitus. *Nephrol Dial Transplant* 2002; **17**(8): 1513-7.

17. Insull Jr W, Kafonek S, Goldner D, Zieve F. Comparison of efficacy and safety of atorvastatin (10mg) with simvastatin (10mg) at six weeks. ASSET Investigators. *The American journal of cardiology* 2001; **87**: 554-9.
18. Ishigaki Y, Kono S, Katagiri H, Oka Y, Oikawa S. Elevation of HDL-C in response to statin treatment is involved in the regression of carotid atherosclerosis. *J Atheroscler Thromb* 2014; **21**(10): 1055-65.
19. Janatuinen T, Knuuti J, Toikka JO, et al. Effect of pravastatin on low-density lipoprotein oxidation and myocardial perfusion in young adults with type 1 diabetes. *Arterioscler Thromb Vasc Biol* 2004; **24**(7): 1303-8.
20. Jialal I, Devaraj S. Statin therapy in acute cardiovascular syndromes. *Curr Opin Lipidol* 2007; **18**(5): 610-2.
21. Kim JH, Lee MR, Shin JA, et al. Effects of pravastatin on serum adiponectin levels in female patients with type 2 diabetes mellitus. *Atherosclerosis* 2013; **227**(2): 355-9.
22. Kim JM, Back MK, Yi HS, Joung KH, Kim HJ, Ku BJ. Effect of Atorvastatin on Growth Differentiation Factor-15 in Patients with Type 2 Diabetes Mellitus and Dyslipidemia. *Diabetes Metab J* 2016; **40**(1): 70-8.
23. Knopp RH, d'Emden M, Smilde JG, Pocock SJ. Efficacy and safety of atorvastatin in the prevention of cardiovascular end points in subjects with type 2 diabetes: the Atorvastatin Study for Prevention of Coronary Heart Disease Endpoints in non-insulin-dependent diabetes mellitus (ASPEN). *Diabetes Care* 2006; **29**(7): 1478-85.
24. Koh KK, Oh PC, Sakuma I, Lee Y, Han SH, Shin EK. Rosuvastatin dose-dependently improves flow-mediated dilation, but reduces adiponectin levels and insulin sensitivity in hypercholesterolemic patients. *Int J Cardiol* 2016; **223**: 488-93.
25. Koh KK, Quon MJ, Han SH, et al. Simvastatin improves flow-mediated dilation but reduces adiponectin levels and insulin sensitivity in hypercholesterolemic patients. *Diabetes Care* 2008; **31**(4): 776-82.
26. Koh KK, Quon MJ, Han SH, et al. Differential metabolic effects of pravastatin and simvastatin in hypercholesterolemic patients. *Atherosclerosis* 2009; **204**(2): 483-90.
27. Lawrence JM, Reid J, Taylor GJ, Stirling C, Reckless JP. The effect of high dose atorvastatin therapy on lipids and lipoprotein subfractions in overweight patients with type 2 diabetes. *Atherosclerosis* 2004; **174**(1): 141-9.
28. Lewin AJ, Kipnes MS, Meneghini LF, et al. Effects of simvastatin on the lipid profile and attainment of low-density lipoprotein cholesterol goals when added to thiazolidinedione therapy in patients with type 2 diabetes mellitus: A multicenter, randomized, double-blind, placebo-controlled trial. *Clin Ther* 2004; **26**(3): 379-89.
29. Liu PY, Lin LY, Lin HJ, et al. Pitavastatin and Atorvastatin double-blind randomized comPArative study among hiGh-risk patients, including thOse with Type 2 diabetes mellitus, in Taiwan (PAPAGO-T Study). *PLoS One* 2013; **8**(10): e76298.
30. Miller M, Dobs A, Yuan Z, Battisti WP, Borisute H, Palmisano J. Effectiveness of simvastatin therapy in raising HDL-C in patients with type 2 diabetes and low HDL-C. *Curr Med Res Opin* 2004; **20**(7): 1087-94.
31. Mori H, Okada Y, Tanaka Y. Effects of pravastatin, atorvastatin, and rosuvastatin in patients with type 2 diabetes mellitus and hypercholesterolemia. *Diabetology International* 2013; **4**(2): 117-25.
32. Paolisso G, Sgambato S, De Riu S, et al. Simvastatin reduces plasma lipid levels and improves insulin action in elderly, non-insulin dependent diabetics. *Eur J Clin Pharmacol* 1991; **40**(1): 27-31.
33. Schneider JG, von Eynatten M, Parhofer KG, et al. Atorvastatin improves diabetic dyslipidemia and increases lipoprotein lipase activity in vivo. *Atherosclerosis* 2004; **175**(2): 325-31.
34. Sever PS, Poulter NR, Dahlöf B, et al. Reduction in cardiovascular events with atorvastatin in 2,532 patients with type 2 diabetes: Anglo-Scandinavian Cardiac Outcomes Trial--lipid-lowering arm (ASCOT-LLA). *Diabetes Care* 2005; **28**(5): 1151-7.

35. Sindhu S, Singh HK, Salman MT, Fatima J, Verma VK. Effects of atorvastatin and rosuvastatin on high-sensitivity C-reactive protein and lipid profile in obese type 2 diabetes mellitus patients. *J Pharmacol Pharmacother* 2011; **2**(4): 261-5.
36. Son JW, Kim DJ, Lee CB, et al. Effects of patient-tailored atorvastatin therapy on ameliorating the levels of atherogenic lipids and inflammation beyond lowering low-density lipoprotein cholesterol in patients with type 2 diabetes. *J Diabetes Investig* 2013; **4**(5): 466-74.
37. Tekin A, Tekin G, Sezgin AT, Müderrisoğlu H. Short- and long-term effect of simvastatin therapy on the heterogeneity of cardiac repolarization in diabetic patients. *Pharmacol Res* 2008; **57**(5): 393-7.
38. Thongtang N, Piyapromdee J, Tangkittikasem N, Samaithongcharoen K, Srikanchanawat N, Sriussadaporn S. Efficacy and Safety of Switching from Low-Dose Statin to High-Intensity Statin for Primary Prevention in Type 2 Diabetes: A Randomized Controlled Trial. *Diabetes Metab Syndr Obes* 2020; **13**: 423-31.
39. Winkler K, Abletshauser C, Hoffmann MM, et al. Effect of fluvastatin slow-release on low density lipoprotein (LDL) subfractions in patients with type 2 diabetes mellitus: baseline LDL profile determines specific mode of action. *J Clin Endocrinol Metab* 2002; **87**(12): 5485-90.
40. Wolffenbuttel BH, Franken AA, Vincent HH. Cholesterol-lowering effects of rosuvastatin compared with atorvastatin in patients with type 2 diabetes -- CORALL study. *J Intern Med* 2005; **257**(6): 531-9.
41. Xu K, Han YL, Jing QM, et al. Lipid-modifying therapy in diabetic patients with high plasma non-high-density lipoprotein cholesterol after percutaneous coronary intervention. *Exp Clin Cardiol* 2007; **12**(1): 48-50.
42. Zhang A, Vertommen J, Van Gaal L, De Leeuw I. Effects of pravastatin on lipid levels, in vitro oxidizability of non-HDL lipoproteins and microalbuminuria in IDDM patients. *Diabetes Res Clin Pract* 1995; **29**(3): 189-94.

Appendix 4: Characteristics of Included Studies

Study, Author & Date (Country)	Statin Agent (dose mg's) *[Intensity]	Placebo-Controlled	Assessment times (By dose)	No. of patients	Mean age, SD (median, range/IQR)	¥% Male	€% White ethnicity	Outcome (s)	BMI (kg/m2) at baseline	Diabetes type	Years of diagnosis	Comorbidity	Concomitant medication (other lipids lowering treatment)	ᶲPatient Risk (Reason for judgement)
Behounek 1994 (US)	Pravastatin (10mg) [L]	Yes	16 weeks	325	Mean: 58.3 (Range: 37-70) (Int)	50.3% (Int)	NR	TC; LDL-c; HDL-c	Mean weight: 74.9 (Range: 144-196) kg	Type I or II	1	Non-insulin-dependent diabetes mellitus and hypercholesterolemia	NR	**Low** (excl. previous cardiovascular disease patient)
Berberoglu 2009 (Turkey)	Atorvastatin (10mg) [M]<br><br>Simvastatin (10mg) [L]	No	12 weeks	140	S: LDL<70 mg/dL: 60.8 (SD 7.1); LDL<100 mg/d: 61.3 (8)<br><br>A: LDL<70 mg/dL: 60 (7.8); LDL<100 mg/d: 62.2 (7.5)	S: LDL<70 mg/dL: 9M/19F); LDL<100 mg/d: 15M/14F<br><br>A: LDL<70 mg/dL: 10M/20F; LDL<100 mg/d: 9M/18F	NR	TC; LDL-c; HDL-c	NR	Type I or II	S: LDL<70 mg/dL: 4.4 (7.5) yrs.; LDL<100 mg/d: 4.2 (4.8) yrs.<br><br>A: LDL<70 mg/dL: 4.5 (7.7) yrs.; LDL<100 mg/d: 4.1 (5.3)	Patients had overt CVD, or one or more major CV risk factors at recruitment	All patient were free from any lipid lowering therapy for at least 6-weeks prior to recruitment	**High** (Patient has overt CVD, or one major CV risk factors inc. hypertension, cigarette smoking, family history of CAD.
Betteridge & Gibson 2007 (UK)	Atorvastatin (10mg, 20mg) [M]<br><br>Rosuvastatin (10mg, 20mg) [H]	No	8 weeks (10mg) 16 weeks (20mg)	509	Total: 61.5 (SD 10.7)	61.10%	NR	TC; LDL-c; HDL-c	NR	Type II	NR	NR	NR	**Low** (excl. a prior history of cardiovascular disease or familial hypercholesterolaemia; resting diastolic or systolic blood pressure of > 95 mmHg or > 200 mmHg)
Chang 2013 (Taiwan)	Atorvastatin (10mg [M], 20mg [M], 40mg [H])	No	12 weeks (A: 10mg, 20mg, 40mg) 48 weeks	1,080	A: 60.9 (10.9); S: 61.1 (10.9)	A: 456F/298M; S: 185F/141M	100% Asian (Taiwanese)	TC; LDL-c; HDL-c	A: 25.5 (SD 3.5); S: 25.5 (3.9)	Type II	A: 9.7 (SD 6.6) yrs.; S: 9.7 (6.2) yrs.	Hypertension diagnosis at recruitment	Already prescribed anti-hypertensive medication at	**High** (Over 56% has hypertension diagnosis at baseline)

	Simvastatin (20mg) [M]		(A: 10mg; S: 20mg)										time of enrolment. Patients who had been prescribed 10mg atorvastatin and 20mg simvastatin for at least 12 months were included in the study	
Cheung 2005 (EU, Canada, and Australia)	Atorvastatin (10mg, 20mg) [M] Pravastatin (40mg) [L] Rosuvastatin (10mg) [H] Simvastatin (20mg) [M]	No	8 weeks	3140	62 and 63 yrs.	55% to 60% male	99% White (European/western)	Non-HDL-c, TC, LDL-c, HDL-c	28.2 to 28.9	Type II	NR	Patients with CAD or other atherosclerotic disease or T2D were enrolled	6-week dietary lead-in period, during which all lipid lowering treatments were discontinued	**Medium** (20% 10-year risk of CAD)
Chu 2008 (Taiwan)	Atorvastatin (10mg [M], 20mg [M], 40mg [H])	No	12 weeks	29	10mg: 59.3 (12.8); 20mg: 58.5 (13); 40mg: 62.6 (9.6)	10mg: 7F/3M; 20mg: 6F/4M; 40mg: 1F/8M	100% Asian (Taiwanese)	TC; LDL-c; HDL-c	10mg: 25.1 (1); 20mg: 25.9 (0.6); 40mg: 23.1 (1.1)	Type II	NR	Hyperlipidaemia and Type 2 diabetics were recruited	Patients were using oral anti-diabetic drugs prior to recruitment	**Medium** (10mg: 3/10 hypertension; 20mg: 4/10 hypertension; 40mg: 3/9 hypertension
Colhoun 2004 (UK)	Atorvastatin (10mg) [M]	Yes	24, 48, 96, 144, 192 weeks (10mg)	2838	A: 61.5 (SD 8.3) yrs.	68%	95% White (British)	Non-HDL-c, TC, LDL-c, HDL-c	36% > BMI 30kg/m2	Type II	Diagnosed at least 6 months before study	T2D diagnosed at least 6 weeks before study and history of hypertension and retinopathy	NR	**High** (History of hypertension, defined as receiving antihypertensive treatment or having systolic blood pressure of 140 mm Hg or greater or diastolic blood pressure of 90 mm Hg or greater on at least two successive occasions; patients were ineligible if they had any past history of

														myocardial infarction, angina, coronary vascular surgery, cerebrovascular accident, or severe peripheral vascular disease)
DALI Study Group 2001 (Netherlands)	Atorvastatin (10mg [M], 80mg [H])	Yes	3 weeks (10mg) 30 weeks (80mg)	251	60 (7.6) yrs.	>53% (Int groups)	Over 82% White European	TC; LDL-c; HDL-c	≥30 kg/m2	Type II	>11 yrs. in both statin groups	NR	Lipid-lowering drugs were withdrawn at least 8 weeks before the start of the run-in phase	**Low** (Patients were not included in the present study if they had a history of myocardial infarction, percutaneous transluminal coronary angioplasty, coronary artery bypass grafting, proven manifest coronary artery disease, severe or unstable angina pectoris (higher than grade II of the Canadian Cardiovascular Society), clinically manifest heart failure (higher than grade II New York Heart Association), or severe cardiac arrhythmia)
Dallinga-Thie 2009 (Netherlands)	Atorvastatin (80mg) [H]	Yes	30 weeks	81	A: 60 ± 8 years	A: 19F/21M	Over 82% White European	TC; LDL-c; HDL-c	A: 31 (SD 4.7) kg/m2	Type II	At least one year	hypertriglyceridemia	NR	**Low** (Patients with an HbA1c above 10% or a history of cardiovascular disease were excluded)
Ferrer-García 2008 (Spain)	Atorvastatin (10mg [M], 20mg [M], 40mg [M], 80mg [H])	No	24 weeks	202	61.1 (SD 9.9, Range 30-85) yrs.	59.90%	NR	Non-HDL-c, TC, LDL-c, HDL-c	30.1 (SD 4.9) kg/m2	Type II	14.5 (SD 12.8) yrs.	NR	All were statin-naïve with LDL-C levels in excess of 2.6 mmol/L after 6 to 12 weeks	**Medium** (54.4% hypertension, 48% family history of T2Ds, 50% retinopathy)

													of dietary therapy	
Gentile 2000 (Italy)	Atorvastatin (10mg) [M], Pravastatin (20mg) [L], Simvastatin (10mg) [L]	Yes	24 weeks	412	Above 57 yrs. in each group	Over 60% males in each group	NR	TC; LDL-c; HDL-c	Between 28-30 kg/m2	Type II	NR	Excluded patients include: primary hypothyroidism; nephrotic syndrome; type 1 diabetes mellitus; hepatic dysfunction; serum creatine phosphokinase levels > 3 times the upper normal; body mass index > 32 kg/m2; uncontrolled hypertension; myocardial infarction; coronary angioplasty; coronary artery bypass graft; or established CAD	Medications known to affect lipid levels, interact with study medications, or affect clinical laboratory parameters (e.g. erythromycin, anticoagulants, isotretinoin, immune-suppressive agents, lipid-regulating drugs, systemic steroids) we're not allowed during the study	**Low** (Excluded patients with uncontrolled hypertension; myocardial infarction; coronary angioplasty; coronary artery bypass graft; or established CAD; known hypersensitivities to HMG-Cao reductase inhibitors)
Goldberg 2006 (US)	Atorvastatin (10mg [M], 20mg [M], 40mg [H])	No	6 weeks	1229	All A: 59.7 (SD 10.4)	A: 49.1%	A: 75.4% white (western), 10.5% black, 9.4% Hispanic American, 3.5% Asian (any)	Non-HDL-c, TC, LDL-c, HDL-c	A: 33.6 (SD 7.4)	Type II	NR	86.5% metabolic syndrome at baseline	3-5 week washout period of current lipid-lowering medications was done before randomisation	**Medium** (100% CHD or CHD risk equiv.; 15.4% actually had CHD; 11.3% ≥ 2 CHD (10 yrs. >20%) risk factors
Hadjibabaie 2006 (Tehran)	Atorvastatin (10mg) [M], Simvastatin (20mg) [M]	No	12 weeks	53	A: 53 (9); S: 56 (6)	A: 11%M; S: 33%M	100% Asian (Persian Arab)	TC; LDL-c; HDL-c	A: 31 (5); S: 29 (2)	Type II	A: 8 (6) yrs.; S: 11 (4) yrs.	T2D patients with hyperlipidaemia. Patients with hepatic and renal dysfunction, uncontrolled hypothyroidism, Type 1 diabetes mellitus, pregnancy and uncontrolled hypertension were excluded.	Patients with current use of lipid-lowering drugs were excluded at baseline	**Low** (Patients with hepatic and renal dysfunction, uncontrolled hypothyroidism, Type 1 diabetes mellitus, pregnancy, current use of lipid-lowering drugs, women on hormone-replacement

														therapy and uncontrolled hypertension were excluded)
Ichihara 2002 (Japan)	Fluvastatin (20mg) [L]	Yes	12, 24 weeks	22	F: 65.8 (30)	64%	100% Asian (Japanese)	TC, HDL-c	F: 20.9 (0.6)	Type II	Betwee n 2-3 yrs. (34.5 (5.3) months)	No clinical cardiovascular disease, secondary hyperparathyroidi sm or a dynamic bone disease during the 6-months preceding study randomisation	Patients receiving drugs that may affect lipid metabolism were excluded	**Low** (Patients had no clinical cardiovascular disease, secondary hyperparathyroidis m)
Insull 2001 (US)	Atorvastati n (10mg) [M], Simvastatin (10mg) [L]	No	6 weeks	1,424	A: 60.9 (Range 28-81); S: 60.1 (Range 25-82)	A: 51.8%; S: 53.3%	A: 89.3% white (western); S: 92.2% white (western)	TC, LDL-c, HDL-c	A: 31.2; S: 30.9	Type II	NR	Mixed dyslipidaemia with or without CHD/peripheral vascular disease, and with or without type 2 diabetes	NR	**High** (Patients with or without CHD/peripheral vascular disease)
Ishigaki 201 3 (Japan)	Pitavastatin (1mg) [L], Pravastatin (10mg) [L]	No	144 weeks	123	Pit: 59 (8.8), Pra: 60 (9.6)	46%	100% Asian (Japanese)	Non-HDL-c, TC, LDL-c, HDL-c	Pit: 25.4 (4.5), Pra: 26 (3.7)	Type II	NR	Stable control of hyperglycaemia patient included. Patients excluded if they had severe hypertension, a stroke or myocardial infarction event in the past 3 months, heart failure	NR	**Medium** (Patients were excluded if they had severe hypertension (systolic blood pressure (SBP) ≥ 180 mmHg or diastolic BP (DBP) ≥ 110 mmHg) - 40% had hypertension in each group; a stroke or myocardial infarction event in the past 3 months, heart failure, or any allergy or contraindication to statin use as specified in the Japanese Pharmacopoeia)
Janatuinen 2004 (Finland)	Pravastatin (40mg) [L]	Yes	16 weeks	46	P: 30.2 (5.6)	57%	100% White European	TC, LDL-c, HDL-c	P: 24.7 (2.5)	Type I	13.4 (SD 7.7) yrs.	Patient had no symptoms of cardiovascular disease or asthma	Patient did not use cardiovascular medication or	**Low** (young patients with no symptoms of cardiovascular

													antioxidants at recruitment	disease or asthma, no use of cardiovascular medication or antioxidants, no proliferative retinopathy or previous constant microalbuminuria)
Jialal 2007 (US)	Simvastatin (20mg) [M]	Yes	12 weeks	52	S: 23.4 (9.1)	46%	NR	Non-HDL-c, TC, LDL-c, HDL-c	S: 24.9 (4)	Type I	Onset <20 yrs., and on insulin therapy since diagnosis	Type I diabetic patients without clinical macrovascular complications recruited at baseline	NR	**Low** (unclear but patient was young and do not appear to have any history of CHD, angina, hypertension etc.)
Kim 2013 (South Korea)	Pravastatin (20mg, 40mg) [L]	Yes	8 weeks (20/40mg), 16 weeks (20/40mg)	81	20mg: 60 (36-70) yrs.; 40mg: 60 (41-69) yrs.	100% Female	100% Asian (South Korean)	TC, LDL-c, HDL-c	20mg: 26.2 (2.6); 40mg: 26.2 (3.4)	Type II	20mg: 3 (1-10) yrs.; 40mg: 2 (1-9) yrs.	Include: type 2 diabetes mellitus (T2DM) and hypercholesterolemia. Exclude: history of ischemic heart disease or congestive heart failure (NYH class 2) in the preceding 3 months	The concomitant use of medications such as angiotensin converting enzyme inhibitors or other angiotensin II receptor blockers at entry and the use of insulin were exclude. Patients taking thiazolidinedione in the preceding 2 months were also excluded.	**Low** (Exclusion criteria included a history of ischemic heart disease or congestive heart failure (NYH class 2) in the preceding 3 months; proliferative retinopathy or stroke; concomitant use of medications such as angiotensin converting enzyme inhibitors or other angio-tension II receptor blockers at entry and the use of insulin)
Kim 2016 (South Korea)	Atorvastatin (10mg [M], 40mg [H])	No	8 weeks	50	10mg: 56 (11.4); 40mg: 55.2 (13.3)	58%	100% Asian (South Korean)	TC, LDL-c, HDL-c	10mg: 25 (3.1); 40mg: 26.1 (4.6)	Type II	10mg: 4.4 (5.8); 40mg: 5.7 (6.4)	Patients with acute infectious disease and a history of acute myocardial infarctions within 6 months were excluded	Patients with a history of lipid lowering medication within 4 weeks (including the screening	**Low** (Excluded patients were those with a history of lipid lowering medication within 4 weeks (including the screening period),

													period) were excluded.	hypersensitivity with acute infectious disease and a history of acute myocardial infarctions within 6 months)
Knopp 2006 (Mixed Western Countries)	Atorvastatin (10mg) [M]	Yes	4 years	2,410	61.1 (SD 8.1)	66%	84% white (western); 6.7% black (any)	TC, LDL-c, HDL-c	28.9 (SD 3.7)	Type II	8 yrs.	Documented myocardial infarction or an interventional procedure 3 months before screening were excluded.	Subjects taking lipid-altering medications, including other statins, were screened after a 4-week washout phase, except in the case of probucol, which was discontinued for at least 6 months before screening	**Medium** (Myocardial infarction, interventional procedure, or episodes of unstable angina 3 months before screening; HbA1c(A1C) 10%; active liver disease; severe renal dysfunction of nephrotic syndrome; congestive heart failure treated with digoxin; creatine phosphokinase 3 the upper limit of normal; blood pressure 160/100mmHg; BMI 35 kg/m2; abuse of alcohol and/or drugs; hypersensitivity to the study medication; participation in another clinical study within 30 days of screening)
Koh 2008 (South Korea)	Simvastatin (10mg [L], 20mg [M], 40mg [M], 80mg [H])	Yes	8 weeks	156	between 57-60 (SD 2)	47%	100% Asian (South Korean)	TC, LDL-c, HDL-c	Between 25 - 27	Type II	NR	Patients with hypercholesterolemia included. Patient with severe hypertension, stroke, acute coronary events, coronary	No patient had taken any lipid-lowering agent or antioxidant vitamin supplements or had	**Low** (Excluded patients with overt liver disease, chronic renal failure, hypothyroidism, myopathy, uncontrolled diabetes, severe

												revascularization within the preceding 3 months were excluded.	undergone hormone replacement therapy during the 2 months preceding the study.	hypertension, stroke, acute coronary events, coronary revascularization within the preceding 3 months, or alcohol abuse)
Koh 2009 (South Korea)	Pravastatin (40mg) [L], Simvastatin (20mg) [M]	Yes	8 weeks	127	S: 58 (2); P: 56 (2)	38%	100% Asian (South Korean)	TC, LDL-c, HDL-c	S: 25.25 (0.53); P: 25.48 (0.48)	Type II	NR	Patients with hypercholesterolemia (low-density lipoprotein cholesterol levels ≥130mg/dl) participated. Patients with overt liver disease, chronic renal failure, hypothyroidism, myopathy, uncontrolled diabetes, severe hypertension, stroke, acute coronary events, coronary revascularization within the preceding 3 months, or alcohol abuse were excluded	No patient had taken any lipid-lowering agent, hormone replacement therapy, or antioxidant vitamin supplements during the 2 months preceding our study	**Medium** (We excluded patients with overt liver disease, chronic renal failure, hypothyroidism, myopathy, uncontrolled diabetes, severe hypertension, stroke, acute coronary events, coronary revascularization within the preceding 3 months, or alcohol abuse)
Koh 2016 (South Korea)	Rosuvastatin (5mg [M], 10mg [M], 20mg [H])	Yes	8 weeks	190	Between 56-58 yrs. (SD: 7-8)	50%	100% Asian (South Korean)	Non-HDL-c, TC, LDL-c, HDL-c	Between 24.98 - 25.64	Type II	NR	More than half patients were hypertensive. There were some patients with stable angina in each group. Patients with overt liver disease, chronic renal failure, hypothyroidism, myopathy, uncontrolled diabetes, severe	No patient had taken any lipid-lowering agent, hormone replacement therapy, or antioxidant vitamin supplements during the 2 months preceding the study	**Medium** (>60% Hypertension)

												hypertension, stroke, unstable angina, acute myocardial infarction, coronary revascularization within the pre-ceding 3 months, or alcohol abuse were excluded		
Lawrence 2004 (UK)	Atorvastatin (80mg) [H]	Yes	8 weeks	40	63 (9)	53%	NR	TC, LDL-c, HDL-c	31.1 (5.6)	Type II	NR	Total cholesterol >5 mmol/L at recruitment	Patients had to be on oral hypoglycaemic agents in monotherapy or combination therapy with metformin and/or a sulphonylurea with an HbA1c <10%. Patients were excluded if prescribed insulin or a thiazolidinedione (pioglitazone or rosiglitazone)	**Low** (This included patients with manifest macrovascular disease and those with a calculated coronary heart disease (CHD) risk over the next 10 years of greater than 30% in line with current recommendations of the Joint British Societies Guidelines on Prevention of Coronary Heart Disease)
Lewin 2004 (US)	Simvastatin (40mg) [M]	Yes	24 weeks	233	55 (Range 27-78)	52%	46.3% white (western); 30.1% Hispanic; 6.5% black; 17.1% other	Non-HDL-c, TC, LDL-c, HDL-c	32 (5.4)	Type II	NR	Participants had glycosylated haemoglobin (HbA1c) value ≤9.0% and an LDL-C concentration >100 mg/dL at recruitment	Eligible patients had been taking a stable dosage of pioglitazone (15–45 mg/d) or rosiglitazone (2–8 mg/d) for ≥3 months before screening	**Medium** (Patient had a history of hyperlipidaemic pancreatitis, CHD, or other atherosclerotic disease and a diagnosis of type 1 DM, type I or IV hyperlipidaemia, or homozygous familial hypercholesterolemia)

Liu 2013 (Taiwan)	Atorvastatin (10mg) [M], Pitavastatin (2mg) [M]	No	4 weeks (2/10mg), 12 weeks (2/10mg)	225	58.7 (8.6)	61.80%	100% Asian (Taiwanese)	Non-HDL-c, TC, LDL-c, HDL-c	26.4 (3.5)	Type II	NR	Coronary artery disease 78 participants (34.7%)<br><br>Hypertension 17 participants (75.6%)	During the study period, fibrates, other statins, probucol, and cyclosporine were prohibited. No other medication was reported.	**High** (Patient were considered "high-risk", thus they had to meet at least one of the following criteria (NCEP ATP III guideline): 1) documented CHD; 2) type 2 DM; 3) if the patient had fewer than 2 risk factors (other than LDL) present in the following items without CHD or a CHD risk equivalent, a 10-year (short-term) CHD risk had to be assessed with a Framingham score > 20%)
Miller 2004 (US)	Simvastatin (40mg [M], 80mg [H])	No	6 weeks	151	58.9 (9.8)	71%	89% white (western); 6% black; 1% Asian; 3% other	Non-HDL-c, TC, LDL-c, HDL-c	33.4 (6.2)	Type II	6.4 (6) years	Patients with uncontrolled hypertension (systolic > 160 systolic or diastolic > 100 mm Hg) or a history of recent (within 3 months of randomization) acute coronary syndrome were excluded	The most commonly used medications were metformin (50%), rosiglitazone (20%), glyburide (17%), glipizide (15%), pioglitazone hydrochloride (13%), and glyburide-metformin (11%)	**Low** (patients with uncontrolled hypertension (systolic > 160 systolic or diastolic > 100 mmHg) or a history of recent (within 3 months of randomization) acute coronary syndrome were exclude)
Mori 2013 (Japan)	Atorvastatin (10mg) [M], Pravastatin (10mg) [L], Rosuvastati	No	12 weeks	128	A: 63.2 (1.5); R: 62.5 (1.6); P: 66.2 (1.5)	46%	100% Asian (Japanese)	Non-HDL-c, TC, LDL-c, HDL-c	A: 24.5 (0.6); R: 26 (0.6); P: 24.3 (0.6)	Type II	NR	Patients with a history of stroke or ischemic heart disease during the	Patients already using statins were enrolled if their eligibility was confirmed	**Low** (Patients with a history of stroke or ischemic heart disease during the previous 6 months, patients with liver diseases, patients

	n (5mg) [M]											previous 6 months were excluded	after 1 month or more of washout following signing the consent form	with nephropathy were exclude from the study)
Nora 2003 (Italy)	Atorvastatin (10mg) [M]	Yes	48 weeks	25	66 (8)	60%	NR	TC, LDL-c, HDL-c	30 (3.2)	Type II	10 (4) yrs.	Inclusion criteria were HbA1c less than 9%, absence of macroangiopathic complications (no personal history of major cardiovascular or cerebrovascular events, normal resting ECG, and LDL-cholesterol < 180 mg/dl. Patients assuming ACE- inhibitors were excluded from the study	14 patients were treated with a combination of sulphonylurea plus metformin; 3 with metformin and 7 with sulphonylurea only	**High** (Age above 70, Duration of diabetes over 10 yrs., hypertension present in over 50% of patients, and obese)
Paolisso 1991 (Italy)	Atorvastatin (5mg) [L], Simvastatin (10mg [L], 30mg [M])	Yes	3 weeks (30mg), 8 weeks (5mg, 10mg)	12	72.3 (1.4)	50%	NR	TC, LDL-c, HDL-c	27.2 (0.6)	Type II	8.5 (0.9)	Patients were moderately obese, hypercholesteraemic, non-insulin dependent (Type II) diabetic	The patients stopped taking oral hypoglycaemic agents or other drugs at least 3 weeks before the experiments	**Moderate** (unclear but due to high mean age and moderate obese nature of patients coded as high)
Schneider 2004 (Germany)	Atorvastatin (40mg) [H]	Yes	8 weeks	61	61.4 (8.2)	61%	100% White European	TC, LDL-c, HDL-c	29.2 (4.1)	Type II	19.3 (11.4)	Patients with fasting triglyceride level >11.4mmol/L (1.000 mg/dl) were excluded	Exclusion criteria were intravenous or subcutaneous heparin treatment in the 72h before the study, contraindications for heparin or statin treatment, and lipid lowering	**High** (Unclear but age and duration of diabetes diagnosis would suggest high risk of CVD and MACE outcomes)

													therapy in the last 3 months	
Sever 2005 (UK, Ireland, Nordic countries)	Atorvastatin (10mg) [M]	Yes	24, 48, 96, 144 weeks	2532	63.6 (8.5)	77%	90% White western	TC, LDL-c, HDL-c	30.3 (5)	Type II	NR	Patients with previous myocardial infarction, currently treated angina, a cerebrovascular event within the previous 3 months, fasting triglyceride level 4.5 mmol/l, heart failure, un-controlled arrhythmias, or any clinically important haematological or biochemical abnormality on routine screening were excluded	At randomization, 52% of the diabetic patients were receiving oral hypoglycaemic drugs, and 7% were receiving insulin	**Medium** (<8% previous stroke/ischemic heart attack, <6% vascular disease; <4% other CVD; number of risk factors (inc. diabetes), 4.1 (1)
Sindhu 2011 (India)	Atorvastatin (40-80mg) [H], Rosuvastatin (10-40mg) [H]	No	24 weeks	40	A: 49 (8.99); R: 49.1 (6.82)	65%	100% Asian (Indian)	TC, LDL-c, HDL-c	A: 32.15 (1.40); R: 32.1 (1.72)	Type II	NR	Patients with significant cardiovascular dysfunction were excluded	NR	**Medium** (unclear but relatively Middle Aged and no mention of MACE outcomes regardless being obese and T2D diagnosed)
Son 2013 (South Korea)	Atorvastatin (10mg [M], 20mg [M], 40mg [H])	No	8 weeks	474	10mg: 58.47 (11.20); 20mg: 59.37 (9.51); 40mg: 58.87 (9.09)	10mg: 45.65%; 20mg: 38.33%; 40mg: 29.17%	100% Asian (South Korean)	Non-HDL-c, TC, LDL-c, HDL-c	10mg: 25.41 (3.49); 20mg: 25.75 (4.92); 40mg: 25.97 (3.27)	Type II	10mg: 6.45 (6.77); 20mg: 6.32 (6.57); 40mg: 4.54 (5.81)	Patients were excluded if they had a history of CHD, cerebrovascular disease or peripheral vascular disease	Medications known to affect lipid levels or to interact with the medications used in this trial were prohibited for the duration of the study.	**Low** (Patients were statin free at start of trial, and were exclude if they had a history of CHD, cerebrovascular disease or peripheral vascular disease, impaired hepatic or renal function, elevation of creatinine kinase levels, uncontrolled hypertension, uncontrolled hypothyroidism, alcohol or any

														other drug abuse, a history of hypersensitivity to statins)
Tekin 2008 (Turkey)	Simvastatin (40mg) [M]	Yes	6, 12 weeks	90	Between 52 - 55	Between 57% - 67%	100% Asian (Turkish)	TC, LDL-c, HDL-c	Betwee n 26 - 30 kg/m2	Type I or II	NR	Patients excluded if they had heart failure, left bundle-branch block, cardiac rhythm other than sinus, presence of significant vascular disease and myocardial dysfunction on transthoracic echocardiography, previous therapy with lipid lowering medications and clinical evidence of coronary artery disease defined as history of angina, myocardial infarction, percutaneous coronary intervention or bypass surgery.	Concomitant medications aspirin, angiotensin-converting enzyme inhibitor, angiotensin-receptor blocker beta-blocker Diuretic, oral antidiabetics Insulin	**Low** (younger age of patients, and exclusion criteria being uncontrolled hypertension, heart failure, left bundle-branch block, cardiac rhythm other than sinus, presence of significant vascular disease and myocardial dysfunction on transthoracic echocardiography, previous therapy with lipid lowering medications and clinical evidence of coronary artery disease defined as history of angina, myocardial infarction, percutaneous coronary intervention, or bypass surgery)
Thongtang 2020 (Thailand)	Atorvastatin (40mg) [H], Simvastatin (≤ 20mg) [L]	No	6 weeks	150	58.8 (8.9)	28%	100% Asian (Thai)	TC, LDL-c, HDL-c	27.6 (4.5)	Type II	NR	Patients having established ASCVD were excluded	Patients with a history of receiving a stable dose of simvastatin up to 20 mg/day for at least 3 months prior to the start of the study were included at enrolment	**High** (included high intensity statin group as mentioned in paper; receiving a stable dose of simvastatin up to 20 mg/day for at least 3 months prior to the start of the study, and plasma LDL-C level less than100 mg/dl at the time of randomization)

Visseren 2001 (Netherlands)	Fluvastatin (40mg) [L]	Yes	12, 24 weeks	42	60 (8)	76%	95% White European	TC, LDL-c, HDL-c	28.2 (3.5)	Type II	11.1 (8.3) yrs.	Patients were excluded from the study if they had a history of myocardial infarction or coronary angioplasty within 6 months prior to the study, severe congestive heart failure (NYHA grade II-IV), unstable angina pectoris, poorly controlled severe hypertension	NR	**High** (48% had history of CAD, long duration of T2D, almost obese, over 60 yrs. of age, already in receipt of stable insulin therapy)
Winkler 2002 (Germany)	Fluvastatin (80mg) [M]	Yes	8 weeks	89	68 (10)	55%	NR	TC, LDL-c, HDL-c	30.5 (4.1)	Type II	Diagnosed at least 12 weeks before start of trial	Exclusion criteria included among others myocardial infarction, or angioplasty during the 6 months before randomization	Patients receiving lipid-lowering therapy during the 8 wk. before the study; and use of insulin and oral contraceptives at the start of the study were excluded	**High** (31% history of CHD, 10% CVD, 69% hypertension, over 60 yrs. and obese)
Wolffenbuttel 2005 (Netherlands)	Atorvastatin (20mg [M], 40mg [H], 80mg [H]), Rosuvastatin (10mg, 20mg, 40mg) [H]	No	6 weeks	263	60 (10)	46%	NR	Non-HDL-c, TC, LDL-c, HDL-c	31.4 (6.1)	Type II	NR	Subjects with active cardiovascular disease (uncontrolled hypertension >200/ >95 mmHg), heart failure NYHA class IV, recent unstable AP, myocardial infarction, transient ischaemic attack, cerebrovascular accident, coronary artery bypass surgery or angioplasty within	Concomitant treatment with erythromycin, clarithromycin, azole antifungal agents, cyclosporin, antiviral agents, phenytoin, carbamazepine, phenobarbital, or nefazodone, was prohibited	**Low** (2% angina; 6% current angina pectoris; 0% percutaneous transluminal coronary angioplasty; 2% coronary artery bypass surgery; 2% cerebrovascular accident; 2% transient ischaemic attack)

												the previous 2 months, or likely to undergo coronary artery intervention within 6 months after randomization were not eligible		
Xu 2007 (China)	Atorvastatin (20mg) [M]	Yes	84 weeks	648	NR	NR	100% Asian (Chinese)	Non-HDL-c, TC, LDL-c, HDL-c	NR	Type I or II	9.4 yrs.	Patients had both diabetes and coronary artery disease	Treatment for diabetes was maintained during the study: insulin (n=267), sulfonylureas (n=101), alpha-glucosidase inhibitors (n=137), nateglinide (n=107). The proportion of patients taking other cardiovascular drugs, such as acetylsalicylic acid, clopidogrel, beta-blockers, calcium antagonists, nitrates and angiotensin-converting enzyme inhibitors after PCI was similar between the groups (data not shown)	**High** (Involved patients with history of CAD and undergone percutaneous coronary intervention)
Zhang 1995 (Belgium)	Pravastatin (20mg) [L]	Yes	12 weeks	20	43 (Range	80%	NR	TC, LDL-c, HDL-c	NR	Type I or II	21 (4-44) yrs.	Nine patients had peripheral	All groups received	**High** (10, 50% of patients had CHD)

					29-61) yrs.							arteriosclerosis diagnosed by Doppler; 11-patients had hypertension, and 10 patients had coronary heart disease identified by positive ECG change	insulin therapy throughout the trial	

*Intensities defined as: L: Low; M: Moderate and H: High. ¥Gender abbreviations: M: Males; F: Females
ᶲThe patient-risk classifications were initially categorised as 'low/moderate/high' risk according to baseline and inclusion/exclusion criteria within the original report. We then categorised into the following two groups for the subgroup analysis: (i) high risk patients involving those with a history of MACE outcomes (i.e., nonfatal stroke, nonfatal MI, CHD or CVD), and (ii) low-to-moderate risk patients involving those that have not experienced a previous or current MACE outcome at baseline.
€Ethnicity was classified using the office for national statistics guidance: https://www.ons.gov.uk/methodology/classificationsandstandards/measuringequality/ethnicgroupnationalidentityandreligion#ethnic-group
N: not reported; TC: total cholesterol; LDL-C: low-density lipoprotein- cholesterol; HDL-C: high-density lipoprotein cholesterol. Yrs: Years.

Appendix 5: Risk of Bias Assessment

Study	1. Randomization process	2. Deviations from intended interventions	3. Missing outcome data	4. Measurement of the outcome	5. Selection of the reported result	6. Overall Bias*
**Behounek 1994**	Some concerns	Low	Some concerns	High	High	3
**Berberoglu 2009**	Low	High	Some concerns	Low	Low	2
**Betteridge & Gibson 2007**	Low	Low	Low	Some concerns	Low	1
**Chang 2013**	Some concerns	Some concerns	Some concerns	High	High	3
**Cheung 2005**	Some concerns	Some concerns	Some concerns	High	Low	2
**Chu 2008**	High	High	Some concerns	Low	Some concerns	3
**Colhoun 2004**	Low	Low	Low	Low	Low	1
**DALI Study Group 2001**	Some concerns	Some concerns	Low	Low	Low	1
**Dallinga-Thie 2009**	High	Some concerns	Some concerns	Some concerns	High	3
**Ferrer-García 2008**	Some concerns	Some concerns	Low	Low	Low	2
**Gentile 2000**	Some concerns	Some concerns	Low	High	Some concerns	3
**Goldberg 2006**	Some concerns	Low	Low	Low	Low	1
**Hadjibabaie 2006**	Some concerns	Some concerns	High	High	High	3
**Ichihara 2002**	Some concerns	Low	High	High	High	3
**Insull 2001**	Low	Some concerns	Low	Some concerns	Low	1
**Ishigaki 2013**	Low	Some concerns	Some concerns	Low	Low	1
**Janatuinen 2004**	Some concerns	Some concerns	Low	Low	Low	1
**Jialal 2007**	Some concerns	Low	Some concerns	Low	Low	1
**Kim 2013**	Low	Some concerns	Low	Low	Low	1
**Kim 2016**	High	High	Some concerns	Low	Low	3
**Knopp 2006**	Some concerns	Low	Low	Low	Low	1
**Koh 2008**	Some concerns	Low	Low	Low	Some concerns	1
**Koh 2009**	Some concerns	Some concerns	Some concerns	Low	Low	2
**Koh 2016**	Low	Low	Some concerns	Low	Some concerns	1
**Lawrence 2004**	Some concerns	Some concerns	Some concerns	High	Some concerns	2

**Lewin 2004**	Some concerns	Some concerns	High	Low	Low	2
**Liu 2013**	Low	Low	Low	Low	Low	1
**Miller 2004**	Low	Some concerns	Low	High	Some concerns	2
**Mori 2013**	Some concerns	Some concerns	High	Low	Low	2
**Nora 2003**	Some concerns	Some concerns	Low	Some concerns	Some concerns	2
**Paolisso 1991**	Some concerns	Some concerns	Low	High	Some concerns	3
**Schneider 2004**	Some concerns	Some concerns	Some concerns	Some concerns	Some concerns	2
**Sever 2005**	Low	Low	Some concerns	Low	Low	1
**Sindhu 2011**	Low	Some concerns	Low	Some concerns	Some concerns	1
**Son 2013**	High	High	Some concerns	Low	Low	3
**Thongtang 2020**	Low	Some concerns	Low	Low	Low	1
**Visseren 2001**	Some concerns	Low	Low	Low	Some concerns	1
**Winkler 2002**	Some concerns	Some concerns	High	Low	Low	1
**Wolffenbuttel 2005**	Low	Some concerns	Low	Low	Some concerns	1
**Xu 2007**	Some concerns	High	Some concerns	High	High	3
**Zhang 1995**	High	High	Some concerns	High	High	3

*The overall risk of bias judgement was classified as follows: ‘low = 1’: when study was judge to be at low risk of bias for all domains with some concerns showing; ‘Some concerns = 2’: when the study is judged to raise more domains with at least some concerns or high risk of bias in at least one domain; ‘High = 3’: Study is judged to be at high risk of bias in at least one domain and/or to have some concerns for multiple domains in a way that substantially lowers confidence in the result.

Risk of Bias contribution by intervention group

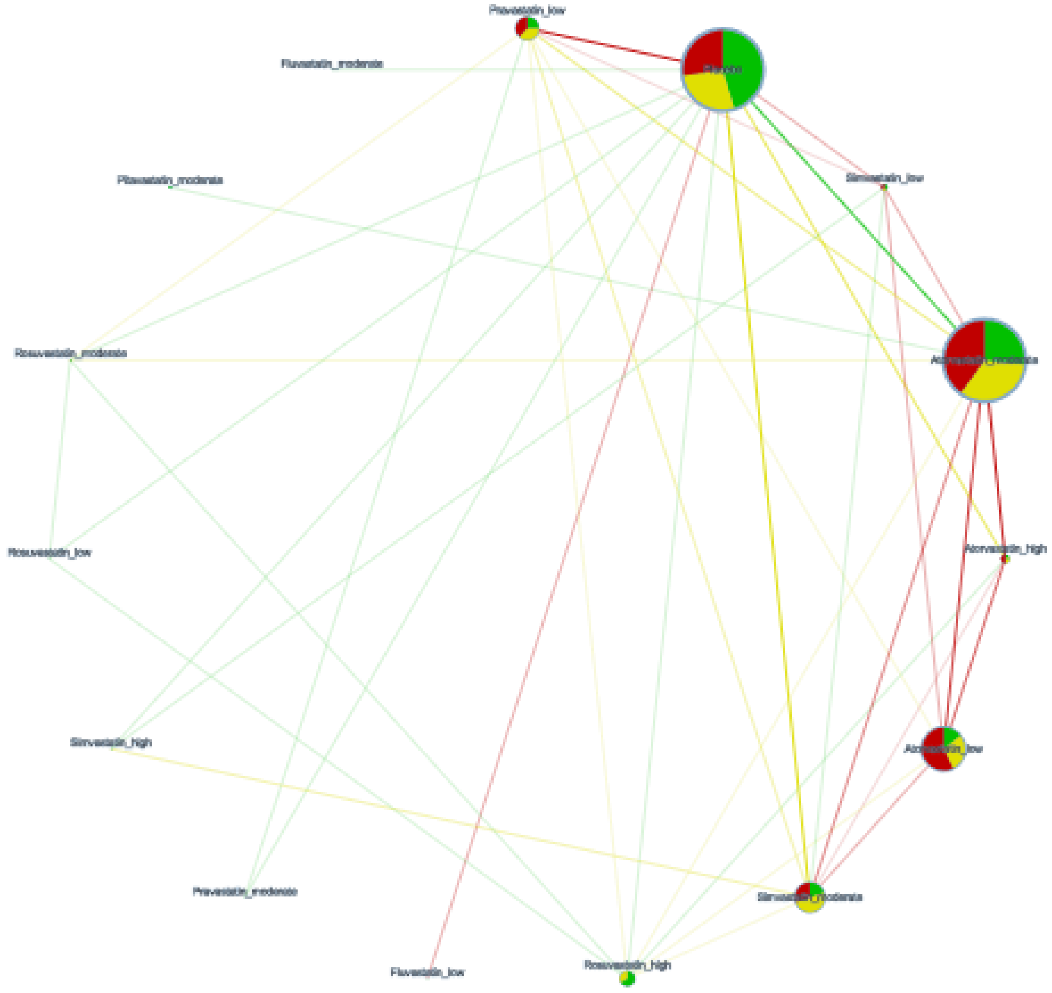

Overall risk of bias by treatment comparison

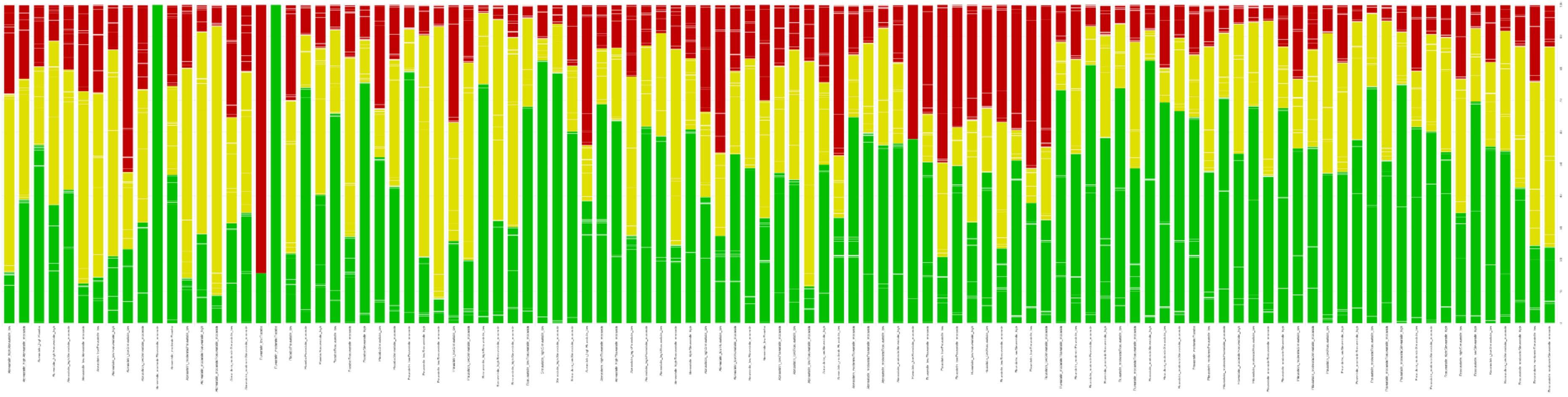

Appendix 6: Deviance statistics of convergence model, Mean Posterior values by Dose and Meta-Analysis of Direct (Pairwise) Evidence

Random effects leverage plot with deviance statistics for non-HDL-C convergence model

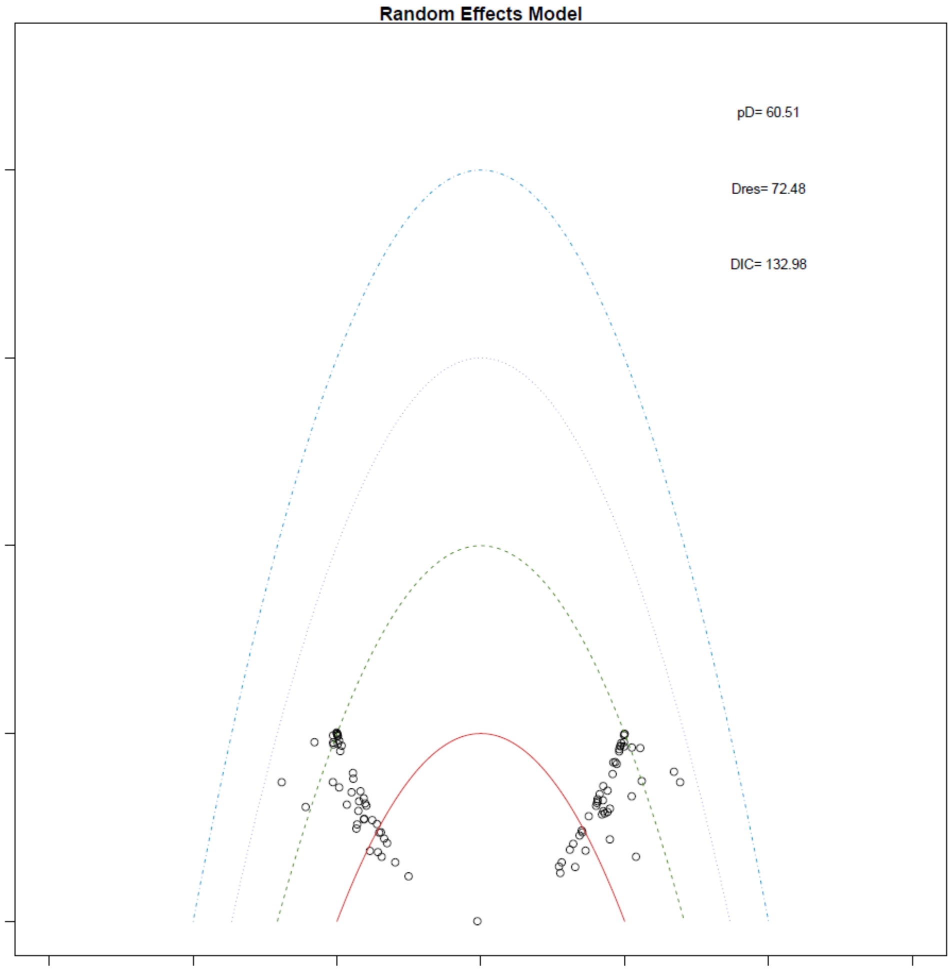

Box Plots of Posterior mean of non-HDL-C by statin dose

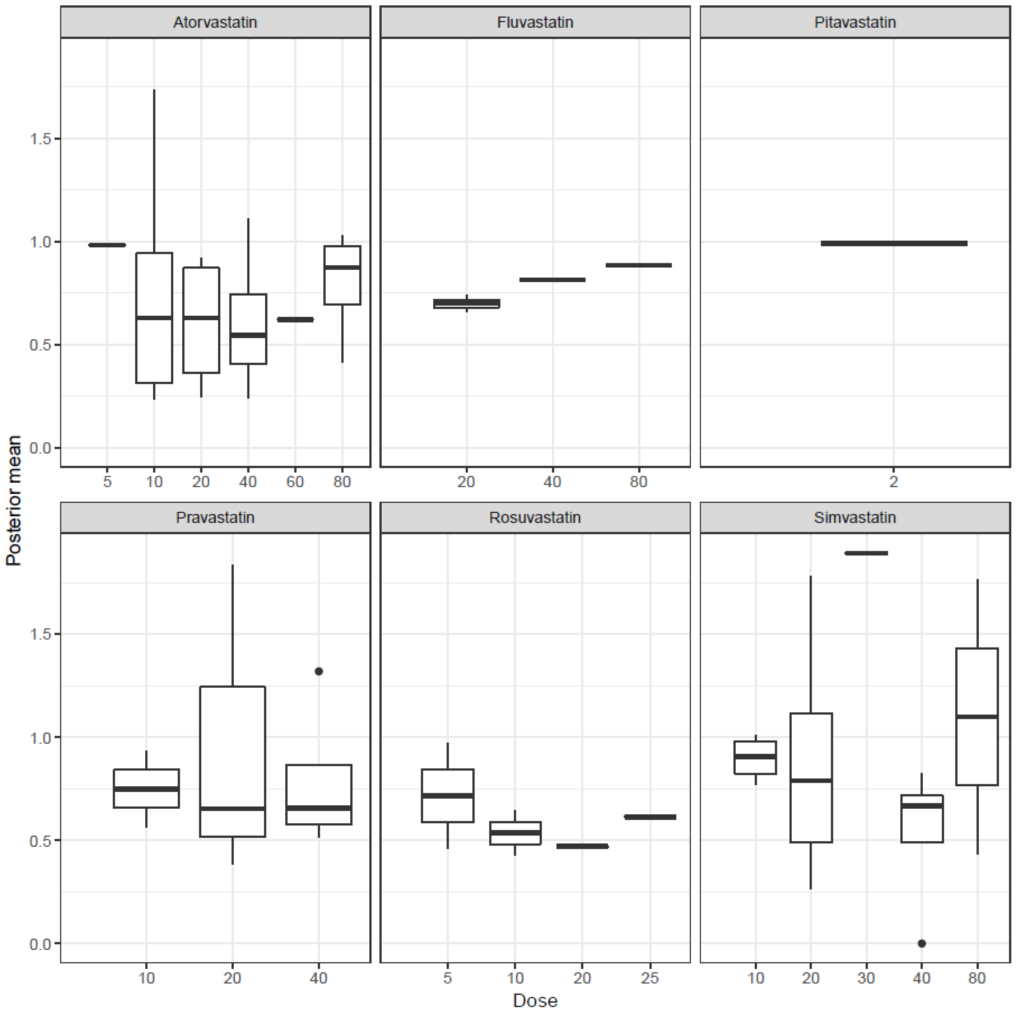

Profile plots of Treatment Response by Dose for non-HDL-C

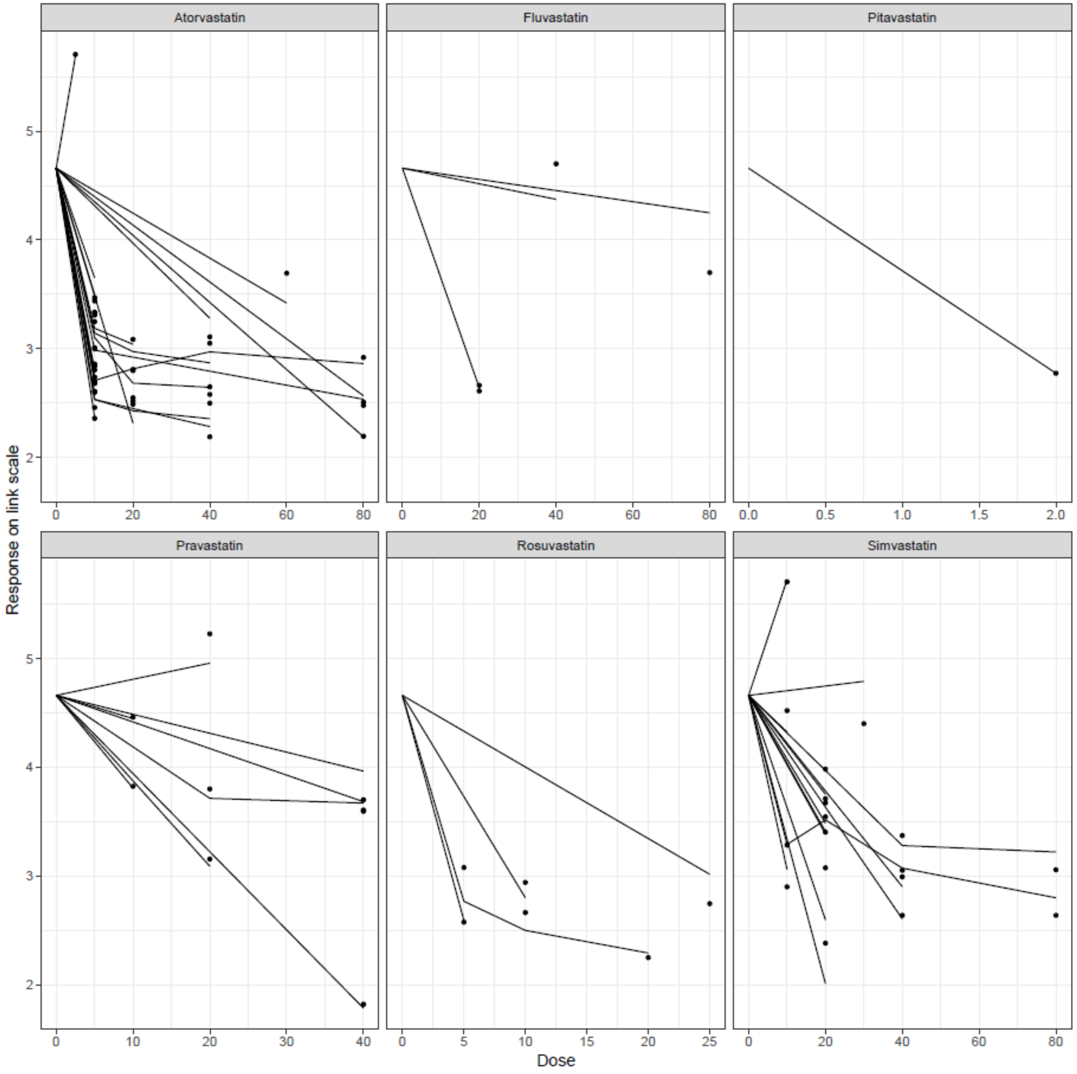

Meta-analysis of all direct (pairwise) evidence for non-HDL-C including the number of studies and number of patients

Comparisons	No. of studies	No. of Patients	Effect size (95% CI)
Atorvastatin_high vs. Atorvastatin_low	4	1175	0.07 (-0.64 to 0.77)
Atorvastatin_high vs. Atorvastatin_moderate	6	649	0.16 (-0.17 to 0.49)
Atorvastatin_high vs. Placebo	4	305	-2.44 (-2.73 to -2.16)
Atorvastatin_high vs. Rosuvastatin_high	1	35	0.95 (-2.05 to 3.94)
Atorvastatin_high vs. Simvastatin_moderate	1	377	-1.21 (-3.66 to 1.24)
Atorvastatin_low vs. Atorvastatin_moderate	5	2781	0.10 (-0.36 to 0.56)
Atorvastatin_low vs. Pravastatin_low	1	1050	-0.36 (-2.30 to 1.59)
Atorvastatin_low vs. Rosuvastatin_high	1	1067	-0.31 (-2.00 to 1.39)
Atorvastatin_low vs. Simvastatin_low	2	280	-0.01 (-0.35 to 0.34)
Atorvastatin_low vs. Simvastatin_moderate	2	2152	-0.20 (-1.86 to 1.47)
Atorvastatin_moderate vs. Pitavastatin_moderate	1	225	0.07 (-2.10 to 2.24)
Atorvastatin_moderate vs. Placebo	5	4140	-2.00 (-2.26 to -1.73)
Atorvastatin_moderate vs. Pravastatin_low	3	1691	-1.29 (-2.00 to -0.57)
Atorvastatin_moderate vs. Rosuvastatin_high	1	1463	-0.15 (-2.00 to 1.71)
Atorvastatin_moderate vs. Rosuvastatin_moderate	1	79	0.23 (-0.11 to 0.57)
Atorvastatin_moderate vs. Simvastatin_low	2	188	-0.84 (-1.86 to 0.18)
Atorvastatin_moderate vs. Simvastatin_moderate	3	1882	-0.59 (-2.04 to 0.85)
Fluvastatin_low vs. Placebo	2	109	-0.07 (-1.07 to 0.93)
Fluvastatin_moderate vs. Placebo	1	89	-1.43 (-4.31 to 1.45)
Placebo vs. Pravastatin_low	6	686	-1.00 (-1.70 to -0.31)
Placebo vs. Pravastatin_moderate	1	45	-0.90 (-3.33 to 1.53)
Placebo vs. Rosuvastatin_high	1	95	-1.99 (-4.34 to 0.36)
Placebo vs. Rosuvastatin_low	1	95	-1.16 (-3.77 to 1.44)
Placebo vs. Rosuvastatin_moderate	1	96	-1.58 (-3.98 to 0.83)
Placebo vs. Simvastatin_high	1	63	-2.35 (-2.82 to -1.89)
Placebo vs. Simvastatin_low	2	226	-1.47 (-2.03 to -0.92)
Placebo vs. Simvastatin_moderate	6	537	-1.88 (-2.29 to -1.47)
Pravastatin_low vs. Pravastatin_moderate	1	53	0.10 (-2.07 to 2.27)
Pravastatin_low vs. Rosuvastatin_high	1	1059	-0.67 (-2.36 to 1.03)
Pravastatin_low vs. Rosuvastatin_moderate	1	75	-1.25 (-1.59 to -0.90)
Pravastatin_low vs. Simvastatin_low	1	159	-0.71 (-1.50 to 0.09)
Pravastatin_low vs. Simvastatin_moderate	2	1149	-0.06 (-0.44 to 0.32)

Rosuvastatin_high vs. Rosuvastatin_low	1	94	0.83 (-1.38 to 3.03)
Rosuvastatin_high vs. Rosuvastatin_moderate	1	95	0.41 (-1.55 to 2.38)
Rosuvastatin_high vs. Simvastatin_moderate	1	1081	-0.46 (-2.32 to 1.39)
Rosuvastatin_low vs. Rosuvastatin_moderate	1	95	0.41 (-1.85 to 2.67)
Simvastatin_high vs. Simvastatin_low	1	61	0.65 (0.21 to 1.08)
Simvastatin_high vs. Simvastatin_moderate	2	110	0.41 (-0.02 to 0.84)
Simvastatin_low vs. Simvastatin_moderate	1	61	0.23 (-0.15 to 0.62)

Appendix 7: Inconsistency Analysis

Main Intensity model for non-HDL-C:

Comparison	P-value	MD and 95% CrI
Atorvastatin_high vs. Atorvastatin_low	0.216	
- direct		0.11 (-0.44, 0.76)
- indirect		0.86 (-0.16, 1.8)
- network		0.31 (-0.18, 0.83)
Atorvastatin_high vs. Atorvastatin_moderate	0.85995	
- direct		0.16 (-0.30, 0.61)
- indirect		0.025 (-1.4, 1.4)
- network		0.14 (-0.30, 0.57)
Atorvastatin_high vs. Placebo	0.360325	
- direct		2.4 (1.7, 3.1)
- indirect		2.0 (1.3, 2.7)
- network		2.2 (1.7, 2.7)
Atorvastatin_high vs. Rosuvastatin_high	0.558	
- direct		-0.96 (-4.1, 2.1)
- indirect		0.023 (-1.2, 1.2)
- network		-0.11 (-1.2, 1.0)
Atorvastatin_high vs. Simvastatin_moderate	0.5655	
- direct		1.2 (-1.4, 3.7)
- indirect		0.43 (-0.22, 1.0)
- network		0.46 (-0.15, 1.1)
Atorvastatin_low vs. Atorvastatin_moderate	0.44235	
- direct		-0.14 (-0.73, 0.37)
- indirect		-0.57 (-1.5, 0.42)
- network		-0.18 (-0.66, 0.28)
Atorvastatin_low vs. Pravastatin_low	0.688475	
- direct		0.35 (-1.8, 2.5)
- indirect		0.80 (0.15, 1.4)

- network		0.77 (0.15, 1.4)
Atorvastatin_low vs. Rosuvastatin_high	0.7556	
- direct		-0.34 (-2.2, 1.5)
- indirect		-0.73 (-2.4, 0.97)
- network		-0.43 (-1.6, 0.70)
Atorvastatin_low vs. Simvastatin_low	0.2964	
- direct		-0.027 (-0.74, 0.68)
- indirect		0.52 (-0.28, 1.2)
- network		0.22 (-0.33, 0.73)
Atorvastatin_low vs. Simvastatin_moderate	0.921125	
- direct		0.21 (-1.5, 1.9)
- indirect		0.12 (-0.60, 0.78)
- network		0.15 (-0.50, 0.75)
Atorvastatin_moderate vs. Placebo	0.588575	
- direct		2.0 (1.5, 2.5)
- indirect		2.2 (1.6, 2.7)
- network		2.1 (1.6, 2.5)
Atorvastatin_moderate vs. Pravastatin_low	**0.0709**	
- direct		1.3 (0.68, 1.9)
- indirect		0.36 (-0.38, 1.2)
- network		0.95 (0.46, 1.4)
Atorvastatin_moderate vs. Rosuvastatin_high	0.734925	
- direct		-0.13 (-2.1, 1.9)
- indirect		-0.57 (-2.2, 1.1)
- network		-0.25 (-1.3, 0.85)
Atorvastatin_moderate vs. Rosuvastatin_moderate	0.627325	
- direct		-0.23 (-1.1, 0.63)
- indirect		0.33 (-1.8, 2.4)
- network		-0.21 (-0.91, 0.53)
Atorvastatin_moderate vs. Simvastatin_low	0.3247	
- direct		0.80 (-0.16, 1.7)
- indirect		0.24 (-0.44, 0.89)

- network		0.39 (-0.13, 0.92)
Atorvastatin_moderate vs. Simvastatin_moderate	0.7116	
- direct		0.59 (-0.94, 2.1)
- indirect		0.28 (-0.34, 0.88)
- network		0.32 (-0.22, 0.87)
Placebo vs. Pravastatin_low	0.870725	
- direct		-1.0 (-1.6, -0.45)
- indirect		-1.1 (-2.0, -0.25)
- network		-1.1 (-1.6, -0.65)
Placebo vs. Rosuvastatin_high	0.83325	
- direct		-2. (-4.4, 0.49)
- indirect		-2.3 (-3.5, -0.97)
- network		-2.3 (-3.4, -1.2)
Placebo vs. Rosuvastatin_moderate	0.593525	
- direct		-1.6 (-4.1, 0.81)
- indirect		-2.3 (-3.2, -1.5)
- network		-2.3 (-3.0, -1.5)
Placebo vs. Simvastatin_high	0.7958	
- direct		-2.4 (-3.3, -1.4)
- indirect		-2. (-4.7, 0.70)
- network		-2.3 (-3.2, -1.5)
Placebo vs. Simvastatin_low	0.179875	
- direct		-1.5 (-2.1, -0.82)
- indirect		-2.2 (-3.0, -1.3)
- network		-1.7 (-2.2, -1.2)
Placebo vs. Simvastatin_moderate	0.4656	
- direct		-1.9 (-2.4, -1.4)
- indirect		-1.4 (-2.6, -0.24)
- network		-1.7 (-2.2, -1.3)
Pravastatin_low vs. Rosuvastatin_high	0.5029	
- direct		-0.70 (-2.6, 1.2)
- indirect		-1.5 (-3.2, 0.13)

- network		-1.2 (-2.3, -0.068)
Pravastatin_low vs. Rosuvastatin_moderate	0.567575	
- direct		-1.2 (-2.1, -0.39)
- indirect		-0.58 (-2.7, 1.6)
- network		-1.2 (-1.9, -0.43)
Pravastatin_low vs. Simvastatin_low	0.712925	
- direct		-0.71 (-1.7, 0.30)
- indirect		-0.49 (-1.1, 0.14)
- network		-0.55 (-1.2, 0.039)
Pravastatin_low vs. Simvastatin_moderate	**0.030675**	
- direct		-0.061 (-0.76, 0.64)
- indirect		-1.2 (-1.9, -0.47)
- network		-0.62 (-1.2, -0.085)
Rosuvastatin_high vs. Rosuvastatin_moderate	0.6747	
- direct		0.46 (-1.7, 2.5)
- indirect		-0.079 (-1.5, 1.4)
- network		0.043 (-1.2, 1.2)
Rosuvastatin_high vs. Simvastatin_moderate	0.742825	
- direct		0.47 (-1.5, 2.5)
- indirect		0.90 (-0.85, 2.6)
- network		0.57 (-0.56, 1.7)
Simvastatin_high vs. Simvastatin_low	0.8074	
- direct		0.64 (-0.24, 1.5)
- indirect		0.29 (-2.4, 3.0)
- network		0.59 (-0.16, 1.4)
Simvastatin_low vs. Simvastatin_moderate	0.606875	
- direct		-0.23 (-1.1, 0.65)
- indirect		0.066 (-0.78, 0.85)
- network		-0.069 (-0.65, 0.50)

CrI: credible interval

Patient Risk Adjusted Model for non-HDL-C:

Comparison	P-value	MD and 95% CrI
Atorvastatin_high_HR vs. Atorvastatin_low_HR	0.8215	
- direct		0.94 (-1.8, 3.8)
- indirect		1.5 (-2.9, 5.9)
- network		0.92 (-1.2, 3.2)
Atorvastatin_high_HR vs. Atorvastatin_mod_HR	0.619	
- direct		0.35 (-2.3, 3.0)
- indirect		1.5 (-2.3, 4.8)
- network		0.79 (-1.2, 2.7)
Atorvastatin_high_HR vs. Placebo	0.653	
- direct		2.4 (-0.75, 5.1)
- indirect		1.3 (-1.8, 4.6)
- network		2. (0.052, 4.2)
Atorvastatin_high_LR vs. Atorvastatin_low_LR	0.20475	
- direct		0.081 (-0.50, 0.75)
- indirect		0.88 (-0.23, 1.9)
- network		0.29 (-0.25, 0.85)
Atorvastatin_high_LR vs. Atorvastatin_mod_LR	0.6185	
- direct		0.15 (-0.31, 0.59)
- indirect		-0.24 (-1.7, 1.3)
- network		0.10 (-0.37, 0.54)
Atorvastatin_high_LR vs. Placebo	0.465	
- direct		2.4 (1.6, 3.2)
- indirect		2.0 (1.2, 2.9)
- network		2.2 (1.7, 2.7)
Atorvastatin_high_LR vs. Rosuvastatin_high_LR	0.53725	
- direct		-0.95 (-3.8, 2.1)
- indirect		0.028 (-1.1, 1.3)
- network		-0.14 (-1.3, 0.98)
Atorvastatin_low_HR vs. Atorvastatin_mod_HR	0.7395	

- direct		-0.62 (-3.5, 2.7)
- indirect		0.0025 (-2.4, 2.3)
- network		-0.17 (-1.9, 1.6)
Atorvastatin_low_HR vs. Simvastatin_low_HR	0.8635	
- direct		-0.18 (-1.8, 1.5)
- indirect		-0.51 (-4.0, 2.6)
- network		-0.21 (-1.6, 1.2)
Atorvastatin_low_LR vs. Atorvastatin_mod_LR	0.37075	
- direct		-0.13 (-0.74, 0.43)
- indirect		-0.65 (-1.8, 0.45)
- network		-0.19 (-0.73, 0.30)
Atorvastatin_low_LR vs. Pravastatin_low_LR	0.66075	
- direct		0.33 (-1.8, 2.5)
- indirect		0.83 (0.15, 1.5)
- network		0.80 (0.16, 1.4)
Atorvastatin_low_LR vs. Rosuvastatin_high_LR	0.802	
- direct		-0.28 (-2.1, 1.7)
- indirect		-0.58 (-2.4, 1.2)
- network		-0.43 (-1.6, 0.69)
Atorvastatin_low_LR vs. Simvastatin_low_LR	0.2685	
- direct		0.00063 (-0.87, 0.84)
- indirect		0.64 (-0.24, 1.5)
- network		0.30 (-0.31, 0.91)
Atorvastatin_low_LR vs. Simvastatin_mod_LR	0.98525	
- direct		0.18 (-2.0, 2.5)
- indirect		0.17 (-0.60, 0.87)
- network		0.17 (-0.52, 0.85)
Atorvastatin_mod_HR vs. Placebo	0.663	
- direct		0.76 (-1.1, 2.8)
- indirect		1.7 (-2., 5.2)
- network		1.2 (-0.51, 3.2)
Atorvastatin_mod_HR vs. Simvastatin_low_HR	0.8685	

- direct		-0.12 (-1.8, 1.6)
- indirect		0.21 (-3.1, 3.3)
- network		-0.040 (-1.5, 1.5)
Atorvastatin_mod_LR vs. Placebo	0.72325	
- direct		2.1 (1.5, 2.6)
- indirect		2.2 (1.6, 2.8)
- network		2.1 (1.7, 2.6)
Atorvastatin_mod_LR vs. Pravastatin_low_LR	0.1185	
- direct		1.3 (0.63, 1.9)
- indirect		0.40 (-0.42, 1.3)
- network		0.98 (0.49, 1.5)
Atorvastatin_mod_LR vs. Rosuvastatin_high_LR	0.74975	
- direct		-0.088 (-2.1, 1.9)
- indirect		-0.49 (-2.1, 1.1)
- network		-0.24 (-1.4, 0.84)
Atorvastatin_mod_LR vs. Rosuvastatin_mod_LR	0.5585	
- direct		-0.24 (-1.2, 0.70)
- indirect		0.51 (-1.8, 2.6)
- network		-0.19 (-0.89, 0.59)
Atorvastatin_mod_LR vs. Simvastatin_low_LR	0.17725	
- direct		1.2 (0.032, 2.4)
- indirect		0.27 (-0.45, 1.0)
- network		0.49 (-0.095, 1.1)
Atorvastatin_mod_LR vs. Simvastatin_mod_LR	0.99425	
- direct		0.34 (-1.7, 2.4)
- indirect		0.37 (-0.28, 1.0)
- network		0.37 (-0.23, 0.97)
Placebo vs. Pravastatin_low_LR	0.765	
- direct		-1.0 (-1.7, -0.42)
- indirect		-1.2 (-2.2, -0.28)
- network		-1.1 (-1.6, -0.62)
Placebo vs. Rosuvastatin_high_LR	0.841	

- direct		-2.4 (-4.4, 0.45)
- indirect		-2.3 (-3.6, -0.98)
- network		-2.4 (-3.4, -1.3)
Placebo vs. Rosuvastatin_mod_LR	0.5605	
- direct		-1.6 (-4.1, 0.94)
- indirect		-2.4 (-3.3, -1.6)
- network		-2.3 (-3.1, -1.5)
Placebo vs. Simvastatin_high_LR	0.8685	
- direct		-2.4 (-3.3, -1.4)
- indirect		-2.1 (-4.7, 0.49)
- network		-2.2 (-3.0, -1.5)
Placebo vs. Simvastatin_low_LR	0.2715	
- direct		-1.5 (-2.1, -0.77)
- indirect		-2.2 (-3.2, -0.96)
- network		-1.6 (-2.2, -1.1)
Placebo vs. Simvastatin_mod_LR	0.72825	
- direct		-1.9 (-2.4, -1.4)
- indirect		-1.6 (-3.0, -0.18)
- network		-1.8 (-2.2, -1.3)
Pravastatin_low_LR vs. Rosuvastatin_high_LR	0.5275	
- direct		-0.70 (-2.7, 1.2)
- indirect		-1.5 (-3.2, 0.12)
- network		-1.2 (-2.3, -0.15)
Pravastatin_low_LR vs. Rosuvastatin_mod_LR	0.5975	
- direct		-1.2 (-2.1, -0.31)
- indirect		-0.62 (-2.8, 1.7)
- network		-1.2 (-1.9, -0.41)
Pravastatin_low_LR vs. Simvastatin_low_LR	0.635	
- direct		-0.69 (-1.7, 0.33)
- indirect		-0.41 (-1.1, 0.26)
- network		-0.49 (-1.1, 0.13)
Pravastatin_low_LR vs. Simvastatin_mod_LR	**0.035**	

- direct		-0.061 (-0.77, 0.66)
- indirect		-1.2 (-2.4, -0.46)
- network		-0.62 (-1.2, -0.079)
Rosuvastatin_high_LR vs. Rosuvastatin_mod_LR	0.66925	
- direct		0.41 (-1.7, 2.4)
- indirect		-0.099 (-1.6, 1.4)
- network		0.052 (-1.1, 1.3)
Rosuvastatin_high_LR vs. Simvastatin_mod_LR	0.70075	
- direct		0.41 (-1.6, 2.5)
- indirect		0.96 (-0.71, 2.7)
- network		0.62 (-0.54, 1.7)
Simvastatin_high_LR vs. Simvastatin_low_LR	0.892	
- direct		0.64 (-0.29, 1.6)
- indirect		0.45 (-2.1, 3.1)
- network		0.63 (-0.20, 1.5)
Simvastatin_low_LR vs. Simvastatin_mod_LR	0.788	
- direct		-0.24 (-1.2, 0.71)
- indirect		-0.058 (-1.0, 0.84)
- network		-0.13 (-0.78, 0.48)

HR: high risk patients; LR: low-to-moderate risk patients.

Main Intensity model for LDL-C:

Comparison	P-value	MD and 95% CrI
Atorvastatin_high vs. Atorvastatin_moderate	0.9045	
- direct		0.17 (-0.16, 0.49)
- indirect		0.13 (-0.50, 0.80)
- network		0.18 (-0.12, 0.48)
Atorvastatin_high vs. Placebo	0.40375	
- direct		1.6 (1.1, 2.1)
-> indirect		1.3 (0.79, 1.8)
-> network		1.4 (1.1, 1.8)
Atorvastatin_high vs. Rosuvastatin_high	0.74975	
- direct		-0.42 (-1.3, 0.44)
- indirect		-0.23 (-1.1, 0.67)
- network		-0.33 (-0.94, 0.28)
Atorvastatin_moderate vs. Pitavastatin_moderate	0.88325	
- direct		0.11 (-0.74, 0.98)
- indirect		0.0099 (-0.95, 0.96)
- network		0.067 (-0.56, 0.70)
Atorvastatin_moderate vs. Placebo	0.08275	
- direct		1.0 (0.60, 1.4)
- indirect		1.5 (1.1, 1.9)
- network		1.3 (0.95, 1.6)
Atorvastatin_moderate vs. Pravastatin_low	0.29875	
- direct		0.85 (0.044, 1.7)
- indirect		0.36 (-0.084, 0.84)
- network		0.49 (0.085, 0.89)
Atorvastatin_moderate vs. Rosuvastatin_moderate	0.979	
- direct		-0.19 (-1.0, 0.62)
- indirect		-0.21 (-1.0, 0.63)
- network		-0.28 (-0.82, 0.27)
Atorvastatin_moderate vs. Simvastatin_low	0.538	

- direct		0.12 (-0.72, 0.94)
- indirect		-0.22 (-1.0, 0.58)
- network		-0.066 (-0.63, 0.50)
Atorvastatin_moderate vs. Simvastatin_moderate	0.22525	
- direct		0.78 (-0.18, 1.8)
- indirect		0.11 (-0.37, 0.59)
- network		0.24 (-0.20, 0.68)
Pitavastatin_moderate vs. Pravastatin_low	0.875	
- direct		0.46 (-0.39, 1.3)
- indirect		0.36 (-0.61, 1.3)
- network		0.42 (-0.22, 1.0)
Placebo vs. Pravastatin_low	0.23	
- direct		-0.89 (-1.3, -0.50)
- indirect		-0.42 (-1.1, 0.27)
- network		-0.76 (-1.1, -0.43)
Placebo vs. Rosuvastatin_high	0.6775	
- direct		-1.6 (-2.5, -0.73)
- indirect		-1.9 (-2.8, -0.92)
- network		-1.8 (-2.4, -1.1)
Placebo vs. Rosuvastatin_moderate	0.64725	
- direct		-1.4 (-2.3, -0.52)
- indirect		-1.6 (-2.4, -0.86)
- network		-1.5 (-2.1, -0.99)
Placebo vs. Simvastatin_low	0.397	
- direct		-1.6 (-2.4, -0.81)
- indirect		-1.1 (-2.0, -0.21)
- network		-1.3 (-1.9, -0.75)
Placebo vs. Simvastatin_moderate	0.1305	
- direct		-1.2 (-1.6, -0.81)
- indirect		-0.37 (-1.4, 0.62)
- network		-1.0 (-1.4, -0.64)
Pravastatin_low vs. Rosuvastatin_moderate	0.4525	

- direct		-1.0 (-1.9, -0.20)
- indirect		-0.58 (-1.5, 0.29)
- network		-0.76 (-1.3, -0.19)
Pravastatin_low vs. Simvastatin_moderate	0.62425	
- direct		-0.084 (-0.88, 0.73)
- indirect		-0.31 (-0.86, 0.25)
- network		-0.25 (-0.73, 0.23)
Rosuvastatin_high vs. Rosuvastatin_moderate	0.99325	
- direct		0.23 (-0.66, 1.1)
- indirect		0.24 (-0.99, 1.4)
- network		0.22 (-0.44, 0.91)
Simvastatin_low vs. Simvastatin_moderate	0.87075	
- direct		0.25 (-0.57, 1.1)
- indirect		0.16 (-0.79, 1.1)
- network		0.30 (-0.29, 0.90)

Appendix 8: Heterogeneity Assessment

Global heterogeneity statistics for each network model of non-HDL-C:

- **Statin Agent and Intensity Model (main model)**
  - Network τ^2 (from NMA in all trials, n=36) = 0 (0 to 0.014)
  - Network I^2 (from NMA in all trials, n=36) = 0 (0 to 38) %
  - DIC = 132.98 (Dbar = 72.49; pD = 60.49)

- **Patient-Risk Adjusted Model**
  - Network τ^2 (from NMA in all trials, n=36) = 0.178 (0.097 to 0.307)
  - Network I^2 (from NMA in all trials, n=36) = 12.1 (0 to 42) %
  - DIC = 142.97 (Dbar = 76.16; pD = 66.80)

Global heterogeneity statistics for network model for LDL-C:

- Network τ^2 (from NMA in all trials, n=29) = 0 (0 to 0.049)
- Network I^2 (from NMA in all trials, n=29) = 5 (0 to 35) %
- DIC = 141.56 (Dbar = 73.70; pD = 67.86)

Heterogeneity score by each comparison for main model of non-HDL-C

Treatment1	Treatment2	I^2 Pairwise	I^2 Network	I^2 Inconsistency (p-value)
Atorvastatin_high	Atorvastatin_low	40.4562303	65.15043	0.12418165
Atorvastatin_high	Atorvastatin_moderate	0	0	0.57929671
Atorvastatin_high	Placebo	0	0	0.31080774
Atorvastatin_high	Rosuvastatin_high	NA	0	0.59711251
Atorvastatin_high	Simvastatin_moderate	NA	0	0.60256453
Atorvastatin_low	Atorvastatin_moderate	17.2586731	45.82898	0.46457183
Atorvastatin_low	Pravastatin_low	NA	0	0.78886846
Atorvastatin_low	Rosuvastatin_high	NA	0	0.92865905
Atorvastatin_low	Simvastatin_low	0	18.27955	0.26160298
Atorvastatin_low	Simvastatin_moderate	0	0	0.99442574
Atorvastatin_moderate	Pitavastatin_moderate	NA	NA	NA
Atorvastatin_moderate	Placebo	0	0	0.67018739
Atorvastatin_moderate	Pravastatin_low	0	0	0.51630449
Atorvastatin_moderate	Rosuvastatin_high	NA	0	0.92681038
Atorvastatin_moderate	Rosuvastatin_moderate	NA	0	0.90146406
Atorvastatin_moderate	Simvastatin_low	0.9475435	0	0.9907888
Atorvastatin_moderate	Simvastatin_moderate	0	0	0.75301716
Fluvastatin_low	Placebo	0	0	NA
Fluvastatin_moderate	Placebo	NA	NA	NA
Placebo	Pravastatin_low	36.5829912	26.23744	0.58383781
Placebo	Pravastatin_moderate	NA	NA	NA
Placebo	Rosuvastatin_high	NA	0	0.86566922
Placebo	Rosuvastatin_low	NA	NA	NA
Placebo	Rosuvastatin_moderate	NA	0	0.73904384
Placebo	Simvastatin_high	NA	0	0.80050655

Placebo	Simvastatin_low	31.4920912	25.44101	0.32659144
Placebo	Simvastatin_moderate	25.4612952	30.79731	0.18489863
Pravastatin_low	Pravastatin_moderate	NA	NA	NA
Pravastatin_low	Rosuvastatin_high	NA	0	0.60983497
Pravastatin_low	Rosuvastatin_moderate	NA	0	0.64728881
Pravastatin_low	Simvastatin_low	NA	0	0.89182508
Pravastatin_low	Simvastatin_moderate	0	74.84326	**0.04960713**
Rosuvastatin_high	Rosuvastatin_low	NA	NA	NA
Rosuvastatin_high	Rosuvastatin_moderate	NA	0	0.71751311
Rosuvastatin_high	Simvastatin_moderate	NA	0	0.9366882
Rosuvastatin_low	Rosuvastatin_moderate	NA	NA	NA
Simvastatin_high	Simvastatin_low	NA	0	0.85089267
Simvastatin_high	Simvastatin_moderate	0	0	NA
Simvastatin_low	Simvastatin_moderate	NA	0	0.56516372

Heterogeneity score Patient-Risk Adjusted Model of non-HDL-C

Treatment1	Treatment2	I^2 Pairwise	I^2 Network	I^2 Inconsistency (p-value)
Atorvastatin_high_HR	Atorvastatin_low_HR	NA	0	0.94383981
Atorvastatin_high_HR	Atorvastatin_mod_HR	NA	0	0.73232802
Atorvastatin_high_HR	Placebo	NA	0	0.62779377
Atorvastatin_high_HR	Simvastatin_mod_HR	NA	NA	NA
Atorvastatin_high_LR	Atorvastatin_low_LR	58.9452122	70.220606	0.21975264
Atorvastatin_high_LR	Atorvastatin_mod_LR	0.9406801	7.046371	0.4877635
Atorvastatin_high_LR	Placebo	0	0	0.48233463
Atorvastatin_high_LR	Rosuvastatin_high_LR	NA	0	0.58493797
Atorvastatin_low_HR	Atorvastatin_mod_HR	NA	0	0.83074732
Atorvastatin_low_HR	Simvastatin_low_HR	NA	0	0.80727772
Atorvastatin_low_HR	Simvastatin_mod_HR	NA	NA	NA
Atorvastatin_low_LR	Atorvastatin_mod_LR	41.9470577	60.018533	0.50790953
Atorvastatin_low_LR	Pravastatin_low_LR	NA	0	0.78464995
Atorvastatin_low_LR	Rosuvastatin_high_LR	NA	0	0.97022176
Atorvastatin_low_LR	Simvastatin_low_LR	NA	72.413919	0.28914319
Atorvastatin_low_LR	Simvastatin_mod_LR	NA	0	0.95139504
Atorvastatin_mod_HR	Pitavastatin_mod_HR	NA	NA	NA
Atorvastatin_mod_HR	Placebo	0	0	0.71407786
Atorvastatin_mod_HR	Simvastatin_low_HR	NA	0	0.85262492
Atorvastatin_mod_HR	Simvastatin_mod_HR	NA	NA	NA
Atorvastatin_mod_LR	Placebo	0	0	0.70128405
Atorvastatin_mod_LR	Pravastatin_low_LR	0	0	0.61672195
Atorvastatin_mod_LR	Rosuvastatin_high_LR	NA	0	0.98681931
Atorvastatin_mod_LR	Rosuvastatin_mod_LR	NA	0	0.86724377
Atorvastatin_mod_LR	Simvastatin_low_LR	NA	0	0.53079951
Atorvastatin_mod_LR	Simvastatin_mod_LR	0	0	0.90106026
Fluvastatin_low_HR	Placebo	NA	NA	NA
Fluvastatin_low_LR	Placebo	NA	NA	NA
Fluvastatin_mod_HR	Placebo	NA	NA	NA

Placebo	Pravastatin_low_HR	NA	NA	NA
Placebo	Pravastatin_low_LR	50.3621219	40.570719	0.52640849
Placebo	Pravastatin_mod_LR	NA	NA	NA
Placebo	Rosuvastatin_high_LR	NA	0	0.82794859
Placebo	Rosuvastatin_low_LR	NA	NA	NA
Placebo	Rosuvastatin_mod_LR	NA	0	0.70878289
Placebo	Simvastatin_high_LR	NA	0	0.79540918
Placebo	Simvastatin_low_LR	31.5269573	11.578625	0.42566386
Placebo	Simvastatin_mod_LR	25.1828725	23.418205	0.3023966
Pravastatin_low_LR	Pravastatin_mod_LR	NA	NA	NA
Pravastatin_low_LR	Rosuvastatin_high_LR	NA	0	0.61234155
Pravastatin_low_LR	Rosuvastatin_mod_LR	NA	0	0.7228183
Pravastatin_low_LR	Simvastatin_low_LR	NA	0	0.80880007
Pravastatin_low_LR	Simvastatin_mod_LR	0	73.161289	**0.07814566**
Rosuvastatin_high_LR	Rosuvastatin_low_LR	NA	NA	NA
Rosuvastatin_high_LR	Rosuvastatin_mod_LR	NA	0	0.73962077
Rosuvastatin_high_LR	Simvastatin_mod_LR	NA	0	0.88101499
Rosuvastatin_low_LR	Rosuvastatin_mod_LR	NA	NA	NA
Simvastatin_high_LR	Simvastatin_low_LR	NA	0	0.95213573
Simvastatin_high_LR	Simvastatin_mod_LR	0	0	NA
Simvastatin_low_LR	Simvastatin_mod_LR	NA	0	0.76579572

Appendix 9: Secondary outcomes (TC, Discontinuations and MACE)

Total cholesterol

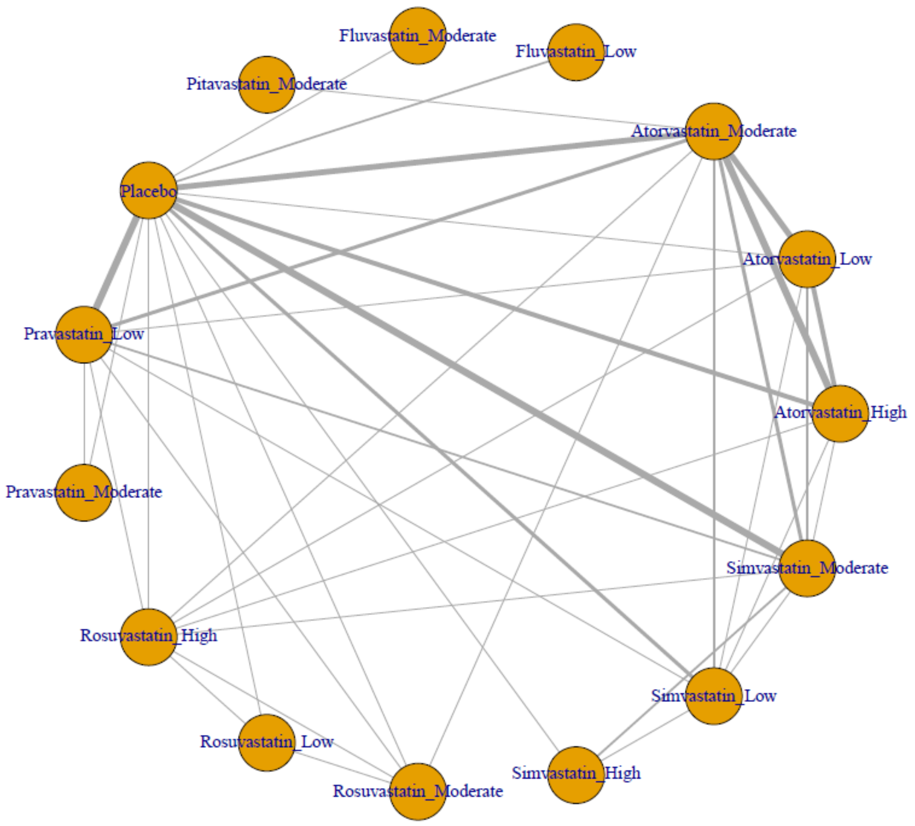

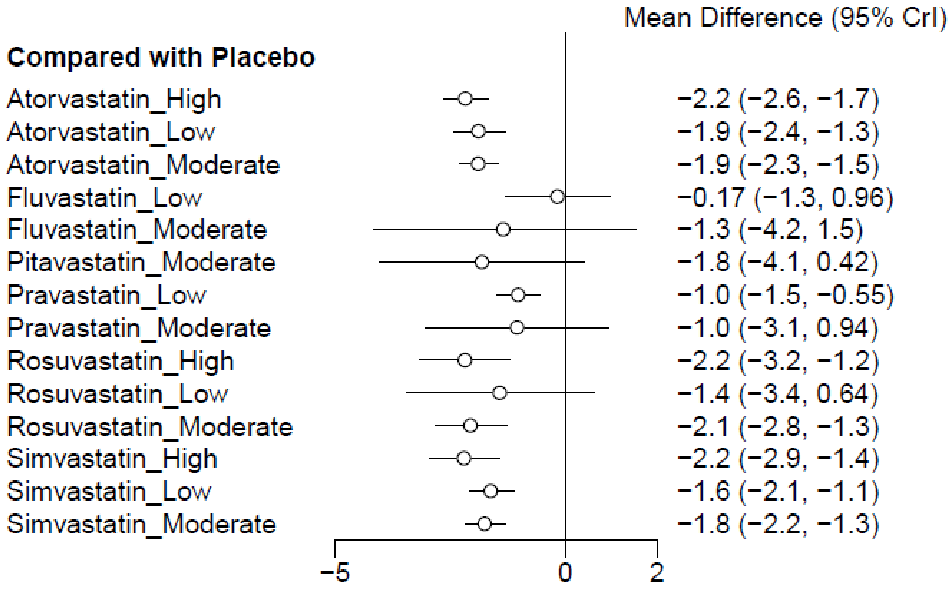

- SUCRA: 1st = Atorvastatin High; 2nd = Simvastatin High; 3rd = Rosuvastatin High
- I^2 = 0%
- One inconsistency detected between Pravastatin low vs. Simvastatin moderate (**P=0.0558**).

Discontinuations due to AE

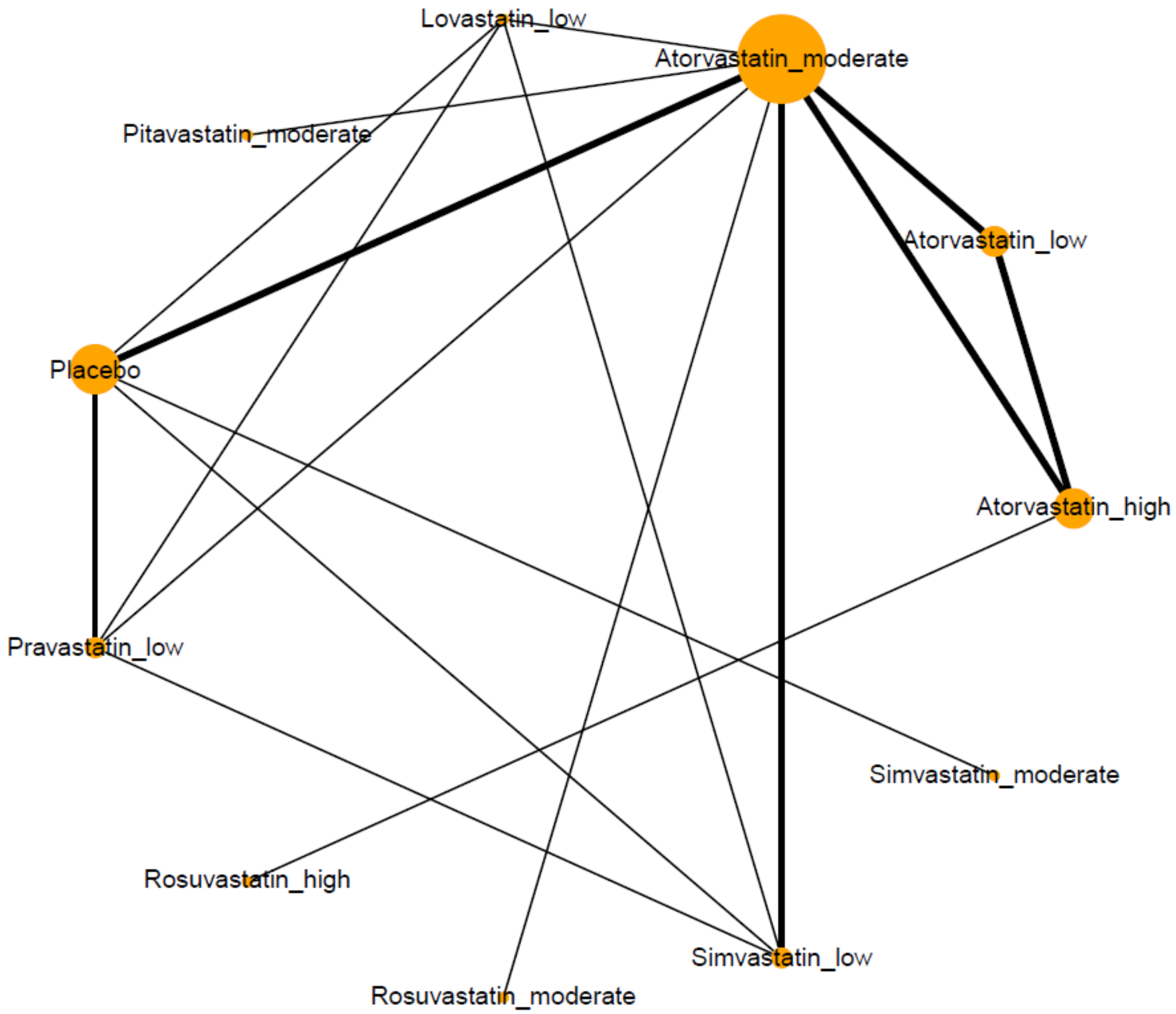

Raw meta-data for discontinuations due to adverse events

Study	Statin Intensity	Number of Discontinuations	Total Number of Patients
Behounek 1994	Pravastatin_low	2	167
Behounek 1994	Placebo	0	158
Betteridge 2007	Rosuvastatin_moderate	15	254
Betteridge 2007	Atorvastatin_moderate	13	255
Chang 2013	Atorvastatin_low	3	52
Chang 2013	Atorvastatin_moderate	2	52
Chang 2013	Atorvastatin_high	2	53
Colhoun 2004	Atorvastatin_moderate	122	1428
Colhoun 2004	Placebo	145	1410
Ferrer-García 2008	Atorvastatin_high	1	17
Ferrer-García 2008	Atorvastatin_moderate	1	61
Ferrer-García 2008	Atorvastatin_low	0	75
Gentile 2000	Atorvastatin_moderate	1	84
Gentile 2000	Pravastatin_low	1	81
Gentile 2000	Lovastatin_low	1	80
Gentile 2000	Simvastatin_low	0	78
Gentile 2000	Placebo	0	86
Goldberg 2006	Simvastatin_low	4	494
Goldberg 2006	Atorvastatin_moderate	11	735
Knopp 2006	Atorvastatin_moderate	33	1211
Knopp 2006	Placebo	38	1199
Lewin 2004	Simvastatin_moderate	10	123
Lewin 2004	Placebo	4	130
Liu 2013	Pitavastatin_moderate	9	112
Liu 2013	Atorvastatin_moderate	4	113
Son 2013	Atorvastatin_low	2	185
Son 2013	Atorvastatin_moderate	2	181
Son 2013	Atorvastatin_high	1	74
Wolffenbuttel 2005	Rosuvastatin_high	10	131

Wolffenbuttel 2005	Atorvastatin_high	11	132

Given there were only 12 studies that provided data on discontinuations and due to the rare event nature of the outcome, their is no such R/Stata package that are able to extend to the Peto odds ratio. Thus, we opted to analyse discontinuations using traditional pairwise meta-analysis approach and the results can be seen below.

Discontinuations due to AEs were analysed with Peto odds ratio

Study	Experimental Events	Experimental Total	Control Events	Control Total	OR	95%-CI
**Pravastatin_low**						
Behounek1994	2	167	0	158	7.04	[0.44; 113.22]
Gentile2000	1	81	0	86	7.86	[0.16; 396.79]
**Common effect model**		**248**		**244**	**7.31**	**[0.76; 70.47]**
**Random effects model**					**7.31**	**[0.76; 70.47]**
Heterogeneity: $I^2 = 0\%$, $\tau^2 = 0$						
**Atorvastatin_moderate**						
Colhoun2004	122	1428	145	1410	0.82	[0.63; 1.05]
Gentile2000	1	84	0	86	7.57	[0.15; 381.46]
Knopp2006	33	1211	38	1199	0.86	[0.53; 1.37]
**Common effect model**		**2723**		**2695**	**0.83**	**[0.66; 1.04]**
**Random effects model**					**0.83**	**[0.66; 1.04]**
Heterogeneity: $I^2 = 0\%$ [0%; 90%], $\tau^2 = < 0.0001$ [0.0000; 61.2733]						
**Lovastatin_low**						
Gentile2000	1	80	0	86	7.96	[0.16; 402.42]
**Common effect model**		**80**		**86**	**7.96**	**[0.16; 402.42]**
**Random effects model**					**7.96**	**[0.16; 402.42]**
Heterogeneity: not applicable						
**Simvastatin_moderate**						
Lewin2004	10	123	4	130	2.62	[0.89; 7.68]
**Common effect model**		**123**		**130**	**2.62**	**[0.89; 7.68]**
**Random effects model**					**2.62**	**[0.89; 7.68]**
Heterogeneity: not applicable						

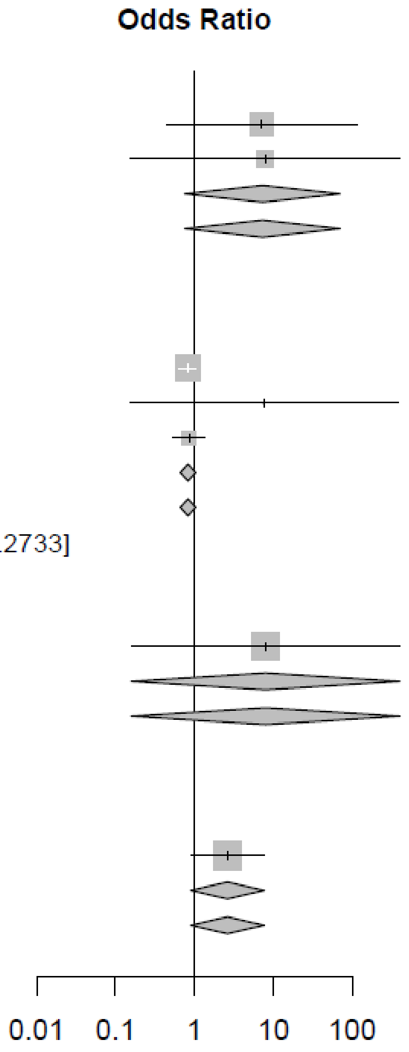

MACE outcomes

Non-Fatal Myocardial Faction

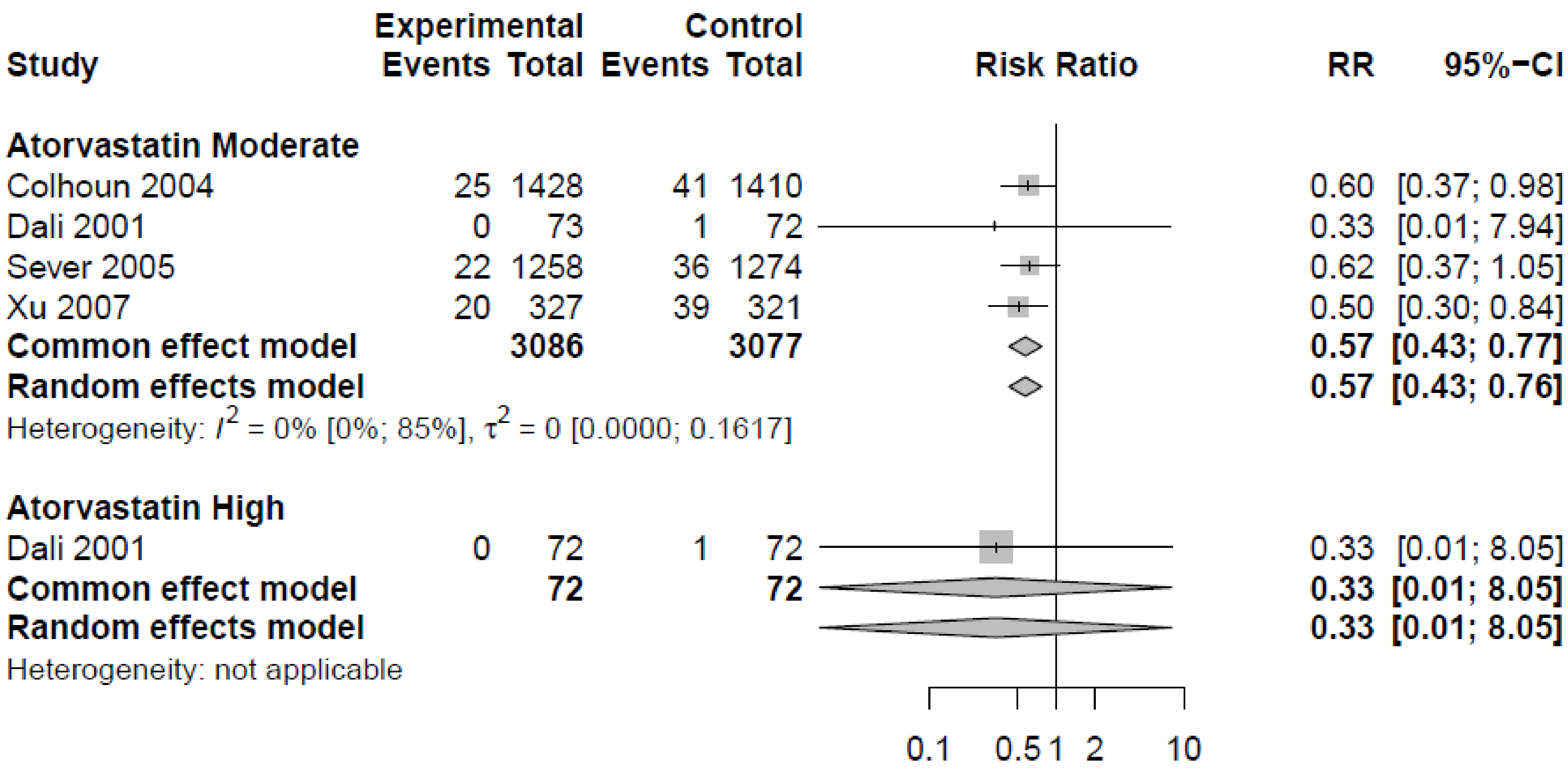

Non-Fatal Stroke

Study	Experimental Events	Experimental Total	Control Events	Control Total	RR	95%−CI
**Atorvastatin Moderate**						
Colhoun 2004	20	1428	30	1410	0.66	[0.38; 1.15]
Sever 2005	23	1258	31	1274	0.75	[0.44; 1.28]
**Common effect model**		**2686**		**2684**	**0.71**	**[0.48; 1.04]**
**Random effects model**					**0.71**	**[0.48; 1.04]**

Heterogeneity: $I^2 = 0\%$, $\tau^2 = 0$

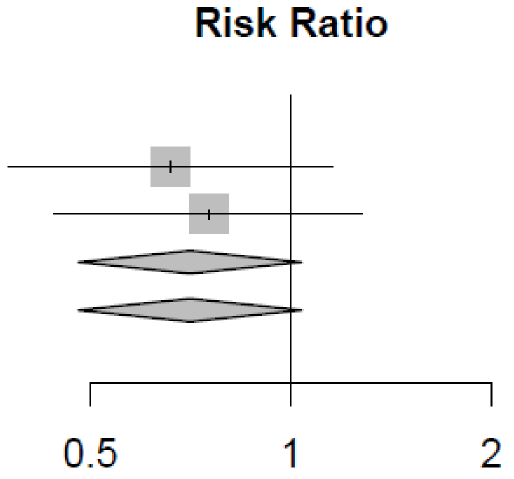

Cardiovascular-related death

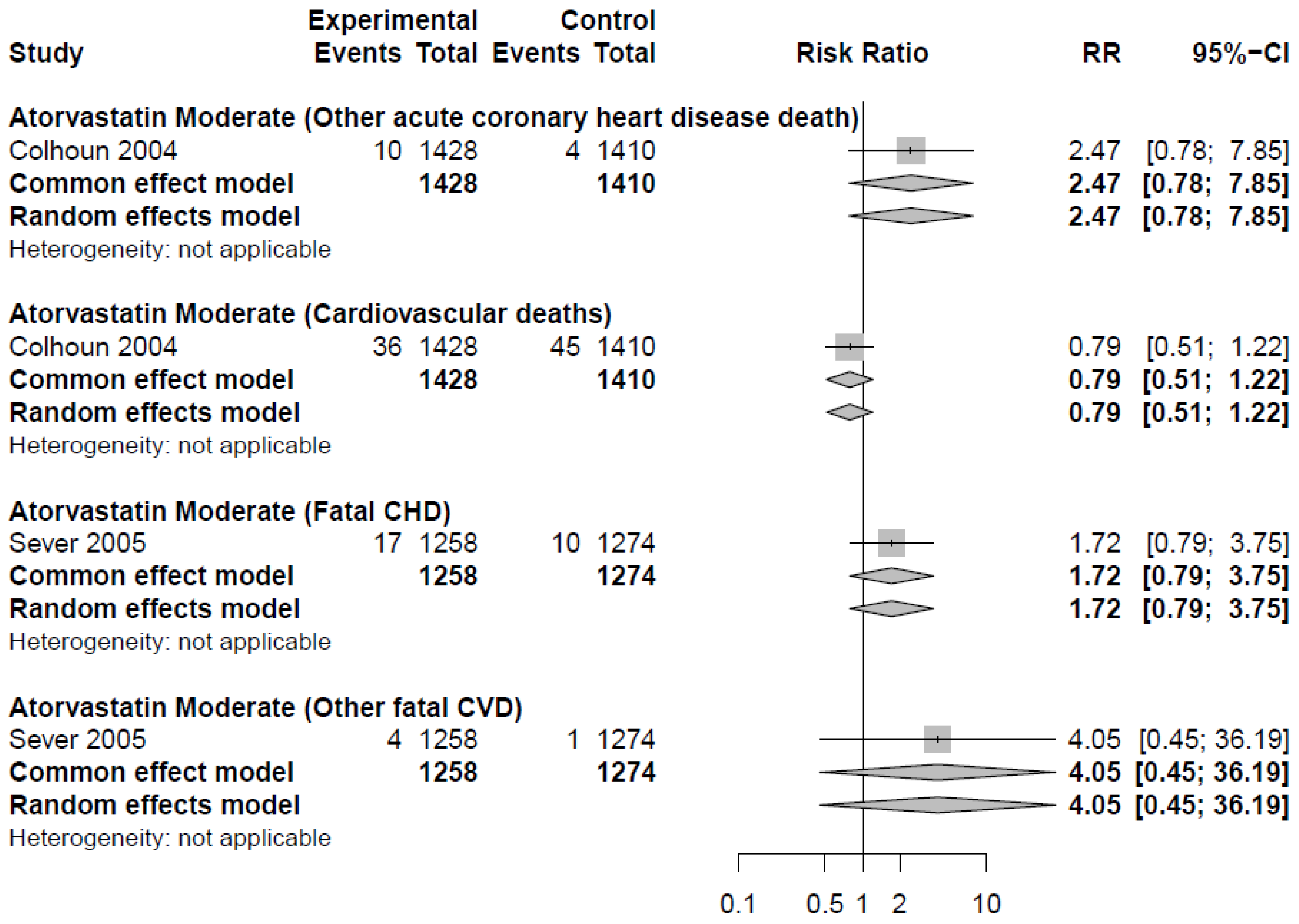

Appendix 10: Treatment Ranking using SUCRA

Main intensity model (non-HDL-C):

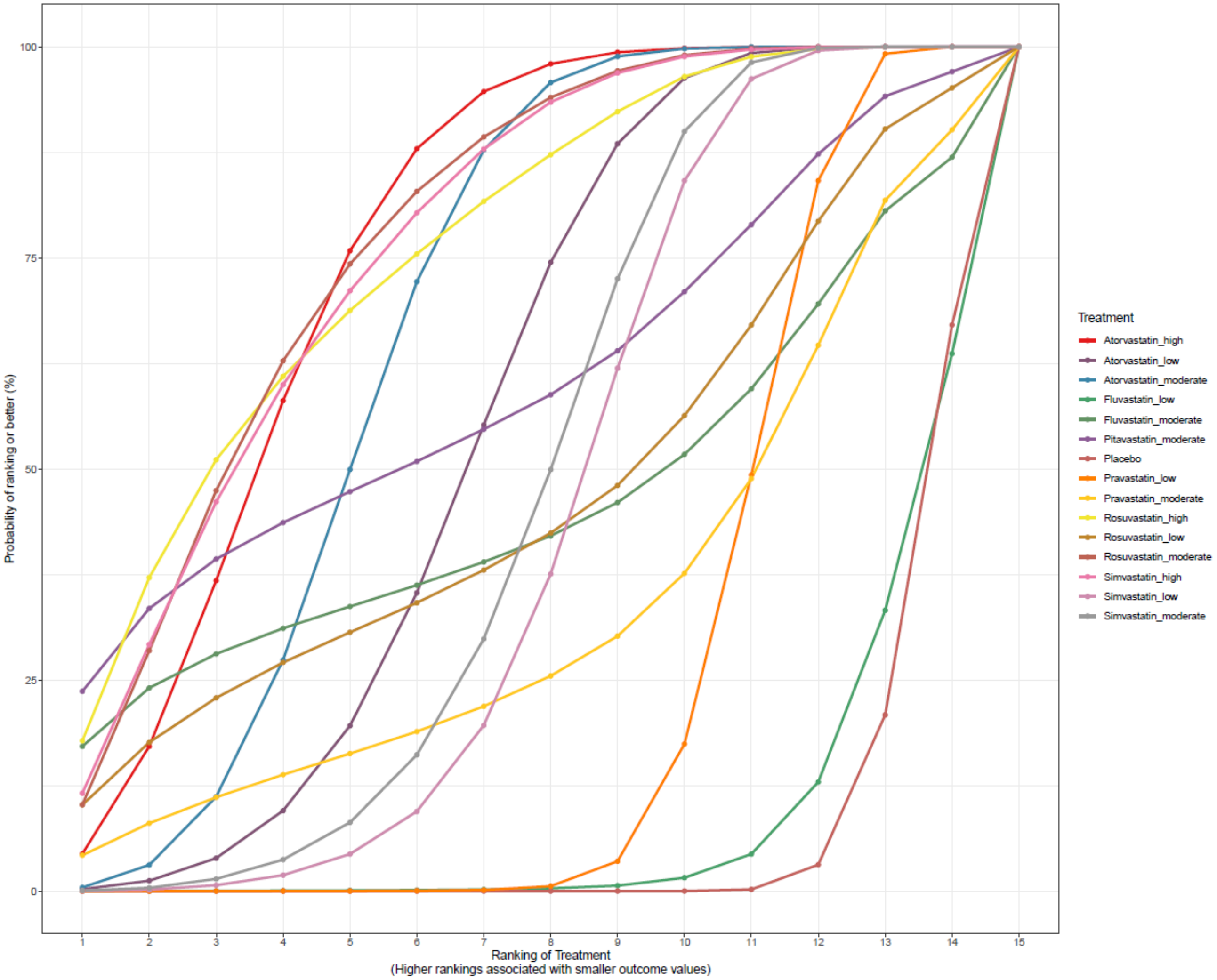

**Statin Agent and Intensity**	**SUCRA score***
Rosuvastatin moderate	0.77496696
Rosuvastatin high	0.76798482
Simvastatin high	0.7667625
Atorvastatin high	0.76289821
Atorvastatin moderate	0.67539286
Pitavastatin moderate	0.60872321
Atorvastatin low	0.55788661
Simvastatin moderate	0.47678304
Rosuvastatin low	0.47610179
Fluvastatin moderate	0.44762411
Simvastatin low	0.43856875
Pravastatin moderate	0.34252589
Pravastatin low	0.25295982
Fluvastatin low	0.08495804
Placebo	0.06586339

*Larger SUCRAs (green) denote the more effective statin agents and intensities.

Patient Risk Adjusted Model (non-HDL-C):

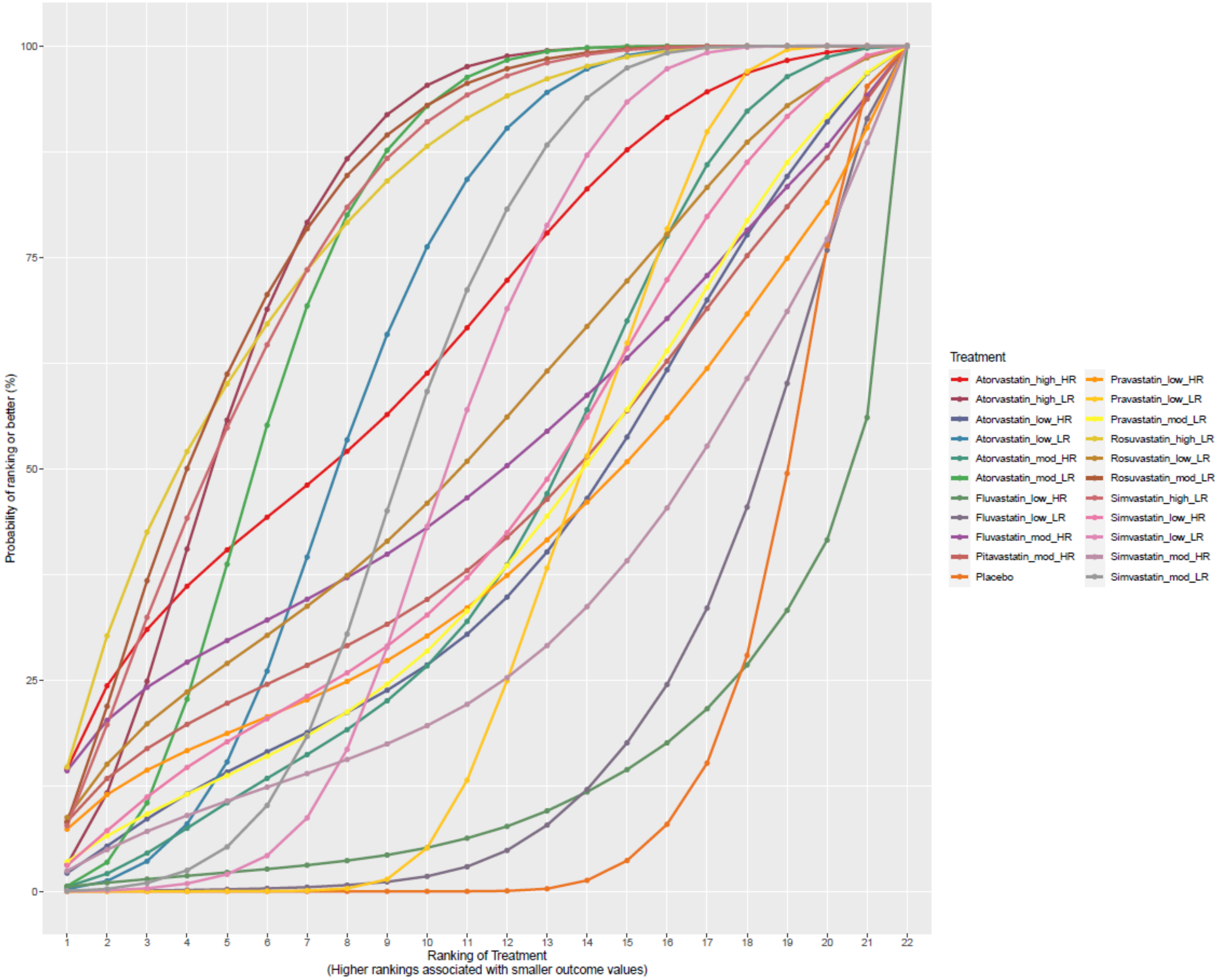

**Statin Agent and Intensity**	**SUCRA score***
Rosuvastatin mod LR	0.803122
Rosuvastatin high LR	0.7935393
Atorvastatin high LR	0.7892732
Simvastatin high LR	0.7831512
Atorvastatin mod LR	0.74255
Atorvastatin low LR	0.6465827
Atorvastatin high HR	0.6400351
Simvastatin mod LR	0.5725964
Rosuvastatin low LR	0.5389274
Simvastatin low LR	0.5183702
Fluvastatin mod HR	0.5161702
Simvastatin low HR	0.4550923
Pitavastatin mod HR	0.4390083
Atorvastatin mod HR	0.4355357
Pravastatin mod LR	0.4157256
Pravastatin low HR	0.3981042
Atorvastatin low HR	0.3963363
Pravastatin low LR	0.3646827
Simvastatin mod HR	0.3133452
Fluvastatin low LR	0.1828077
Placebo	0.1323952
Fluvastatin low HR	0.1226488

Main intensity model (LDL-C):

Statin Agent and Intensity	SUCRA score*
Simvastatin_high	0.931794
Rosuvastatin_high	0.8928002
Rosuvastatin_moderate	0.7886903
Atorvastatin_high	0.7421468
Simvastatin_low	0.6394138
Atorvastatin_moderate	0.5972458
Rosuvastatin_low	0.5796707
Pitavastatin_moderate	0.5468163
Lovastatin_low	0.451416
Atorvastatin_low	0.4354168
Simvastatin_moderate	0.4109872
Fluvastatin_moderate	0.368182
Pravastatin_moderate	0.2859857
Pravastatin_low	0.2496543
Placebo	0.0548915
Fluvastatin_low	0.0248885

Appendix 11: CINeMA Assessment

Using the CINeMA framework, we evaluated the certainty of evidence for each network estimate according to the following criteria:

- **Within study bias:** We categorized the overall risk of bias of each study as low risk of bias when none of the four assessed risk of bias item was rated high risk, as moderate risk of bias when one or two items were rated high risk, and as high risk when three or four were rated high risk. We then used the contribution matrix to calculate the percentage of contribution from each study, and finally assessed the study limitation for each network estimate based on the weighted average risk of bias of the contributing studies (Appendix 5).
- **Reporting bias:** We spotted major concerns visually with the comparison adjusted funnel plot to see if any statistically significance was present in pairwise comparisons (Appendix 11).
- **Indirectness:** Transitivity assumption was assessed through mean TC and HDL-C baseline scores for those reported and mean/SD age by comparing baseline consistency across intervention groups (See after CINeMA table).
- **Imprecision:** We considered a clinically meaningful threshold for SMD to be 0.20 and graded the degree of concerns according to the possible changes in clinical inference according to the following criteria:

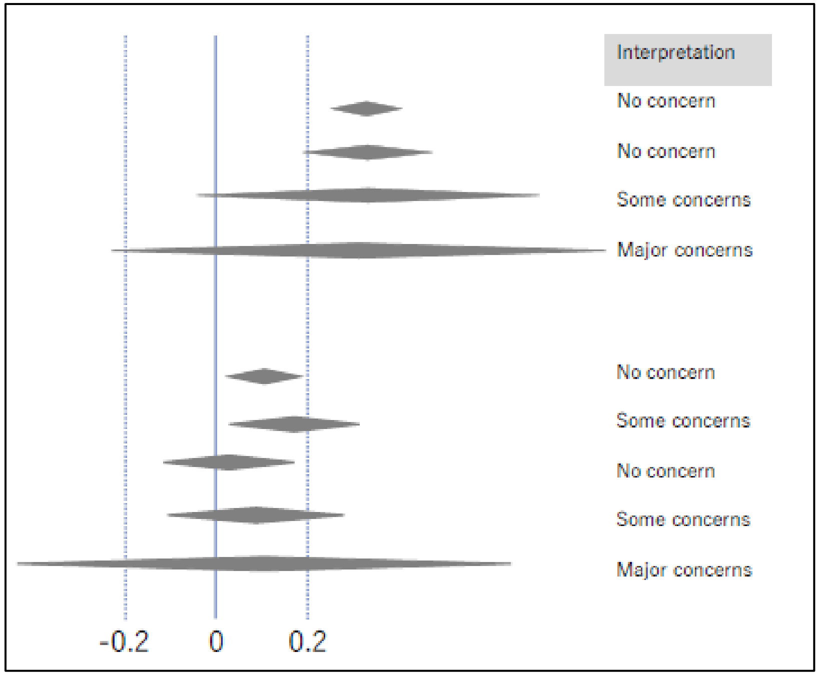

- **Heterogeneity:** We evaluated the degree of concerns through comparing the clinical inference based on the 95% credible intervals (CrIs) whilst applying the same clinical inference framework as for imprecision. To support this heterogeneity assessment the Analysis of heterogeneity (ANOHE) function (‘mtc.anohe’) in the GeMTC package in R was used to assess I^2 simultaneously whilst comparing pairwise contributions and network contributions. The function attempts to draw a distinction between inconsistency and heterogeneity but it should only be considered experimental (van Valkenhoef et al. 2014). Nevertheless, it can still be a useful guide for assessing heterogeneity in the network.

- **Incoherence (Inconsistency):** For inconsistency, we looked at the results of node-splitting (Appendix 7) and we saw major concerns when $p<0.10$ but no concern otherwise.

CINeMA Results Table

Comparison	No. of studies	Within-study bias	Reporting bias	Indirectness	Imprecision	Heterogeneity	Incoherence	Confidence rating
**Mixed evidence**								
Atorvastatin_high vs. Atorvastatin_low	4	Major concerns	Undetected	No concerns	No concerns	Some concerns	No concerns	Low
Atorvastatin_high vs. Atorvastatin_moderate	6	Major concerns	Undetected	No concerns	No concerns	No concerns	No concerns	Moderate
Atorvastatin_high vs. Placebo	4	Major concerns	Undetected	No concerns	No concerns	No concerns	No concerns	Moderate
Atorvastatin_high vs. Rosuvastatin_high	1	Some concerns	Undetected	No concerns	No concerns	No concerns	No concerns	Moderate
Atorvastatin_high vs. Simvastatin_moderate	1	Major concerns	Suspected	No concerns	No concerns	No concerns	No concerns	Low
Atorvastatin_low vs. Atorvastatin_moderate	5	Major concerns	Undetected	No concerns	No concerns	No concerns	No concerns	Moderate
Atorvastatin_low vs. Pravastatin_low	1	Some concerns	Undetected	No concerns	No concerns	No concerns	No concerns	Moderate
Atorvastatin_low vs. Rosuvastatin_high	1	Some concerns	Undetected	No concerns	No concerns	No concerns	No concerns	Moderate
Atorvastatin_low vs. Simvastatin_low	2	Major concerns	Undetected	No concerns	No concerns	No concerns	No concerns	Moderate
Atorvastatin_low vs. Simvastatin_moderate	2	Some concerns	Undetected	No concerns	No concerns	No concerns	No concerns	Moderate
Atorvastatin_moderate vs. Pitavastatin_moderate	1	No concerns	Undetected	No concerns	No concerns	No concerns	No concerns	High
Atorvastatin_moderate vs. Placebo	5	No concerns	Undetected	No concerns	No concerns	No concerns	No concerns	High
Atorvastatin_moderate vs. Pravastatin_low	3	Some concerns	Undetected	No concerns	No concerns	No concerns	Major concerns	Low
Atorvastatin_moderate vs. Rosuvastatin_high	1	Some concerns	Some concerns	No concerns	No concerns	No concerns	No concerns	Moderate
Atorvastatin_moderate vs. Rosuvastatin_moderate	1	Some concerns	Undetected	No concerns	No concerns	No concerns	No concerns	Moderate
Atorvastatin_moderate vs. Simvastatin_low	2	Major concerns	Undetected	No concerns	No concerns	No concerns	No concerns	Moderate
Atorvastatin_moderate vs. Simvastatin_moderate	3	Some concerns	Undetected	No concerns	No concerns	No concerns	No concerns	Moderate
Fluvastatin_low vs. Placebo	2	Major concerns	Undetected	No concerns	No concerns	No concerns	No concerns	Moderate
Fluvastatin_moderate vs. Placebo	1	No concerns	Undetected	No concerns	Major concerns	No concerns	No concerns	Moderate

Placebo vs. Pravastatin_low	6	Major concerns	Undetected	Some concerns	No concerns	Some concerns	No concerns	Low
Placebo vs. Pravastatin_moderate	1	No concerns	Undetected	No concerns	Some concerns	No concerns	No concerns	Moderate
Placebo vs. Rosuvastatin_high	1	No concerns	Undetected	No concerns	No concerns	No concerns	No concerns	High
Placebo vs. Rosuvastatin_low	1	No concerns	Undetected	No concerns	Some concerns	No concerns	No concerns	Moderate
Placebo vs. Rosuvastatin_moderate	1	No concerns	Undetected	No concerns	No concerns	No concerns	No concerns	High
Placebo vs. Simvastatin_high	1	No concerns	Undetected	No concerns	No concerns	No concerns	No concerns	High
Placebo vs. Simvastatin_low	2	No concerns	Undetected	No concerns	No concerns	Some concerns	Some concerns	Moderate
Placebo vs. Simvastatin_moderate	6	Some concerns	Undetected	Some concerns	No concerns	No concerns	No concerns	Moderate
Pravastatin_low vs. Pravastatin_moderate	1	No concerns	Undetected	No concerns	No concerns	No concerns	No concerns	High
Pravastatin_low vs. Rosuvastatin_high	1	Some concerns	Undetected	No concerns	No concerns	No concerns	No concerns	Moderate
Pravastatin_low vs. Rosuvastatin_moderate	1	Some concerns	Undetected	No concerns	No concerns	No concerns	No concerns	Moderate
Pravastatin_low vs. Simvastatin_low	1	Major concerns	Undetected	No concerns	No concerns	No concerns	No concerns	Moderate
Pravastatin_low vs. Simvastatin_moderate	2	Some concerns	Undetected	No concerns	No concerns	Major concerns	Major concerns	Very Low
Rosuvastatin_high vs. Rosuvastatin_low	1	No concerns	Suspected	No concerns	No concerns	No concerns	No concerns	Moderate
Rosuvastatin_high vs. Rosuvastatin_moderate	1	No concerns	Suspected	No concerns	No concerns	No concerns	No concerns	Moderate
Rosuvastatin_high vs. Simvastatin_moderate	1	Some concerns	Suspected	No concerns	No concerns	No concerns	No concerns	Low
Rosuvastatin_low vs. Rosuvastatin_moderate	1	No concerns	Undetected	No concerns	No concerns	No concerns	No concerns	High
Simvastatin_high vs. Simvastatin_low	1	No concerns	Undetected	No concerns	No concerns	No concerns	No concerns	High
Simvastatin_high vs. Simvastatin_moderate	2	No concerns	Undetected	No concerns	No concerns	No concerns	No concerns	High
Simvastatin_low vs. Simvastatin_moderate	1	No concerns	Undetected	No concerns	No concerns	No concerns	No concerns	High
Indirect evidence								
Atorvastatin_high vs. Fluvastatin_low	0	Major concerns	Undetected	No concerns	No concerns	No concerns	No concerns	Moderate

Atorvastatin_high vs. Fluvastatin_moderate	0	No concerns	Undetected	No concerns	No concerns	No concerns	No concerns	High
Atorvastatin_high vs. Pitavastatin_moderate	0	No concerns	Undetected	No concerns	No concerns	No concerns	No concerns	High
Atorvastatin_high vs. Pravastatin_low	0	Some concerns	Undetected	No concerns	No concerns	No concerns	No concerns	Moderate
Atorvastatin_high vs. Pravastatin_moderate	0	No concerns	Undetected	No concerns	Some concerns	No concerns	No concerns	Moderate
Atorvastatin_high vs. Rosuvastatin_low	0	No concerns	Undetected	No concerns	No concerns	No concerns	No concerns	High
Atorvastatin_high vs. Rosuvastatin_moderate	0	Some concerns	Undetected	No concerns	No concerns	No concerns	No concerns	Moderate
Atorvastatin_high vs. Simvastatin_high	0	No concerns	Undetected	No concerns	No concerns	No concerns	No concerns	High
Atorvastatin_high vs. Simvastatin_low	0	Major concerns	Undetected	No concerns	No concerns	No concerns	No concerns	Moderate
Atorvastatin_low vs. Fluvastatin_low	0	Major concerns	Undetected	No concerns	No concerns	No concerns	No concerns	Moderate
Atorvastatin_low vs. Fluvastatin_moderate	0	No concerns	Undetected	No concerns	No concerns	No concerns	No concerns	High
Atorvastatin_low vs. Pitavastatin_moderate	0	No concerns	Undetected	No concerns	No concerns	No concerns	No concerns	High
Atorvastatin_low vs. Placebo	0	Major concerns	Undetected	No concerns	No concerns	No concerns	No concerns	Moderate
Atorvastatin_low vs. Pravastatin_moderate	0	No concerns	Undetected	No concerns	No concerns	No concerns	No concerns	High
Atorvastatin_low vs. Rosuvastatin_low	0	No concerns	Undetected	No concerns	No concerns	No concerns	No concerns	High
Atorvastatin_low vs. Rosuvastatin_moderate	0	Some concerns	Undetected	No concerns	No concerns	No concerns	No concerns	Moderate
Atorvastatin_low vs. Simvastatin_high	0	No concerns	Undetected	No concerns	No concerns	No concerns	No concerns	High
Atorvastatin_moderate vs. Fluvastatin_low	0	No concerns	Undetected	No concerns	No concerns	No concerns	No concerns	High
Atorvastatin_moderate vs. Fluvastatin_moderate	0	No concerns	Undetected	No concerns	No concerns	No concerns	No concerns	High
Atorvastatin_moderate vs. Pravastatin_moderate	0	No concerns	Undetected	No concerns	No concerns	No concerns	No concerns	High
Atorvastatin_moderate vs. Rosuvastatin_low	0	No concerns	Undetected	No concerns	No concerns	No concerns	No concerns	High

Atorvastatin_moderate vs. Simvastatin_high	0	No concerns	Undetected	No concerns	No concerns	No concerns	No concerns	High
Fluvastatin_low vs. Fluvastatin_moderate	0	No concerns	Undetected	No concerns	Major concerns	No concerns	No concerns	Moderate
Fluvastatin_low vs. Pitavastatin_moderate	0	No concerns	Undetected	No concerns	No concerns	No concerns	No concerns	High
Fluvastatin_low vs. Pravastatin_low	0	Major concerns	Undetected	No concerns	No concerns	No concerns	No concerns	Moderate
Fluvastatin_low vs. Pravastatin_moderate	0	No concerns	Undetected	No concerns	Major concerns	No concerns	No concerns	Moderate
Fluvastatin_low vs. Rosuvastatin_high	0	No concerns	Undetected	No concerns	No concerns	No concerns	No concerns	High
Fluvastatin_low vs. Rosuvastatin_low	0	No concerns	Undetected	No concerns	Some concerns	No concerns	No concerns	Moderate
Fluvastatin_low vs. Rosuvastatin_moderate	0	No concerns	Undetected	No concerns	No concerns	No concerns	No concerns	High
Fluvastatin_low vs. Simvastatin_high	0	No concerns	Undetected	No concerns	No concerns	No concerns	No concerns	High
Fluvastatin_low vs. Simvastatin_low	0	No concerns	Undetected	No concerns	No concerns	No concerns	No concerns	High
Fluvastatin_low vs. Simvastatin_moderate	0	No concerns	Undetected	No concerns	No concerns	No concerns	No concerns	High
Fluvastatin_moderate vs. Pitavastatin_moderate	0	No concerns	Undetected	No concerns	No concerns	No concerns	No concerns	High
Fluvastatin_moderate vs. Pravastatin_low	0	No concerns	Undetected	No concerns	No concerns	No concerns	No concerns	High
Fluvastatin_moderate vs. Pravastatin_moderate	0	No concerns	Undetected	No concerns	No concerns	No concerns	No concerns	High
Fluvastatin_moderate vs. Rosuvastatin_high	0	No concerns	Undetected	No concerns	No concerns	No concerns	No concerns	High
Fluvastatin_moderate vs. Rosuvastatin_low	0	No concerns	Undetected	No concerns	No concerns	No concerns	No concerns	High
Fluvastatin_moderate vs. Rosuvastatin_moderate	0	No concerns	Undetected	No concerns	No concerns	No concerns	No concerns	High
Fluvastatin_moderate vs. Simvastatin_high	0	No concerns	Undetected	No concerns	No concerns	No concerns	No concerns	High
Fluvastatin_moderate vs. Simvastatin_low	0	No concerns	Undetected	No concerns	No concerns	No concerns	No concerns	High
Fluvastatin_moderate vs. Simvastatin_moderate	0	No concerns	Undetected	No concerns	No concerns	No concerns	No concerns	High

Pitavastatin_moderate vs. Placebo	0	No concerns	Undetected	No concerns	Major concerns	No concerns	No concerns	Moderate
Pitavastatin_moderate vs. Pravastatin_low	0	No concerns	Undetected	No concerns	No concerns	No concerns	No concerns	High
Pitavastatin_moderate vs. Pravastatin_moderate	0	No concerns	Undetected	No concerns	No concerns	No concerns	No concerns	High
Pitavastatin_moderate vs. Rosuvastatin_high	0	No concerns	Undetected	No concerns	Some concerns	No concerns	No concerns	Moderate
Pitavastatin_moderate vs. Rosuvastatin_low	0	No concerns	Undetected	No concerns	No concerns	No concerns	No concerns	High
Pitavastatin_moderate vs. Rosuvastatin_moderate	0	No concerns	Undetected	No concerns	Some concerns	No concerns	No concerns	Moderate
Pitavastatin_moderate vs. Simvastatin_high	0	No concerns	Undetected	No concerns	Some concerns	No concerns	No concerns	Moderate
Pitavastatin_moderate vs. Simvastatin_low	0	No concerns	Undetected	No concerns	No concerns	No concerns	No concerns	High
Pitavastatin_moderate vs. Simvastatin_moderate	0	No concerns	Undetected	No concerns	No concerns	No concerns	No concerns	High
Pravastatin_low vs. Rosuvastatin_low	0	No concerns	Undetected	No concerns	No concerns	No concerns	No concerns	High
Pravastatin_low vs. Simvastatin_high	0	No concerns	Undetected	No concerns	No concerns	No concerns	No concerns	High
Pravastatin_moderate vs. Rosuvastatin_high	0	No concerns	Undetected	No concerns	No concerns	No concerns	No concerns	High
Pravastatin_moderate vs. Rosuvastatin_low	0	No concerns	Undetected	No concerns	No concerns	No concerns	No concerns	High
Pravastatin_moderate vs. Rosuvastatin_moderate	0	No concerns	Undetected	No concerns	No concerns	No concerns	No concerns	High
Pravastatin_moderate vs. Simvastatin_high	0	No concerns	Undetected	No concerns	No concerns	No concerns	No concerns	High
Pravastatin_moderate vs. Simvastatin_low	0	No concerns	Undetected	No concerns	No concerns	No concerns	No concerns	High
Pravastatin_moderate vs. Simvastatin_moderate	0	No concerns	Undetected	No concerns	No concerns	No concerns	No concerns	High
Rosuvastatin_high vs. Simvastatin_high	0	No concerns	Undetected	No concerns	No concerns	No concerns	No concerns	High
Rosuvastatin_high vs. Simvastatin_low	0	Some concerns	Undetected	No concerns	No concerns	No concerns	No concerns	Moderate

Rosuvastatin_low vs. Simvastatin_high	0	No concerns	Undetected	No concerns	No concerns	No concerns	No concerns	High
Rosuvastatin_low vs. Simvastatin_low	0	No concerns	Undetected	No concerns	No concerns	No concerns	No concerns	High
Rosuvastatin_low vs. Simvastatin_moderate	0	No concerns	Undetected	No concerns	No concerns	No concerns	No concerns	High
Rosuvastatin_moderate vs. Simvastatin_high	0	No concerns	Undetected	No concerns	No concerns	No concerns	No concerns	High
Rosuvastatin_moderate vs. Simvastatin_low	0	No concerns	Undetected	No concerns	No concerns	No concerns	No concerns	High
Rosuvastatin_moderate vs. Simvastatin_moderate	0	Some concerns	Undetected	No concerns	No concerns	No concerns	No concerns	Moderate

Transitivity (Indirectness) Assessment:

Pooled baseline characteristics for each statin intervention and placebo

	Baseline variable (Mean ± SD)		
**Statin intervention**	*Age (yrs.)*	*Total Cholesterol (mmol/L)*	*HDL-C (mmol/L)*
Atorvastatin_high	59.20 ± 9.46	5.95 ± 4.66	1.21 ± 1.75
Atorvastatin_low	61.37 ± 9.72	7.91 ± 3.36	0.62 ± 0.25
Atorvastatin_moderate	60.16 ± 8.77	5.84 ± 14.06	1.25 ± 5.09
Fluvastatin_low	62.90 ± 19.00	5.52 ± 2.86	1.24 ± 1.20
Fluvastatin_moderate	68.00 ± 10.00	6.32 ± 6.35	1.14 ± 2.01
Pitavastatin_moderate	58.70 ± 8.60	6.08 ± 7.33	1.42 ± 3.68
Pravastatin_low	54.28 ± 3.03	6.24 ± 4.79	1.32 ± 1.92
Pravastatin_moderate	60.00 ± NR	5.95 ± 5.93	1.42 ± 2.08
Rosuvastatin_high	56.20 ± 7.16	6.11 ± 11.15	1.22 ± 3.35
Rosuvastatin_low	57.00 ± 7.50	NR	NR
Rosuvastatin_moderate	59.75 ± 4.55	6.06 ± 2.97	1.46 ± 1.60
Simvastatin_high	58.70 ± 5.90	6.22 ± 3.10	1.02 ± 0.39
Simvastatin_low	60.44 ± 5.45	6.00 ± 3.14	1.18 ± 1.59
Simvastatin_moderate	55.92 ± 5.89	6.30 ± 5.82	1.17 ± 2.11
Placebo	56.98 ± 8.33	8.96 ± 7.87	1.79 ± 3.89
**Pooled average**	**59.31 ± 8.10**	**6.39 ± 5.96**	**1.25 ± 2.21**

NR: not reported.

Plot of mean age by each intervention group and study

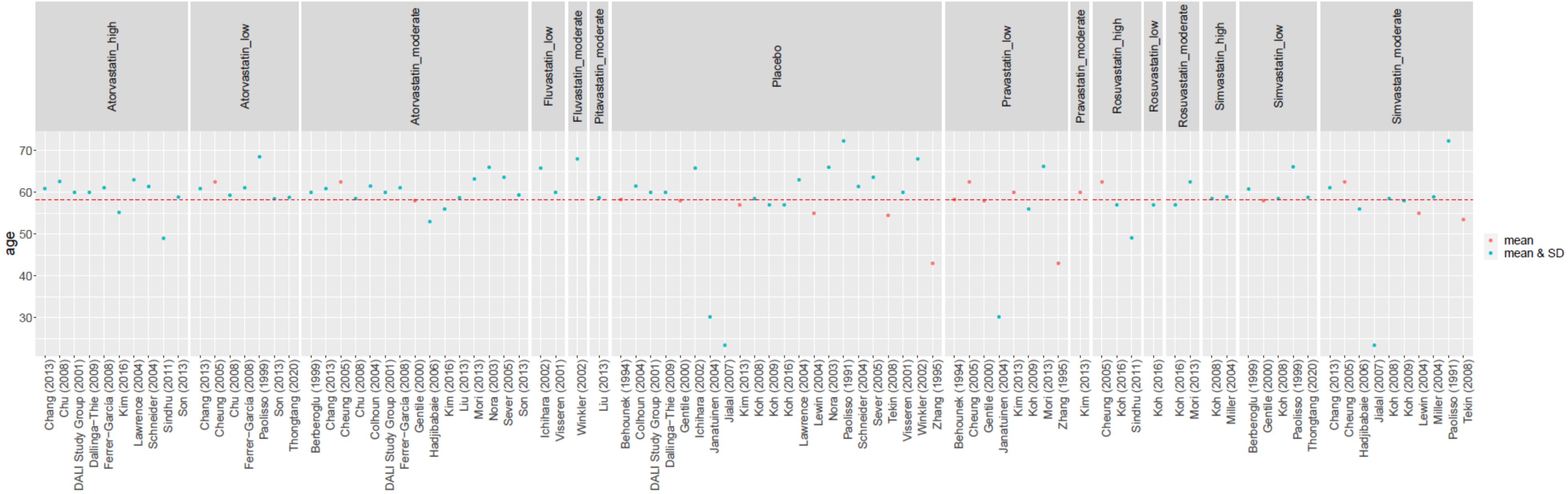

Appendix 12: Comparison-Adjusted Funnel Plots

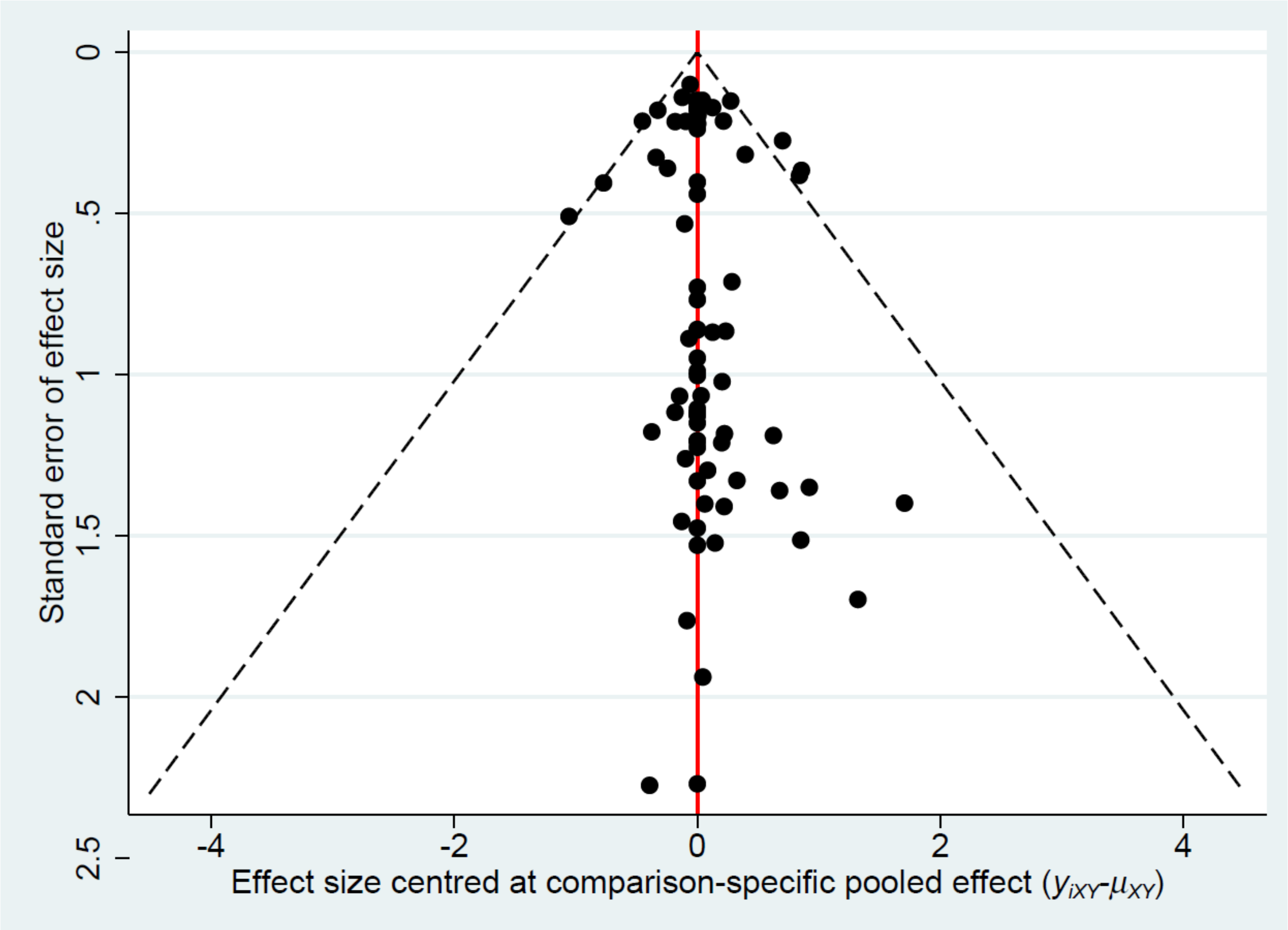

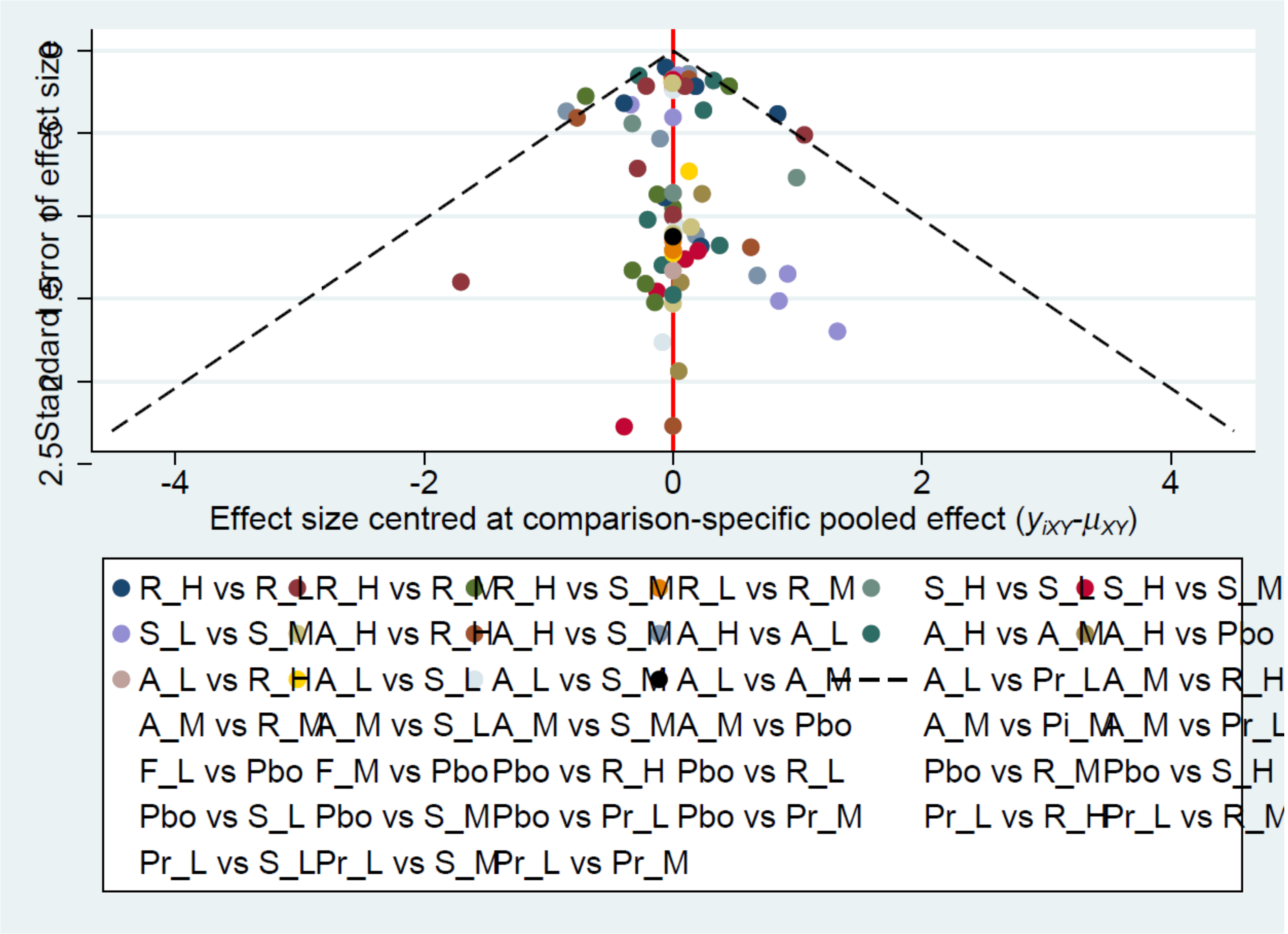

Standard error of effect size
0
.5
1
1.5
2
2.5
-4
-2
0
2
4
Effect size centred at comparison-specific pooled effect (y_{iXY}-μ_{XY})
R_H vs R_L
R_H vs R_M
R_H vs S_M
R_L vs R_M
S_H vs S_L
S_H vs S_M
S_L vs S_M
A_H vs R_H
A_H vs S_M
A_H vs A_L
A_H vs A_M
A_H vs Pbo
A_L vs R_H
A_L vs S_L
A_L vs S_M
A_L vs A_M
A_L vs Pr_L
A_M vs R_H
A_M vs R_M
A_M vs S_L
A_M vs S_M
A_M vs Pbo
A_M vs Pi_M
A_M vs Pr_L
F_L vs Pbo
F_M vs Pbo
Pbo vs R_H
Pbo vs R_L
Pbo vs R_M
Pbo vs S_H
Pbo vs S_L
Pbo vs S_M
Pbo vs Pr_L
Pbo vs Pr_M
Pr_L vs R_H
Pr_L vs R_M
Pr_L vs S_L
Pr_L vs S_M
Pr_L vs Pr_M

Appendix 13: Sensitivity Analysis of Network Model by Dose

Full Dose model:

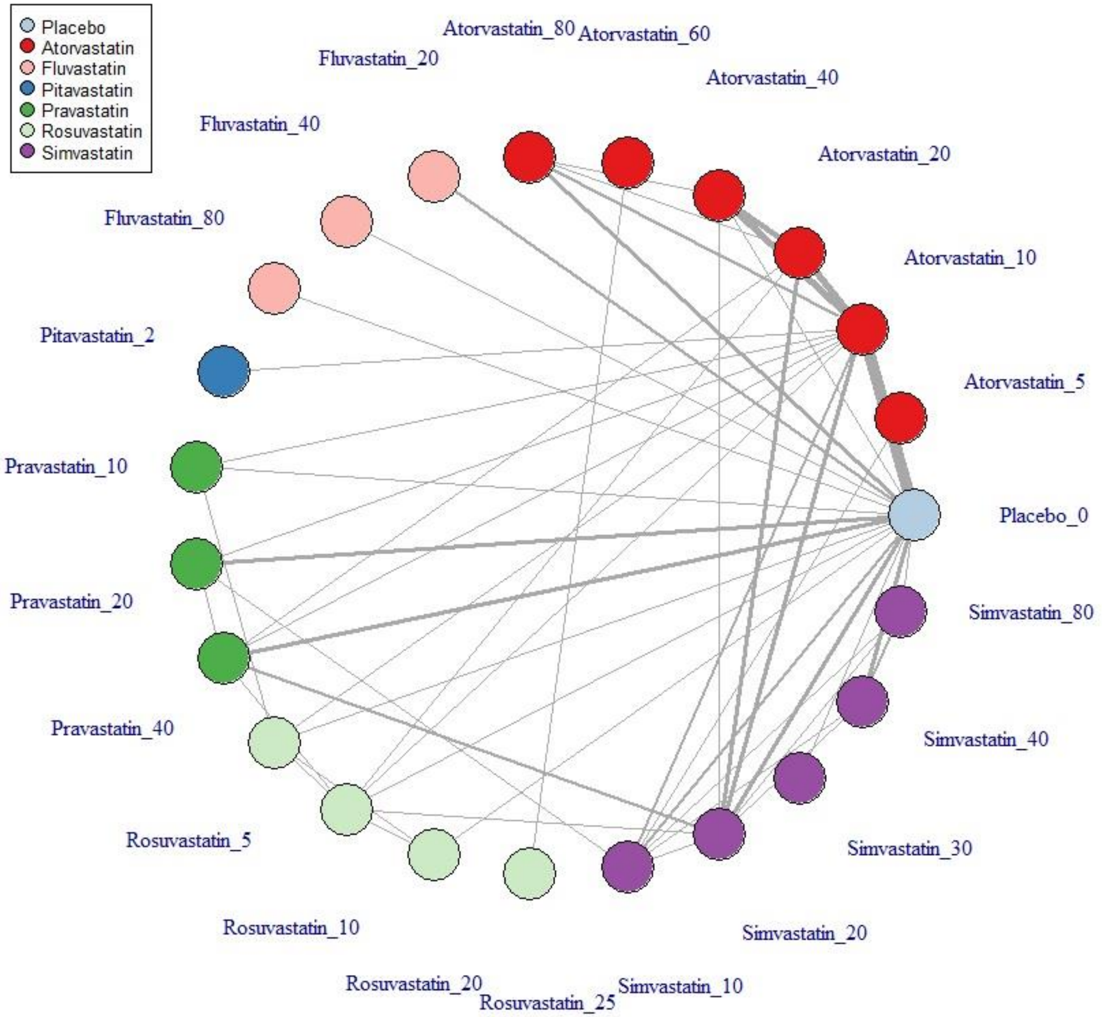

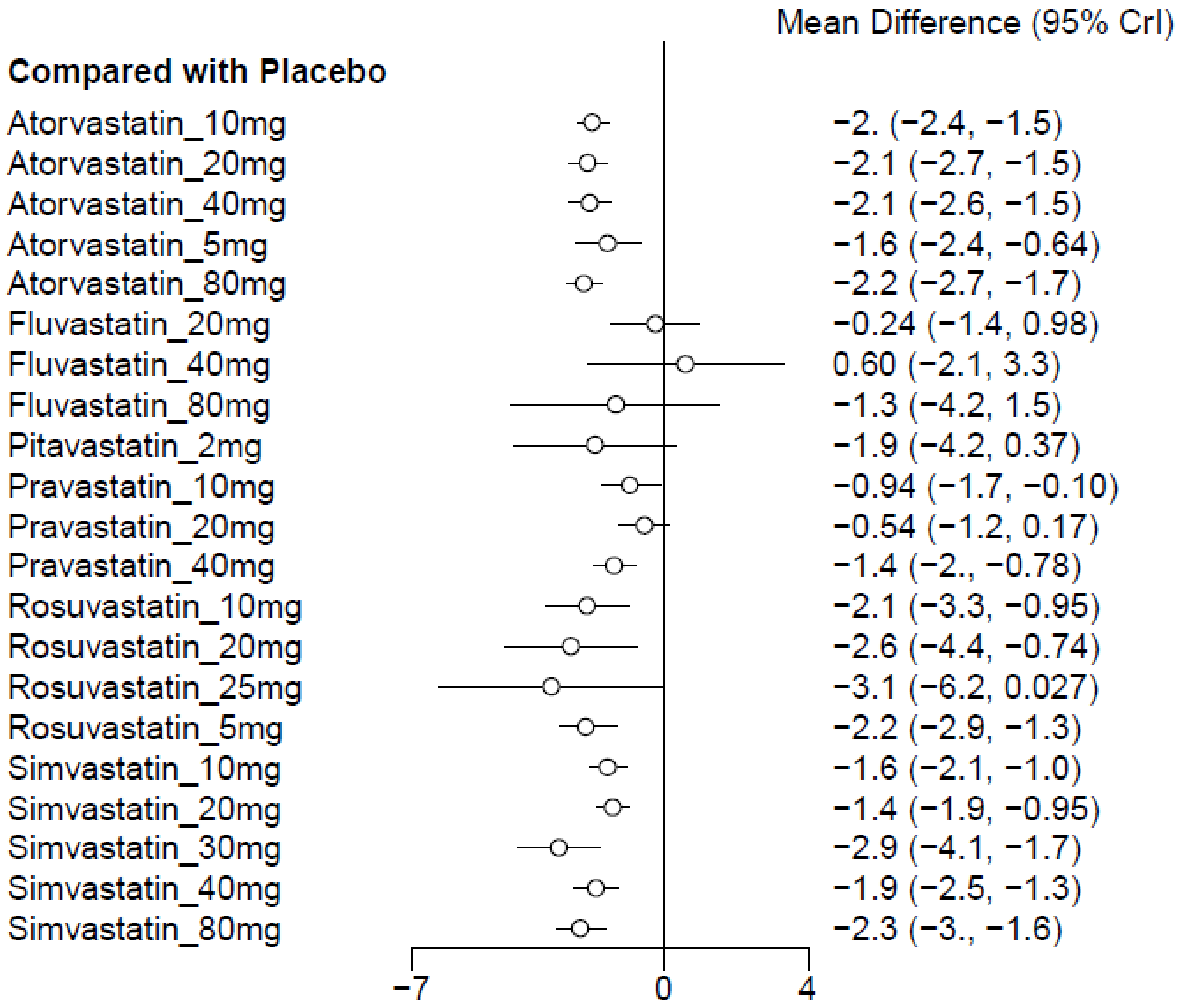
Mean Difference (95% CrI)
Compared with Placebo
Atorvastatin_10mg −2. (−2.4, −1.5)
Atorvastatin_20mg −2.1 (−2.7, −1.5)
Atorvastatin_40mg −2.1 (−2.6, −1.5)
Atorvastatin_5mg −1.6 (−2.4, −0.64)
Atorvastatin_80mg −2.2 (−2.7, −1.7)
Fluvastatin_20mg −0.24 (−1.4, 0.98)
Fluvastatin_40mg 0.60 (−2.1, 3.3)
Fluvastatin_80mg −1.3 (−4.2, 1.5)
Pitavastatin_2mg −1.9 (−4.2, 0.37)
Pravastatin_10mg −0.94 (−1.7, −0.10)
Pravastatin_20mg −0.54 (−1.2, 0.17)
Pravastatin_40mg −1.4 (−2., −0.78)
Rosuvastatin_10mg −2.1 (−3.3, −0.95)
Rosuvastatin_20mg −2.6 (−4.4, −0.74)
Rosuvastatin_25mg −3.1 (−6.2, 0.027)
Rosuvastatin_5mg −2.2 (−2.9, −1.3)
Simvastatin_10mg −1.6 (−2.1, −1.0)
Simvastatin_20mg −1.4 (−1.9, −0.95)
Simvastatin_30mg −2.9 (−4.1, −1.7)
Simvastatin_40mg −1.9 (−2.5, −1.3)
Simvastatin_80mg −2.3 (−3., −1.6)
−7
0
4

SUCRA ranking scores for dose model

Statin dose	SUCRA
Simvastatin_30mg	0.88393369
Rosuvastatin_25mg	0.79916333
Simvastatin_80mg	0.7657019
Rosuvastatin_20mg	0.75476976
Atorvastatin_80mg	0.73942417
Rosuvastatin_5mg	0.69471548
Atorvastatin_20mg	0.6831675
Rosuvastatin_10mg	0.66163071
Atorvastatin_40mg	0.64883845
Atorvastatin_10mg	0.6054731
Pitavastatin_2mg	0.56963798
Simvastatin_40mg	0.56301345
Fluvastatin_80mg	0.44912214
Atorvastatin_5mg	0.42551083
Simvastatin_10mg	0.41047107
Simvastatin_20mg	0.35371524
Pravastatin_40mg	0.34568488
Pravastatin_10mg	0.22700405
Pravastatin_20mg	0.15143417
Fluvastatin_20mg	0.11654107
Fluvastatin_40mg	0.08704774
Placebo	0.06399929

Node-split inconsistency analysis:

Comparison	P-value	CrI
Atorvastatin_10mg vs. Atorvastatin_20mg	0.287	
- direct		-0.035 (-0.53, 0.31)
- indirect		0.72 (-0.64, 2.3)
- network		-0.12 (-0.63, 0.31)
Atorvastatin_10mg vs. Atorvastatin_40mg	0.6705	
- direct		0.015 (-0.57, 0.47)
- indirect		-0.58 (-3.3, 2.4)
- network		-0.057 (-0.60, 0.39)
Atorvastatin_10mg vs. Atorvastatin_80mg	0.69125	
- direct		-0.13 (-0.66, 0.38)
- indirect		-0.48 (-2.1, 1.1)
- network		-0.23 (-0.71, 0.19)
Atorvastatin_10mg vs. Placebo	0.5775	
- direct		2. (1.5, 2.4)
- indirect		1.8 (1.2, 2.4)
- network		2. (1.5, 2.4)
Atorvastatin_10mg vs. Pravastatin_10mg	0.91775	
-> direct		1.0 (0.27, 1.7)
-> indirect		0.84 (-2.4, 5.2)
-> network		1.1 (0.37, 1.7)
Atorvastatin_10mg vs. Pravastatin_20mg	0.25725	
- direct		1.9 (0.83, 3.1)
- indirect		0.76 (-1.1, 2.5)
- network		1.4 (0.68, 2.2)
Atorvastatin_10mg vs. Pravastatin_40mg	0.66775	
- direct		0.17 (-1.8, 2.3)
- indirect		0.66 (-0.083, 1.3)
- network		0.64 (-0.065, 1.3)
Atorvastatin_10mg vs. Rosuvastatin_10mg	0.96975	

- direct		-0.27 (-2.1, 1.5)
- indirect		-0.25 (-2.2, 2.0)
- network		-0.16 (-1.3, 1.0)
Atorvastatin_10mg vs. Rosuvastatin_5mg	0.498	
- direct		-0.23 (-0.94, 0.48)
- indirect		0.52 (-1.6, 2.8)
- network		-0.17 (-0.85, 0.54)
Atorvastatin_10mg vs. Simvastatin_10mg	0.23975	
- direct		0.85 (-0.095, 1.8)
- indirect		0.13 (-0.75, 0.94)
- network		0.43 (-0.17, 0.98)
Atorvastatin_10mg vs. Simvastatin_20mg	0.722	
- direct		0.27 (-1.3, 1.8)
- indirect		0.60 (-0.00082, 1.2)
- network		0.57 (0.013, 1.1)
Atorvastatin_20mg vs. Atorvastatin_40mg	0.38675	
- direct		0.17 (-0.35, 0.66)
- indirect		-0.80 (-2.7, 1.2)
- network		0.066 (-0.49, 0.55)
Atorvastatin_20mg vs. Atorvastatin_80mg	0.3115	
- direct		0.10 (-0.56, 0.78)
- indirect		-0.40 (-1.2, 0.37)
- network		-0.10 (-0.65, 0.44)
Atorvastatin_20mg vs. Pravastatin_40mg	0.9385	
- direct		0.64 (-1.4, 2.6)
- indirect		0.72 (-0.060, 1.6)
- network		0.76 (-0.0053, 1.5)
Atorvastatin_20mg vs. Rosuvastatin_10mg	0.99125	
- direct		-0.068 (-2.1, 1.8)
- indirect		-0.056 (-2.0, 2.0)
- network		-0.025 (-1.2, 1.2)
Atorvastatin_20mg vs. Simvastatin_20mg	0.31125	

- direct		0.15 (-1.0, 1.3)
- indirect		0.88 (0.12, 1.7)
- network		0.69 (0.057, 1.3)
Atorvastatin_40mg vs. Atorvastatin_80mg	0.98675	
- direct		-0.12 (-0.71, 0.46)
- indirect		-0.10 (-0.81, 0.63)
- network		-0.18 (-0.71, 0.43)
Atorvastatin_40mg vs. Placebo	0.83375	
- direct		2.4 (-0.35, 5.2)
- indirect		2.1 (1.5, 2.7)
- network		2.1 (1.5, 2.6)
Atorvastatin_40mg vs. Simvastatin_20mg	0.5855	
- direct		1.4 (-1.5, 3.6)
- indirect		0.58 (-0.093, 1.3)
- network		0.63 (-0.037, 1.3)
Atorvastatin_80mg vs. Placebo	0.22375	
- direct		2.4 (1.8, 3.0)
- indirect		1.8 (1.0, 2.6)
- network		2.2 (1.7, 2.7)
Placebo vs. Pravastatin_10mg	0.831	
- direct		-0.64 (-3.5, 2.4)
- indirect		-0.94 (-1.7, -0.085)
- network		-0.95 (-1.7, -0.11)
Placebo vs. Pravastatin_40mg	0.886	
- direct		-1.5 (-2.1, -0.75)
- indirect		-1.3 (-3.2, 0.39)
- network		-1.4 (-1.9, -0.79)
Placebo vs. Rosuvastatin_10mg	0.7035	
- direct		-1.6 (-3.9, 0.74)
- indirect		-2.2 (-3.5, -0.77)
- network		-2.1 (-3.3, -0.94)
Placebo vs. Rosuvastatin_5mg	0.4535	

- direct		-1.2 (-3.7, 1.3)
- indirect		-2.2 (-3.3, -1.4)
- network		-2.2 (-2.9, -1.3)
Placebo vs. Simvastatin_10mg	0.535	
- direct		-1.5 (-2.1, -0.85)
- indirect		-2.0 (-3.7, -0.24)
- network		-1.6 (-2.1, -1.0)
Placebo vs. Simvastatin_20mg	0.5375	
- direct		-1.5 (-2.8, -0.88)
- indirect		-1.8 (-2.9, -0.72)
- network		-1.4 (-1.9, -0.97)
Placebo vs. Simvastatin_80mg	0.777	
- direct		-2.4 (-3.2, -1.5)
- indirect		-1.9 (-4.7, 0.66)
- network		-2.3 (-3.1, -1.7)
Pravastatin_10mg vs. Rosuvastatin_5mg	0.71925	
- direct		-1.2 (-2.7, -0.50)
- indirect		-0.52 (-4.2, 3.5)
- network		-1.2 (-1.9, -0.52)
Pravastatin_20mg vs. Pravastatin_40mg	0.539	
- direct		-0.21 (-2.5, 2.1)
- indirect		-0.98 (-1.9, -0.070)
- network		-0.82 (-1.7, 0.041)
Pravastatin_20mg vs. Simvastatin_10mg	0.9555	
- direct		-0.70 (-1.7, 0.29)
- indirect		-0.74 (-2.7, 0.94)
- network		-1.0 (-1.8, -0.22)
Pravastatin_40mg vs. Rosuvastatin_10mg	0.96175	
- direct		-0.70 (-2.6, 1.1)
- indirect		-0.63 (-2.8, 1.3)
- network		-0.77 (-2.1, 0.51)
Pravastatin_40mg vs. Simvastatin_20mg	0.628	

- direct		-0.063 (-0.77, 0.64)
- indirect		-0.45 (-1.8, 1.0)
- network		-0.059 (-0.67, 0.52)
Rosuvastatin_10mg vs. Rosuvastatin_5mg	0.7785	
- direct		0.30 (-2.1, 2.6)
- indirect		-0.087 (-1.6, 1.5)
- network		-0.022 (-1.3, 1.3)
Rosuvastatin_10mg vs. Simvastatin_20mg	0.94975	
- direct		0.56 (-1.5, 2.4)
- indirect		0.69 (-1.3, 2.9)
- network		0.70 (-0.49, 1.9)
Simvastatin_10mg vs. Simvastatin_20mg	0.26325	
- direct		0.38 (-0.41, 1.2)
- indirect		-0.30 (-1.3, 0.60)
- network		0.14 (-0.45, 0.71)
Simvastatin_10mg vs. Simvastatin_40mg	0.84725	
- direct		-0.24 (-0.98, 0.49)
- indirect		-0.40 (-2.3, 1.1)
- network		-0.31 (-0.96, 0.33)
Simvastatin_10mg vs. Simvastatin_80mg	0.82075	
- direct		-0.65 (-1.4, 0.13)
- indirect		-0.97 (-3.8, 1.7)
- network		-0.74 (-1.5, -0.071)
Simvastatin_20mg vs. Simvastatin_40mg	0.48075	
- direct		-0.62 (-1.4, 0.18)
- indirect		-0.0078 (-1.5, 1.5)
- network		-0.45 (-1.1, 0.19)
Simvastatin_20mg vs. Simvastatin_80mg	0.75175	
- direct		-1.0 (-1.9, -0.23)
- indirect		-0.55 (-3.3, 2.4)
- network		-0.89 (-1.6, -0.22)

Overall statin intensity model ignoring agent:

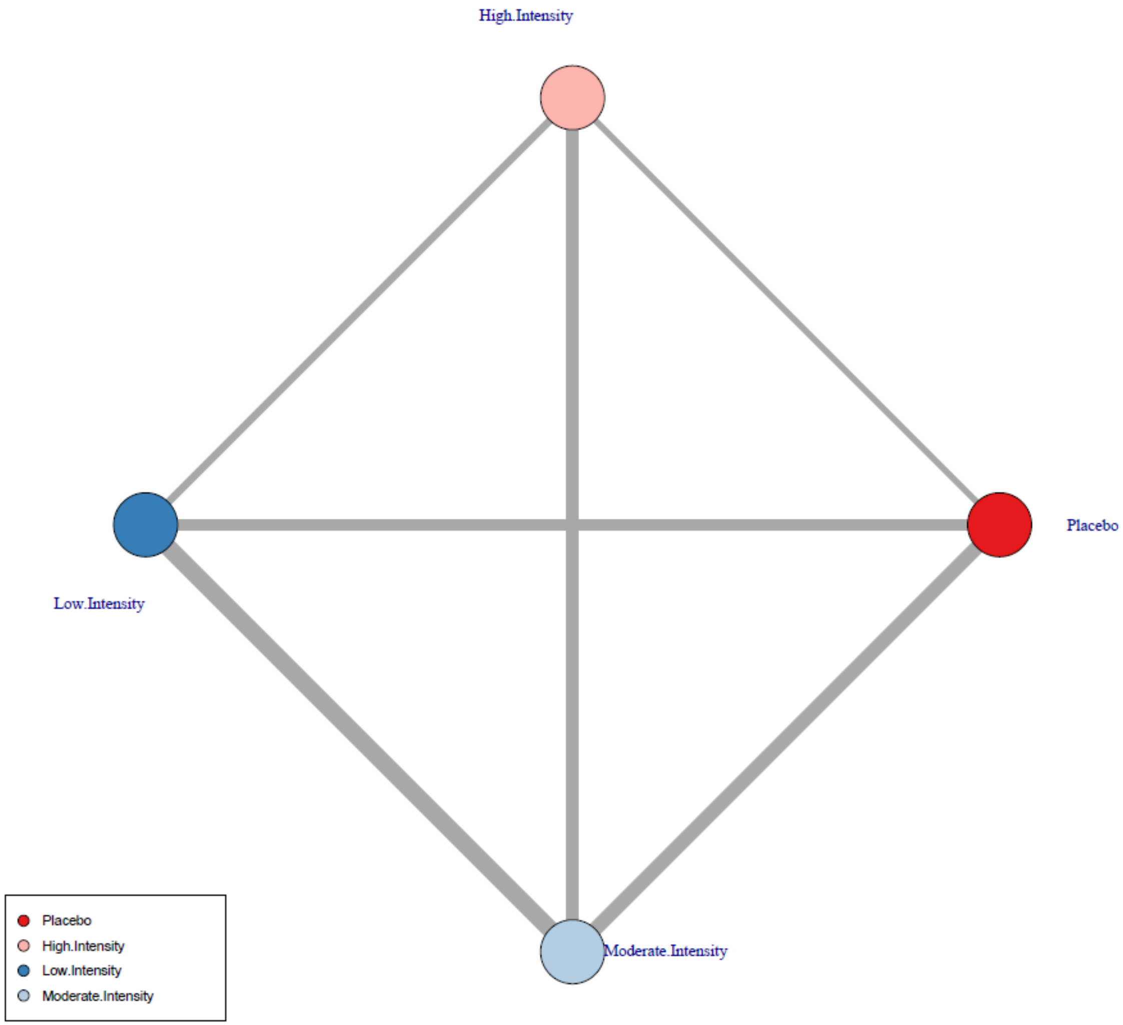

Forest plot of intensity model ignoring agent:

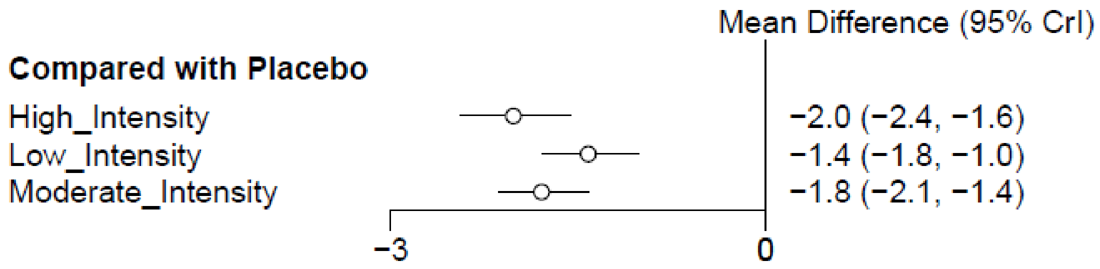

Node-split inconsistency analysis:

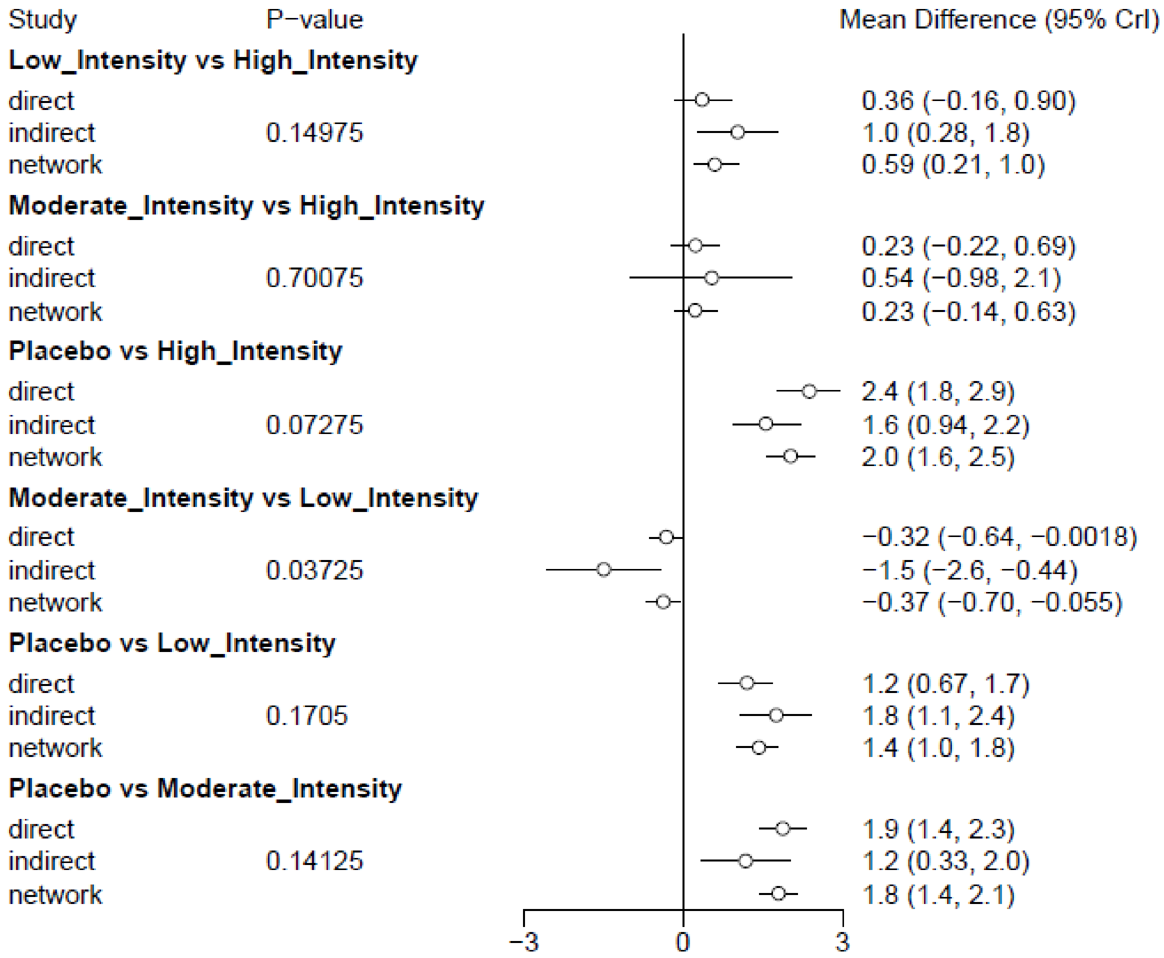

Appendix 14: Further Results for the Subgroup Analysis

League table 'heat plot' of head-to-head comparisons for patient risk subgroup analysis

Comparator \ Treatment	Rosuvastatin_mod_LR	Rosuvastatin_high_LR	Atorvastatin_high_LR	Simvastatin_high_LR	Atorvastatin_mod_LR	Atorvastatin_high_HR	Atorvastatin_low_LR	Simvastatin_mod_LR	Rosuvastatin_low_LR	Simvastatin_low_LR	Fluvastatin_mod_HR
Rosuvastatin_mod_LR		−0.04 (−1.25, 1.15)	0.08 (−0.77, 0.90)	0.06 (−1.02, 1.11)	0.18 (−0.59, 0.91)	0.35 (−1.97, 2.64)	0.37 (−0.48, 1.23)	0.55 (−0.33, 1.39)	0.73 (−1.40, 2.86)	0.68 (−0.21, 1.56)	0.89 (−2.18, 3.97)
Rosuvastatin_high_LR	0.04 (−1.15, 1.25)		0.12 (−1.01, 1.25)	0.09 (−1.21, 1.41)	0.22 (−0.88, 1.32)	0.39 (−2.05, 2.82)	0.41 (−0.72, 1.56)	0.59 (−0.56, 1.74)	0.78 (−1.37, 2.94)	0.72 (−0.45, 1.90)	0.93 (−2.23, 4.11)
Atorvastatin_high_LR	−0.08 (−0.90, 0.77)	−0.12 (−1.25, 1.01)		−0.02 (−0.92, 0.88)	0.10 (−0.37, 0.56)	0.27 (−1.97, 2.50)	0.29 (−0.23, 0.85)	0.47 (−0.20, 1.13)	0.66 (−1.50, 2.81)	0.60 (−0.06, 1.27)	0.81 (−2.21, 3.85)
Simvastatin_high_LR	−0.06 (−1.11, 1.02)	−0.09 (−1.41, 1.21)	0.02 (−0.88, 0.92)		0.13 (−0.73, 0.97)	0.30 (−2.01, 2.59)	0.32 (−0.57, 1.23)	0.50 (−0.29, 1.26)	0.68 (−1.57, 2.93)	0.62 (−0.17, 1.44)	0.83 (−2.23, 3.92)
Atorvastatin_mod_LR	−0.18 (−0.91, 0.59)	−0.22 (−1.32, 0.88)	−0.10 (−0.56, 0.37)	−0.13 (−0.97, 0.73)		0.17 (−2.05, 2.38)	0.19 (−0.29, 0.72)	0.37 (−0.22, 0.96)	0.56 (−1.57, 2.69)	0.50 (−0.09, 1.12)	0.71 (−2.29, 3.74)
Atorvastatin_high_HR	−0.35 (−2.64, 1.97)	−0.39 (−2.82, 2.05)	−0.27 (−2.50, 1.97)	−0.30 (−2.59, 2.01)	−0.17 (−2.38, 2.05)		0.03 (−2.20, 2.28)	0.20 (−2.01, 2.42)	0.39 (−2.68, 3.43)	0.33 (−1.89, 2.58)	0.54 (−3.13, 4.22)
Atorvastatin_low_LR	−0.37 (−1.23, 0.48)	−0.41 (−1.56, 0.72)	−0.29 (−0.85, 0.23)	−0.32 (−1.23, 0.57)	−0.19 (−0.72, 0.29)	−0.03 (−2.28, 2.20)		0.18 (−0.52, 0.84)	0.36 (−1.80, 2.53)	0.30 (−0.31, 0.91)	0.51 (−2.51, 3.56)
Simvastatin_mod_LR	−0.55 (−1.39, 0.33)	−0.59 (−1.74, 0.56)	−0.47 (−1.13, 0.20)	−0.50 (−1.26, 0.29)	−0.37 (−0.96, 0.22)	−0.20 (−2.42, 2.01)	−0.18 (−0.84, 0.52)		0.19 (−1.97, 2.35)	0.13 (−0.48, 0.77)	0.34 (−2.66, 3.37)
Rosuvastatin_low_LR	−0.73 (−2.86, 1.40)	−0.78 (−2.94, 1.37)	−0.66 (−2.81, 1.50)	−0.68 (−2.93, 1.57)	−0.56 (−2.69, 1.57)	−0.39 (−3.43, 2.68)	−0.36 (−2.53, 1.80)	−0.19 (−2.35, 1.97)		−0.06 (−2.23, 2.12)	0.16 (−3.51, 3.80)
Simvastatin_low_LR	−0.68 (−1.56, 0.21)	−0.72 (−1.90, 0.45)	−0.60 (−1.27, 0.06)	−0.62 (−1.44, 0.17)	−0.50 (−1.12, 0.09)	−0.33 (−2.58, 1.89)	−0.30 (−0.91, 0.31)	−0.13 (−0.77, 0.48)	0.06 (−2.12, 2.23)		0.21 (−2.81, 3.25)
Fluvastatin_mod_HR	−0.89 (−3.97, 2.18)	−0.93 (−4.11, 2.23)	−0.81 (−3.85, 2.21)	−0.83 (−3.92, 2.23)	−0.71 (−3.74, 2.29)	−0.54 (−4.22, 3.13)	−0.51 (−3.56, 2.51)	−0.34 (−3.37, 2.66)	−0.16 (−3.80, 3.51)	−0.21 (−3.25, 2.81)	
Simvastatin_low_HR	−1.08 (−3.49, 1.36)	−1.12 (−3.66, 1.43)	−1.00 (−3.36, 1.37)	−1.02 (−3.44, 1.42)	−0.90 (−3.25, 1.45)	−0.73 (−3.06, 1.57)	−0.70 (−3.07, 1.68)	−0.53 (−2.88, 1.83)	−0.34 (−3.48, 2.79)	−0.40 (−2.76, 1.98)	−0.18 (−3.92, 3.56)
Pitavastatin_mod_HR	−1.17 (−4.20, 1.88)	−1.21 (−4.34, 1.93)	−1.09 (−4.08, 1.90)	−1.12 (−4.15, 1.94)	−0.99 (−3.97, 1.99)	−0.83 (−3.88, 2.26)	−0.80 (−3.79, 2.21)	−0.62 (−3.60, 2.37)	−0.43 (−4.07, 3.16)	−0.49 (−3.47, 2.52)	−0.28 (−4.46, 3.88)
Atorvastatin_mod_HR	−1.10 (−3.10, 0.90)	−1.15 (−3.28, 1.00)	−1.02 (−2.94, 0.90)	−1.05 (−3.05, 0.95)	−0.92 (−2.83, 0.97)	−0.75 (−2.79, 1.27)	−0.72 (−2.66, 1.20)	−0.56 (−2.46, 1.35)	−0.37 (−3.17, 2.43)	−0.42 (−2.35, 1.50)	−0.21 (−3.70, 3.28)
Pravastatin_mod_LR	−1.21 (−3.34, 0.93)	−1.25 (−3.53, 1.04)	−1.13 (−3.22, 0.95)	−1.15 (−3.32, 1.00)	−1.03 (−3.10, 1.02)	−0.86 (−3.84, 2.10)	−0.83 (−2.93, 1.26)	−0.66 (−2.74, 1.41)	−0.47 (−3.41, 2.44)	−0.53 (−2.62, 1.56)	−0.32 (−3.90, 3.30)
Pravastatin_low_HR	−1.37 (−4.38, 1.62)	−1.41 (−4.51, 1.67)	−1.30 (−4.24, 1.63)	−1.32 (−4.32, 1.65)	−1.19 (−4.12, 1.72)	−1.03 (−4.61, 2.53)	−1.00 (−3.95, 1.95)	−0.83 (−3.76, 2.10)	−0.64 (−4.24, 2.94)	−0.69 (−3.64, 2.24)	−0.49 (−4.60, 3.65)
Atorvastatin_low_HR	−1.29 (−3.86, 1.27)	−1.33 (−4.01, 1.32)	−1.22 (−3.72, 1.27)	−1.24 (−3.81, 1.31)	−1.11 (−3.61, 1.36)	−0.94 (−3.29, 1.35)	−0.92 (−3.43, 1.58)	−0.74 (−3.24, 1.72)	−0.55 (−3.81, 2.66)	−0.61 (−3.13, 1.88)	−0.39 (−4.24, 3.40)
Pravastatin_low_LR	**−1.16** (−1.91, −0.41)	**−1.20** (−2.32, −0.09)	**−1.09** (−1.72, −0.47)	**−1.11** (−1.98, −0.26)	**−0.98** (−1.52, −0.48)	−0.82 (−3.04, 1.39)	**−0.79** (−1.43, −0.14)	**−0.61** (−1.21, −0.05)	−0.43 (−2.57, 1.71)	−0.49 (−1.12, 0.16)	−0.27 (−3.28, 2.75)
Simvastatin_mod_HR	−1.72 (−4.60, 1.18)	−1.76 (−4.74, 1.23)	−1.64 (−4.46, 1.20)	−1.67 (−4.53, 1.23)	−1.54 (−4.35, 1.29)	−1.37 (−3.84, 1.09)	−1.35 (−4.17, 1.51)	−1.17 (−3.98, 1.66)	−0.98 (−4.50, 2.50)	−1.05 (−3.86, 1.80)	−0.83 (−4.88, 3.24)
Fluvastatin_low_LR	**−2.13** (−3.67, −0.57)	**−2.17** (−3.90, −0.44)	**−2.05** (−3.48, −0.62)	**−2.08** (−3.61, −0.53)	**−1.95** (−3.36, −0.55)	−1.78 (−4.33, 0.74)	**−1.76** (−3.20, −0.30)	**−1.58** (−2.99, −0.17)	−1.40 (−3.91, 1.12)	**−1.45** (−2.88, −0.00)	−1.24 (−4.49, 2.02)
Placebo	**−2.31** (−3.08, −1.50)	**−2.34** (−3.45, −1.24)	**−2.23** (−2.76, −1.68)	**−2.25** (−3.02, −1.47)	**−2.12** (−2.59, −1.66)	−1.95 (−4.13, 0.20)	**−1.93** (−2.50, −1.33)	**−1.76** (−2.23, −1.28)	−1.57 (−3.70, 0.56)	**−1.63** (−2.16, −1.06)	−1.42 (−4.38, 1.57)
Fluvastatin_low_HR	**−2.91** (−5.77, −0.03)	−2.95 (−5.93, 0.01)	**−2.83** (−5.63, −0.02)	−2.86 (−5.70, 0.01)	−2.73 (−5.52, 0.07)	−2.57 (−6.04, 0.96)	−2.54 (−5.34, 0.28)	−2.36 (−5.14, 0.44)	−2.18 (−5.65, 1.32)	−2.23 (−5.03, 0.59)	−2.02 (−6.06, 2.07)

Comparator \ Treatment	Simvastatin_low_HR	Pitavastatin_mod_HR	Atorvastatin_mod_HR	Pravastatin_mod_LR	Pravastatin_low_HR	Atorvastatin_low_HR	Pravastatin_low_LR	Simvastatin_mod_HR	Fluvastatin_low_LR	Placebo	Fluvastatin_low_HR
Rosuvastatin_mod_LR	1.08 (−1.36, 3.49)	1.17 (−1.88, 4.20)	1.10 (−0.90, 3.10)	1.21 (−0.93, 3.34)	1.37 (−1.62, 4.38)	1.29 (−1.27, 3.86)	**1.16** (0.41, 1.91)	1.72 (−1.18, 4.60)	**2.13** (0.57, 3.67)	**2.31** (1.50, 3.08)	**2.91** (0.03, 5.77)
Rosuvastatin_high_LR	1.12 (−1.43, 3.66)	1.21 (−1.93, 4.34)	1.15 (−1.00, 3.28)	1.25 (−1.04, 3.53)	1.41 (−1.67, 4.51)	1.33 (−1.32, 4.01)	**1.20** (0.09, 2.32)	1.76 (−1.23, 4.74)	**2.17** (0.44, 3.90)	**2.34** (1.24, 3.45)	2.95 (−0.01, 5.93)
Atorvastatin_high_LR	1.00 (−1.37, 3.36)	1.09 (−1.90, 4.08)	1.02 (−0.90, 2.94)	1.13 (−0.95, 3.22)	1.30 (−1.63, 4.24)	1.22 (−1.27, 3.72)	**1.09** (0.47, 1.72)	1.64 (−1.20, 4.46)	**2.05** (0.62, 3.48)	**2.23** (1.68, 2.76)	**2.83** (0.02, 5.63)
Simvastatin_high_LR	1.02 (−1.42, 3.44)	1.12 (−1.94, 4.15)	1.05 (−0.95, 3.05)	1.15 (−1.00, 3.32)	1.32 (−1.65, 4.32)	1.24 (−1.31, 3.81)	**1.11** (0.26, 1.98)	1.67 (−1.23, 4.53)	**2.08** (0.53, 3.61)	**2.25** (1.47, 3.02)	2.86 (−0.01, 5.70)
Atorvastatin_mod_LR	0.90 (−1.45, 3.25)	0.99 (−1.99, 3.97)	0.92 (−0.97, 2.83)	1.03 (−1.02, 3.10)	1.19 (−1.72, 4.12)	1.11 (−1.36, 3.61)	**0.98** (0.48, 1.52)	1.54 (−1.29, 4.35)	**1.95** (0.55, 3.36)	**2.12** (1.66, 2.59)	2.73 (−0.07, 5.52)
Atorvastatin_high_HR	0.73 (−1.57, 3.06)	0.83 (−2.26, 3.88)	0.75 (−1.27, 2.79)	0.86 (−2.10, 3.84)	1.03 (−2.53, 4.61)	0.94 (−1.35, 3.29)	0.82 (−1.39, 3.04)	1.37 (−1.09, 3.84)	1.78 (−0.74, 4.33)	1.95 (−0.20, 4.13)	2.57 (−0.96, 6.04)
Atorvastatin_low_LR	0.70 (−1.68, 3.07)	0.80 (−2.21, 3.79)	0.72 (−1.20, 2.66)	0.83 (−1.26, 2.93)	1.00 (−1.95, 3.95)	0.92 (−1.58, 3.43)	**0.79** (0.14, 1.43)	1.35 (−1.51, 4.17)	**1.76** (0.30, 3.20)	**1.93** (1.33, 2.50)	2.54 (−0.28, 5.34)
Simvastatin_mod_LR	0.53 (−1.83, 2.88)	0.62 (−2.37, 3.60)	0.56 (−1.35, 2.46)	0.66 (−1.41, 2.74)	0.83 (−2.10, 3.76)	0.74 (−1.72, 3.24)	**0.61** (0.05, 1.21)	1.17 (−1.66, 3.98)	**1.58** (0.17, 2.99)	**1.76** (1.28, 2.23)	2.36 (−0.44, 5.14)
Rosuvastatin_low_LR	0.34 (−2.79, 3.48)	0.43 (−3.16, 4.07)	0.37 (−2.43, 3.17)	0.47 (−2.44, 3.41)	0.64 (−2.94, 4.24)	0.55 (−2.66, 3.81)	0.43 (−1.71, 2.57)	0.98 (−2.50, 4.50)	1.40 (−1.12, 3.91)	1.57 (−0.56, 3.70)	2.18 (−1.32, 5.65)
Simvastatin_low_LR	0.40 (−1.98, 2.76)	0.49 (−2.52, 3.47)	0.42 (−1.50, 2.35)	0.53 (−1.56, 2.62)	0.69 (−2.24, 3.64)	0.61 (−1.88, 3.13)	0.49 (−0.16, 1.12)	1.05 (−1.80, 3.86)	**1.45** (0.00, 2.88)	**1.63** (1.06, 2.16)	2.23 (−0.59, 5.03)
Fluvastatin_mod_HR	0.18 (−3.56, 3.92)	0.28 (−3.88, 4.46)	0.21 (−3.28, 3.70)	0.32 (−3.30, 3.90)	0.49 (−3.65, 4.60)	0.39 (−3.40, 4.24)	0.27 (−2.75, 3.28)	0.83 (−3.24, 4.88)	1.24 (−2.02, 4.49)	1.42 (−1.57, 4.38)	2.02 (−2.07, 6.06)
Simvastatin_low_HR		0.10 (−2.66, 2.84)	0.02 (−1.47, 1.52)	0.13 (−2.92, 3.22)	0.29 (−3.35, 3.99)	0.22 (−1.24, 1.69)	0.08 (−2.26, 2.45)	0.64 (−1.94, 3.24)	1.05 (−1.59, 3.72)	1.22 (−1.07, 3.55)	1.83 (−1.73, 5.38)
Pitavastatin_mod_HR	−0.10 (−2.84, 2.66)		−0.07 (−2.37, 2.23)	0.04 (−3.53, 3.62)	0.21 (−3.88, 4.29)	0.12 (−2.78, 3.06)	−0.01 (−2.98, 2.99)	0.55 (−2.78, 3.87)	0.96 (−2.26, 4.20)	1.13 (−1.80, 4.09)	1.74 (−2.29, 5.72)
Atorvastatin_mod_HR	−0.02 (−1.52, 1.47)	0.07 (−2.23, 2.37)		0.11 (−2.62, 2.85)	0.27 (−3.10, 3.69)	0.19 (−1.61, 1.99)	0.06 (−1.84, 1.97)	0.62 (−1.78, 3.00)	1.03 (−1.23, 3.29)	1.20 (−0.64, 3.05)	1.81 (−1.51, 5.08)
Pravastatin_mod_LR	−0.13 (−3.22, 2.92)	−0.04 (−3.62, 3.53)	−0.11 (−2.85, 2.62)		0.17 (−3.36, 3.70)	0.09 (−3.10, 3.25)	−0.04 (−2.07, 1.98)	0.51 (−2.94, 3.96)	0.93 (−1.53, 3.35)	1.10 (−0.94, 3.12)	1.70 (−1.72, 5.12)
Pravastatin_low_HR	−0.29 (−3.99, 3.35)	−0.21 (−4.29, 3.88)	−0.27 (−3.69, 3.10)	−0.17 (−3.70, 3.36)		−0.07 (−3.84, 3.66)	−0.21 (−3.15, 2.71)	0.34 (−3.64, 4.29)	0.76 (−2.43, 3.93)	0.93 (−1.97, 3.81)	1.53 (−2.49, 5.52)
Atorvastatin_low_HR	−0.22 (−1.69, 1.24)	−0.12 (−3.06, 2.78)	−0.19 (−1.99, 1.61)	−0.09 (−3.25, 3.10)	0.07 (−3.66, 3.84)		−0.13 (−2.63, 2.35)	0.43 (−2.11, 2.97)	0.84 (−1.93, 3.61)	1.01 (−1.44, 3.44)	1.62 (−2.05, 5.25)
Pravastatin_low_LR	−0.08 (−2.45, 2.26)	0.01 (−2.99, 2.98)	−0.06 (−1.97, 1.84)	0.04 (−1.98, 2.07)	0.21 (−2.71, 3.15)	0.13 (−2.35, 2.63)		0.56 (−2.28, 3.37)	0.97 (−0.46, 2.37)	**1.14** (0.65, 1.61)	1.75 (−1.06, 4.54)
Simvastatin_mod_HR	−0.64 (−3.24, 1.94)	−0.55 (−3.87, 2.78)	−0.62 (−3.00, 1.78)	−0.51 (−3.96, 2.94)	−0.34 (−4.29, 3.64)	−0.43 (−2.97, 2.11)	−0.56 (−3.37, 2.28)		0.40 (−2.66, 3.50)	0.58 (−2.19, 3.38)	1.19 (−2.70, 5.08)
Fluvastatin_low_LR	−1.05 (−3.72, 1.59)	−0.96 (−4.20, 2.26)	−1.03 (−3.29, 1.23)	−0.93 (−3.35, 1.53)	−0.76 (−3.93, 2.43)	−0.84 (−3.61, 1.93)	−0.97 (−2.37, 0.46)	−0.40 (−3.50, 2.66)		0.17 (−1.15, 1.50)	0.78 (−2.31, 3.83)
Placebo	−1.22 (−3.55, 1.07)	−1.13 (−4.09, 1.80)	−1.20 (−3.05, 0.64)	−1.10 (−3.12, 0.94)	−0.93 (−3.81, 1.97)	−1.01 (−3.44, 1.44)	**−1.14** (−1.61, −0.65)	−0.58 (−3.38, 2.19)	−0.17 (−1.50, 1.15)		0.61 (−2.16, 3.35)
Fluvastatin_low_HR	−1.83 (−5.38, 1.73)	−1.74 (−5.72, 2.29)	−1.81 (−5.08, 1.51)	−1.70 (−5.12, 1.72)	−1.53 (−5.52, 2.49)	−1.62 (−5.25, 2.05)	−1.75 (−4.54, 1.06)	−1.19 (−5.08, 2.70)	−0.78 (−3.83, 2.31)	−0.61 (−3.35, 2.16)	

Forest plot of sensitivity analysis after removing high RoB studies

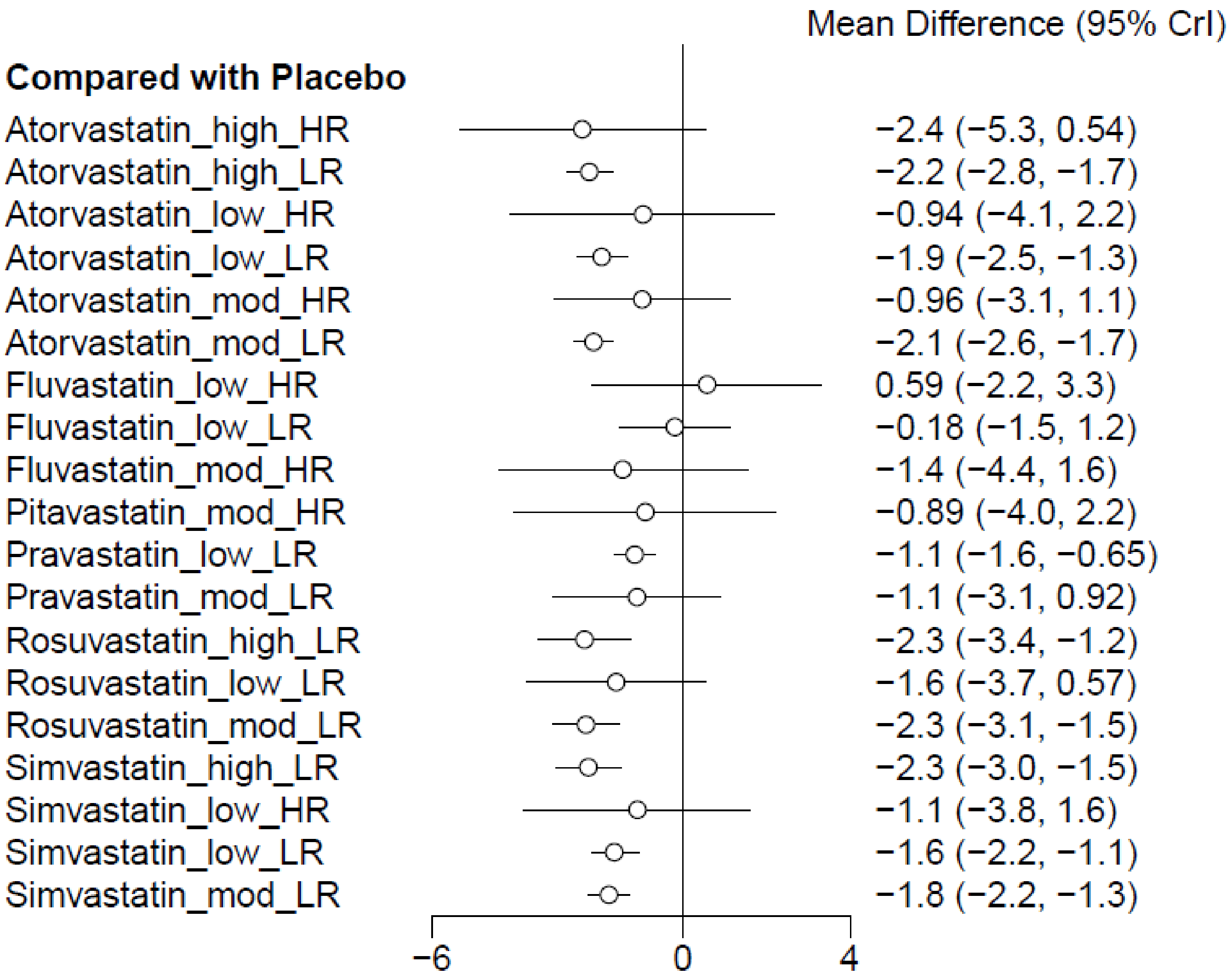

SUCRA rankings for sensitivity analysis

Treatment	SUCRA Score
Rosuvastatin_mod_LR	0.7889692
Rosuvastatin_high_LR	0.7808058
Atorvastatin_high_LR	0.7737345
Simvastatin_high_LR	0.7678061
Atorvastatin_mod_LR	0.7245301
Atorvastatin_high_HR	0.7040383
Atorvastatin_low_LR	0.6257478
Simvastatin_mod_LR	0.553373
Rosuvastatin_low_LR	0.5279125
Fluvastatin_mod_HR	0.4983751
Simvastatin_low_LR	0.4967534
Simvastatin_low_HR	0.4212047
Pravastatin_mod_LR	0.3988972
Atorvastatin_low_HR	0.3975071
Pitavastatin_mod_HR	0.3863496
Atorvastatin_mod_HR	0.3724859
Pravastatin_low_LR	0.347588
Fluvastatin_low_LR	0.1769425
Placebo	0.1299753
Fluvastatin_low_HR	0.1270038
